# Unveiling the Evolution of Afterglow in Diboraanthracene Scaffolds: From Thermally Activated Delayed Fluorescence to Room-Temperature Phosphorescence

**DOI:** 10.1021/jacs.5c16948

**Published:** 2025-11-28

**Authors:** Yi-Kuan Chen, Jian Lei, Po-Cheng Liu, Cheng-Han Lin, Yu-Ming Chen, Wun-Shuo Chang, I-Chia Chen, Liang-Yan Hsu, Tien-Lin Wu

**Affiliations:** † Department of Chemistry, 34881 National Tsing Hua University, No. 101, Sec. 2, Kuang-Fu Rd., Hsinchu 300044, Taiwan; ‡ Department of Public Foundation, 381933 Chongqing Three Gorges Medical College, Chongqing 404120, China; § 71556 Institute of Atomic and Molecular Sciences, Academia Sinica, Taipei 106319, Taiwan; ∥ Department of Chemistry, National Taiwan University, No. 1, Sec. 4, Roosevelt Rd., Taipei 106319, Taiwan; ⊥ Physics Division, National Center for Theoretical Sciences, No. 1, Sec. 4, Roosevelt Rd., Taipei 106319, Taiwan; # College of Semiconductor Research, 34881 National Tsing Hua University, No. 101, Sec. 2, Kuang-Fu Rd., Hsinchu 300044, Taiwan

## Abstract

Three-coordinate organoboranes have emerged as promising afterglow emitters by promoting intersystem crossing (ISC) through the introduction of boron centers. Among them, diboraanthracene (DBA) derivatives have recently shown great potential for achieving afterglow with second-scale durations. This study explores how the structural modulation of DBA scaffolds governs afterglow evolution. For the first time, ultralong thermally activated delayed fluorescence (TADF) was identified from 9,10-dimesityl-9,10-dihydro-9,10-diboraanthracene (MesDBA), which exhibits a delayed lifetime of 0.72 s and represents the longest pure TADF reported to date. Building on this insight, π-conjugation was extended to yield 6,13-dimesityl-6,13-dihydro-6,13-diborapentacene (MesDBP), which unlocks a new pathway for room-temperature phosphorescence (RTP) with a duration of 12 s and a lifetime of 1.41 s. An iptycene-derived DBA, mesityldiborapentiptycene (MesDBPI), was designed to modulate excited-state dynamics, affording a hybrid TADF-RTP afterglow lasting up to 40 s. Its deuterated analogue, MesDBPI-*d*_18_, further extended the lifetimes to 4.00 s (TADF) and 4.22 s (RTP), establishing the longest values among organoboron emitters in an inert polymer. To rationalize these findings, a theoretical model grounded in Marcus theory was employed to predict the reverse intersystem crossing (RISC) rate constants, showing strong agreement with experimental measurements across all compounds. Furthermore, afterglow organic light-emitting diodes (OLEDs) based on MesDBPI achieved an external quantum efficiency (EQE) of up to 1.8%, demonstrating the validity of this molecular design strategy at the device level. In addition, color-tunable afterglows enable the demonstration of multilevel security applications. This controllable evolution from TADF to RTP underscores the versatility of DBA frameworks as a robust platform for next-generation optoelectronics and security technologies.

## Graphical abstract

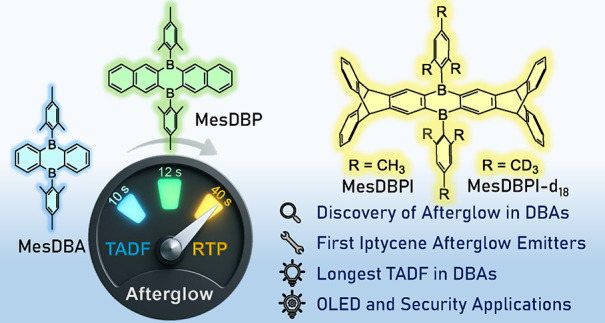

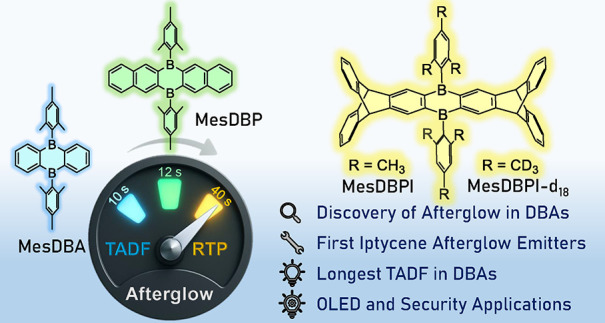

## Introduction

Afterglow is the emission with a lifetime exceeding 0.1 s after the excitation source is turned off.^1^ The study of afterglow can be traced back to the development of inorganic phosphors, such as Eu^2+^/Dy^3+^-doped SrAl_2_O_4_, reported in 1996.^2^ Nowadays, those mature inorganic materials with persistent luminescence are applied for several commercial products, such as luminous paints, luminous toys, emergency signs, etc. In contrast, organic afterglow studies are an emerging research area,^3^ primarily driven by ultralong phosphorescence at room temperature. However, purely organic compounds generally suffer from inefficient intersystem crossing (ISC) and spin-forbidden radiative transitions from the triplet state, which significantly limit their ability to emit room-temperature phosphorescence (RTP) under ambient conditions. To overcome these challenges, various molecular strategies have been developed to stabilize triplet excited states and suppress nonradiative decay, including crystalline design,^4,5^ strong π–π interaction,^6^ halogen bonding,^7^ isomer impurity,^8,9^ nonplanar geometry,^10^ matrix engineering,^11^ and host–guest systems.^12,13^ Among those methods, an effective molecular design strategy is well-recognized to promote ultralong RTP via incorporating amine or carbonyl functional groups, such as carbazole, phenothiazine, and ketones.^14^ These groups facilitate the ISC process through their lone-pair electrons, effectively enhancing triplet exciton generation and increasing overall triplet exciton density, thereby contributing to afterglow.^15^ In recent years, ultralong thermally activated delayed fluorescence (TADF) represents an important class of afterglow emissions. A foundational study by Prof. Zhang and co-workers in 2021 demonstrated a difluoroboron-based TADF material exhibiting afterglow with a delayed lifetime of 0.31 s.^16^ The relatively wide singlet–triplet gap (Δ*E*_ST_ = 0.22 eV) led to a slow reverse intersystem crossing (RISC) process (rate constant, *k*_RISC_ ≈ 10^0^–10^1^ s^–1^). The TADF system was subsequently optimized to achieve a prolonged lifetime (0.47 s)^17^ and a narrowband afterglow.^18,19^ Subsequently, ultralong TADF materials have also been integrated into organic long-persistent luminescence (OLPL) systems, enabling remarkable afterglow with an hour-long duration.^20,21^ On the other hand, Kabe et al. reported the pioneering work on fabricating afterglow organic light-emitting diodes (OLEDs) via an amine-type emitter (DMFLTPD) and a deuterated coronene in 2015.^22^ Later, an OLPL system^12,23^ was employed to achieve persistent electroluminescence (EL) after the power was off.^24^ Recently, a combination of 2,8-bis­(diphenylphos-phoryl)­dibenzo­[*b,d*]­thiophene (PPT) and *N*,*N*′-di­(1-naphthyl)-*N*,*N*′-diphenyl-(1,10-biphenyl)-4,4′-diamine (NPB) achieved afterglow OLEDs with low driving voltage and a relatively high external quantum efficiency (EQE) of 1.47%.^25^ Despite the recent advances, the availability of high-performance afterglow dopants for practical optoelectronic applications remains limited, as critical parameters such as EQE and brightness continue to lag behind practical performance benchmarks.^22,25,26^ Thus, the exploration of novel molecular scaffolds and rational design strategies are essential for addressing these limitations and advancing functional afterglow materials.

Organoboron compounds have emerged as a highly promising class of materials among organic RTP emitters, driven by the distinctive electronic properties of boron.^27^ Their significance stems from the relatively rare efficient organic afterglow emitters, alongside recent discovery and development of boron-containing systems. There are several crystalline derivatives of phenylboronic acids and esters showing the ultralong RTP with longer lifetime (>1 s) earlier.^28,29^ However, the mechanisms of such boronic acids are still attributed to the lone pairs,^30^ intermolecular interactions,^31^ halogen doping,^7,14^ and even impurity.^32,33^ In 2020, Wu et al. reported a series of triphenylboranes exhibited the RTP property.^34^ These three-coordinated organoboranes are a new type of ultralong afterglow fluorophores without n−π* transitions. The vacant p orbital of the boron atom plays a critical role in facilitating intersystem crossing (ISC), thereby contributing to the formation of persistent RTP. Based on literature emission spectra, the difference (Δ*E*_ST_) between singlet and triplet excited states is estimated to be greater than 1.0 eV. The discovery inspired the following molecular design using triarylboranes to investigate the ultralong RTP. The incorporation of a dimethylamine group into triarylboranes can generate additional charge transfer transition to achieve both ultralong TADF and RTP in a PMMA matrix.^35,36^ Furthermore, Jovaišaitė et al. discovered diboraanthracene (DBA) derivatives with longer afterglow behavior and further developed them for data recording applications.^37^ The hybrid afterglow (combining TADF and RTP) exhibited by these DBA derivatives in PMMA captured our attention and sparked further in-depth exploration.

The concept of boron-doped polycyclic aromatic hydrocarbons (PAHs) dates back to early studies in the 1970s,^38,39^ motivated by the desire to tailor electronic properties through heteroatom substitution. However, it was not until the 2000s that stable, isolable boracycles were successfully synthesized,^40^ driven by synthetic advances in organoboron chemistry.^41,42^ Among these developments, diboron-embedded aromatics have emerged as a particularly intriguing class, offering unique opportunities for modulating electronic structures and reactivities compared to their all carbon analogues.^43–45^ Notably, DBA compounds, featuring two boron atoms embedded in the anthracene backbone, offer a versatile platform for tuning molecular properties. Pioneering work by Prof. Wagner’s group established synthetic access to a wide range of functionalized DBA derivatives for comprehensive studies, including air and water stability,^46^ substituent effects,^47,48^ π-extension,^49,50^ reactivities,^51,52^ and photophysical properties.^53–55^ Researchers have leveraged the insights, and DBA derivatives have attracted growing interest as promising candidates for OLED applications. In 2018, the first efficient DBA-based TADF OLEDs demonstrated benchmark performance, attributed to the incorporation of electron-donating groups and rod-like molecular architectures.^56^ Subsequent advances enabled single-layer OLEDs,^57,58^ an ultrathin emitting layer,^59^ a white OLED,^60^ and devices with extended operational lifetimes.^57,60^ Yet, despite these impressive advancements, a critical gap remains in our understanding of the fundamental DBA systems themselves. Among the reported DBA derivatives, 9,10-dimesityl-9,10-dihydro-9,10-diboraanthracene (MesDBA) is the simplest air- and water-stable molecule, first documented in 1995.^61^ Despite its three-decade history and frequent appearance in the literature,^45,46,62^ detailed investigations into its photophysical properties have been surprisingly limited with only basic steady-state PL spectra reported, thereby treating it as a conventional fluorescent molecule.

Motivated by the overlooked potential of MesDBA, we revisited its emission behavior from a deeper perspective. Remarkably, this study uncovers for the first time that MesDBA exhibits pure and ultralong TADF, a phenomenon unprecedented among pristine arylboranes (Scheme 1). Its Δ*E*_ST_ value of 0.36 eV is much lower than the above-mentioned triarylborane species with long-lived RTP.^34^ To clarify the effect of boron substitution, we further examined the boron-free analogue 9,10-dimesitylanthracene (MesAn), which exhibits only prompt fluorescence.^53^ This comparison illustrates how boron doping reshapes excited-state dynamics, offering a design concept for diverse π-conjugated systems. Similarly, we reveal that 6,13-dimesityl-6,13-dihydro-6,13-diborapentacene (MesDBP)^53,63^ shows distinct afterglow characteristics with an additional and prolonged RTP component, further demonstrating the critical role of π-extension in tuning excited-state dynamics. This discovery expands our understanding of its photophysical behavior and provides new insights into the molecular design for boron-induced afterglow. To elucidate the afterglow mechanism and improve EL performance, we designed and synthesized mesityldiborapentiptycene (MesDBPI), a new DBA derivative. The triptycene scaffolds have been introduced into TADF emitters previously,^64–68^ and their trigonal shapes not only provide the rigidity and three-dimensional steric hindrance^69^ for preventing the emission aggregation caused quenching (ACQ) but also extend the delocalization region by the homoconjugation effect.^70,71^ Recent reports have shown that triptycene-derived materials can form thermally stable films with excellent morphological stability.^72,73^ In Scheme 1, MesDBPI contains two triptycene units to extend the conjugation length and construct the pentiptycene-derived diboron core, diborapentiptycene. This nonplanar and rigid emitter exhibits hybrid afterglow behavior, combining persistent TADF and RTP components with an emission duration of up to 14 s in the PMMA polymer. Further thermal annealing of this doped film dramatically pushes the afterglow duration to a notable 40 s with a lifetime of 3.70 s. In addition, site-selective deuteration of the mesityl rings in MesDBPI-*d*_18_ further extended the lifetimes to 4.00 s (TADF) and 4.22 s (RTP), representing the longest record for a single organoboron molecule in an inert polymer. A comprehensive understanding of the excited-state processes was pursued by using time-resolved spectroscopic techniques and theoretical calculations. In terms of molecular design, extending the π-conjugation in MesDBP and incorporating an iptycene unit in MesDBPI increase steric hindrance and introduce additional exciton transition pathways. This steric engineering isolates triplet excitons, thereby preventing intermolecular quenching, such as triplet–triplet annihilation (TTA), and enforcing specific intermolecular distances. Meanwhile, conformational locking further inhibits the nonradiative decay from the triplet excited state to the ground state. Moreover, methyl deuteration in MesDBPI-*d*_18_ suppresses high-frequency C–H vibrations, reducing nonradiative decay and enhancing afterglow lifetimes. This highlights the effectiveness of the design strategy in synergistically managing singlet and triplet radiative pathways and further unveils the critical role of the DBA core in enabling efficient ISC, slow RISC, and triplet-state stabilization without reliance on heavy atoms or matrix interaction. Consequently, MesDBPI was employed as a dopant emitter in afterglow OLEDs, achieving a superior EQE of 1.8% and an emission lifetime of 113 ms. By uncovering the mechanisms of long-lived emission, this study lays the groundwork for the rational design of advanced luminescent materials. The precisely tunable afterglow behavior of DBA-based systems highlights their potential in next-generation organic electronic and photonic security applications.

**Scheme 1:**
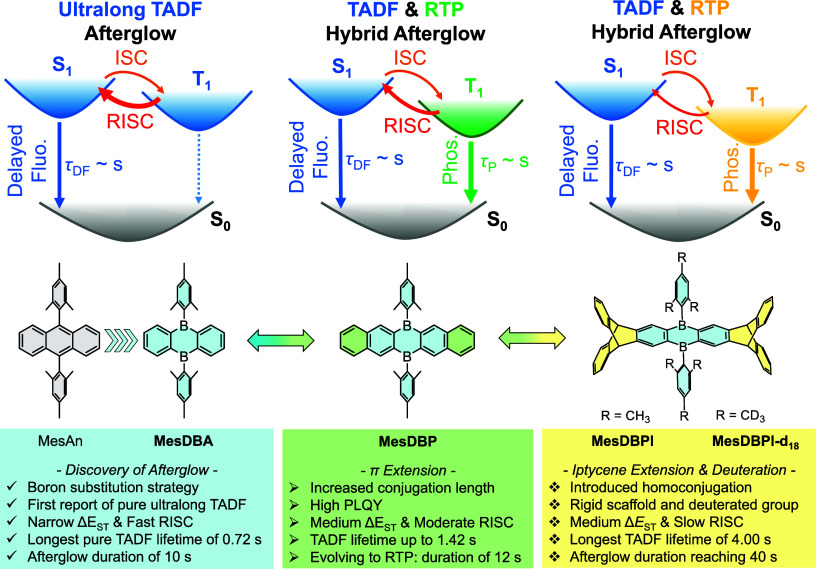
Schematic Illustration of Excited-State Pathways and Molecular Evolution in Three Types of Diboraanthracene Scaffolds

## Results and Discussion

### Materials Synthesis and Characterization

First, syntheses of 7,16-dimesityl-5,7,9,14,16,18-hexahydro-5,18:9,14-bis­([1,2]­benzeno)­dinaphtho­[2,3-b:2′,3′-i]­boranthren (MesDBPI) and its deuterated derivative MesDBPI-*d*_18_ are illustrated in Scheme 2. We prepared a starting material, 9,10-dihydro-2,3-bis­(trimethylsilyl)-9,10­[1′,2′]-benzenoanthracene,^74^ and stirred with boron tribromide in hexane for 3 days to afford the dibromodiborapentiptycene (DBDBPI). Next, we used 2-mesitylmagnesium bromide and 2-mesityl-*d*_9_magnesium bromide to react with DBDBPI, respectively, and obtained the desired products, MesDBPI and MesDBPI-*d*_18_. MesAn, MesDBA, and MesDBP were prepared according to previous literature.^45,53,75^ Details of the synthetic procedure, ^1^H/^13^C NMR, high-resolution mass spectrometry and elemental analysis, and IR spectroscopy are provided in the Supporting Information, as shown in Figures S1–S13. To investigate the influence of π-extension in the DBA system, the single crystals of MesDBP, MesDBPI, and MesDBPI-*d*_18_ (Figure S14) were prepared and determined by X-ray diffraction (XRD), and the corresponding parameters are summarized in Table S1. In Figure 1, the dihedral angles (α and β) of MesDBPI and MesDBPI-*d*_18_ between the phenyl ring of the DBPI core are 78.4/79.4° and 77.1/79.4°, respectively, while the dihedral angles of MesDBA are determined to be 86.6° and 87.7°. Moreover, we substituted triptycene units at the DBA core to induce steric hindrance with large angles (θ = 106°) and increase the intermolecular distances. The intermolecular distances of 5.59, 6.80, and 6.69 Å were found in the crystal packing of MesDBP, MesDBPI, and MesDBPI-*d*_18_, respectively, which are much longer than those of MesDBA (2.43–3.25 Å).^45^ Therefore, no intense intermolecular π–π stacking interactions were observed for MesDBP, MesDBPI, and MesDBPI-*d*_18_. Additionally, the theoretical root-mean-square deviation (RMSD) values between the ground (S_0_) and excited states (S_1_ and T_1_) in the crystal of MesDBPI/MesDBPI-*d*_18_ are the lowest, calculated at 0.041 and 0.037 Å, respectively. Similarly, MesDBA and MesDBP exhibit low RMSD values of 0.041/0.047 and 0.065/0.051 Å, respectively, indicating that MesDBPI/MesDBPI-*d*_18_ possesses greater structural rigidity compared to others. Based on these results, it is expected that intermolecular exciton distances of MesDBPI could be effectively increased by the pentiptycene-derived substructure, which could suppress triplet-related quenching processes and improve the performance of OLEDs. A cyclic voltammetry method was employed to determine the lowest unoccupied molecular orbital (LUMO) levels of MesDBA, MesDBP, MesDBPI, and MesDBPI-*d*_18_, as illustrated in Figure S15. The HOMO level was then calculated by subtracting the corresponding LUMO level and the optical bandgap (*E*_g_). Thus, the HOMO/LUMO energy levels of MesDBA, MesDBP, MesDBPI, and MesDBPI-*d*_18_ are determined to be −5.51/–2.68, −5.66/–2.70, −5.50/–2.65, and −5.48/–2.66 eV, respectively. Thermal gravimetric analysis (TGA) and differential scanning calorimetry (DSC) measurements under a nitrogen atmosphere were performed. As shown in Figure S16, MesDBA, MesDBP, MesDBPI, and MesDBPI-*d*_18_ exhibit excellent thermal stability with decomposition temperatures of 256, 298, 464, and 435 °C, respectively. Additionally, MesDBA shows a high glass-transition temperature of 235 °C. These favorable thermal properties indicate that all compounds are suitable for thermal evaporation in device fabrication.

**Scheme 2:**
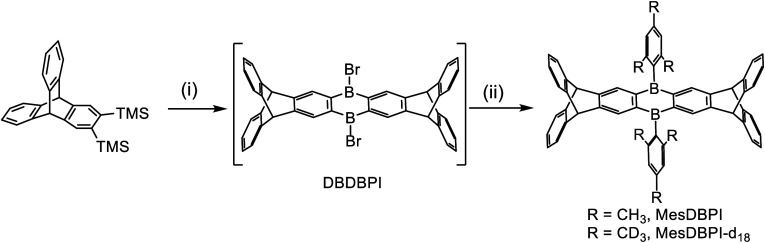
Synthesis of MesDBPI and MesDBPI-*d*_18_ ^a^

a Conditions: (i) BBr_3_, *n*-hexane, 120 °C, 3 d; (ii) 2-mesitylmagnesium bromide or 2-mesityl-d_9_magnesium bromide, toluene, 0 °C to room temperature, 18 h.

**Figure 1:**
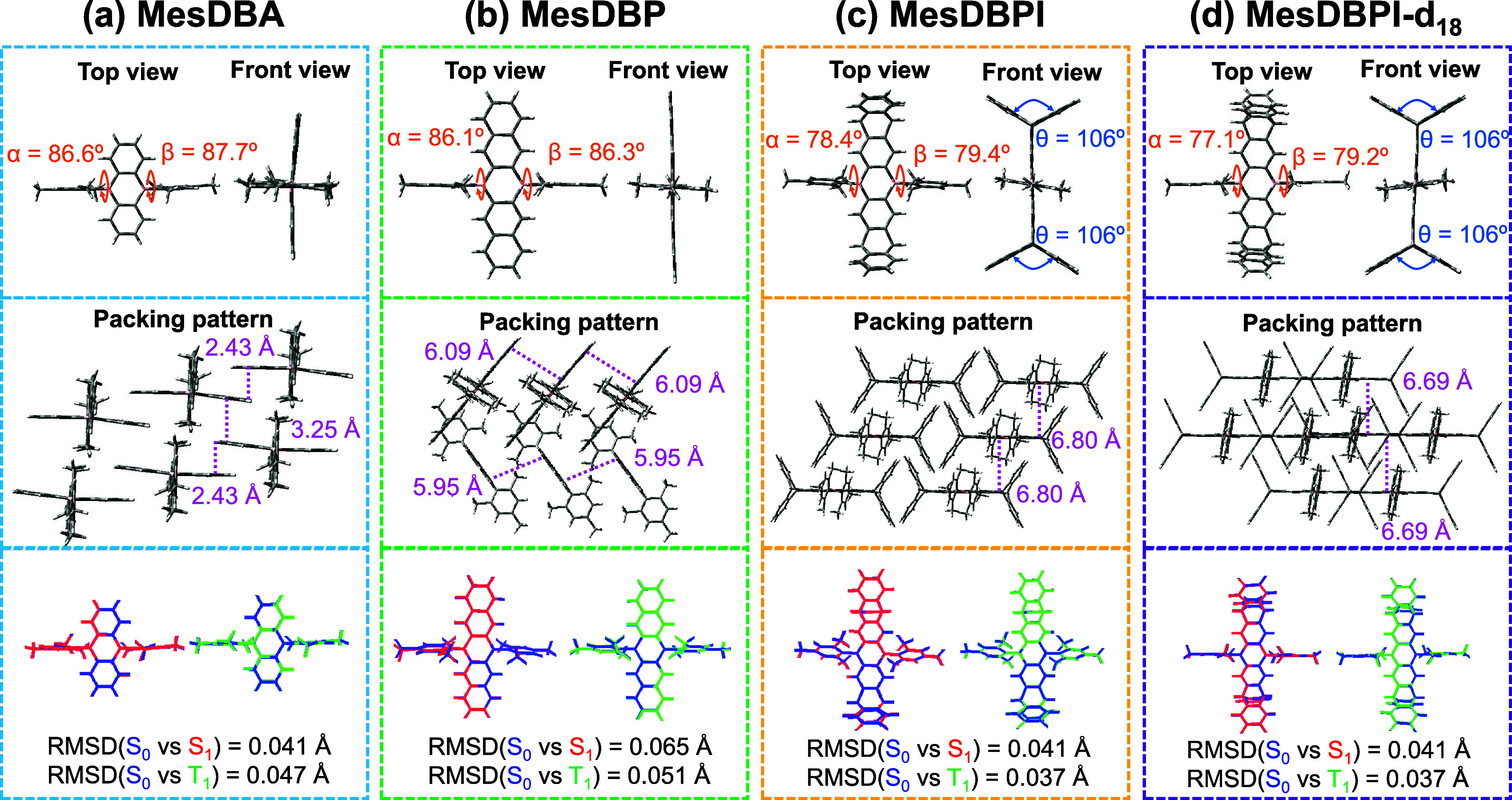
Single crystal structures, packing pattern diagram with relevant intermolecular interactions, and root-mean-square deviation (RMSD) values between S_0_ and S_1_/T_1_ geometries (calculated at the BMK/6-31G­(d) level in the solid phase) for (a) MesDBA, (b) MesDBP, (c) MesDBPI, and (d) MesDBPI-*d*_18_.

To investigate the photophysics of MesAn and diboron-based molecules, their UV/vis absorption and PL spectra in toluene (10^–4^ M) were measured, and the relevant photophysical data are summarized in Table S2. As shown in Figure 2a–d, all emitters showed strong absorption peaks at 350 nm for MesDBA, 304 nm for MesDBP, and 290 and 358 nm for MesDBPI and MesDBPI-*d*_18_, which belong to the π → π* transition. The weak and broad absorption bands of MesDBA, MesDBPI, and MesDBPI-*d*_18_ were observed at around 400 nm, which could be assigned to the charge-transfer (CT) transition. PL spectra of MesDBPI, MesDBPI-*d*_18_, and MesDBA showed structureless emission with peaks at 481, 482, and 455 nm, respectively, while MesDBP showed structured emission with peaks at 417, 441, and 469 nm. The fluorescence spectra of MesDBP, MesDBPI, and MesDBPI-*d*_18_ showed no pronounced solvatochromism in various polar solvents, whereas MesDBA displayed a clear bathochromic effect along with increasing solvent polarity (Figure S17). The full width at half maximum (FWHM) values of MesAn, MesDBA, MesDBP, MesDBPI, and MesDBPI-*d*_18_ in toluene are 16, 68, 12, 56, and 57 nm, respectively. Among them, MesDBP displays a sharp emission with well-resolved vibronic features, whereas MesDBA shows the broadest spectrum. These distinct solvatochromic responses and emission profiles indicate that MesDBP, MesDBPI, and MesDBPI-*d*_18_ undergo a dominant localized excited (LE) transition, while MesDBA primarily exhibits a CT transition. MesAn exhibited distinctly different behavior from other boron-containing species, as shown in Figure S18. Its absorption and emission spectra display clear vibrational structures and a mirror image, consistent with previous reports,^45^ indicating a purely LE character. We then investigated the photophysical properties of all emitters in PMMA at a doping concentration of 1 wt %. The fluorescence and phosphorescence spectra of the doped films were measured at room temperature and 100 K, respectively, and Δ*E*_ST_ was determined from the onset of the fluorescence and phosphorescence spectra (Figure 2e–h). The experimentally determined Δ*E*_ST_ values for MesDBA, MesDBP, MesDBPI, and MesDBPI-*d*_18_ were 0.36, 0.57, 0.52, and 0.53 eV in the solid state, respectively. Furthermore, Figure 3a displays photographs of all-emitter-doped PMMA on a quartz plate under 365 nm UV illumination. When the excitation source was turned off, a distinct sky-blue, green, and yellow afterglow was observed for MesDBA, MesDBP, and MesDBPI/MesDBPI-*d*_18_, respectively, highlighting their afterglow properties. The afterglow durations, defined as the time until the emission was no longer visible to the naked eye and confirmed by camera recordings, were 4 s for MesDBA, 7 s for MesDBP, and 14 s for MesDBPI and MesDBPI-*d*_18_, respectively. In contrast, MesAn exhibited no detectable long-lived emission. To ensure a fair comparison of afterglow durations, we performed control experiments varying the film thickness and excitation time (3, 10, and 30 s). Under all tested conditions for the MesDBPI sample, the afterglow duration remained largely unchanged (Figure S19), indicating that the property is intrinsic to the molecule and minimally influenced by these parameters. Notably, a milder annealing treatment (90 °C for 30 min), compared with the higher temperatures (140–250 °C) reported in previous studies,^37,76^ was applied to all samples. The process significantly enhanced the afterglow durations of all PMMA films, reaching 10 s for MesDBA, 12 s for MesDBP, and 40 s for MesDBPI and MesDBPI-*d*_18_ (Figure 3b). The enhanced afterglow observed after thermal annealing is likely associated with physical aging of the PMMA matrix.^77^ Annealing below PMMA’s *T*_g_ (∼378 K) induces structural relaxation, leading to denser chain packing, reduced free volume, and increased rigidity, which suppress nonradiative decay and improve oxygen barrier properties. In Figure S20, the disappearance of the “sea–island” pattern in the SEM images further supports chain reorganization toward a more homogeneous and stable state. Importantly, the 40 s afterglow achieved by MesDBPI and MesDBPI-*d*_18_ represents one of the longest durations reported for a single organic molecule in an inert matrix such as PMMA. Although defined only as the time visible to the naked eye, duration remains a practical metric, and our result exceeds previous reports typically limited to ∼35 s.^78^ This benchmark underscores the success of our molecular design in achieving long-lived excited states without relying on external heavy atoms and matrix-specific interactions.^79–81^ The emission spectra of all compounds broaden in the PMMA films, particularly for MesDBP, MesDBPI, and MesDBPI-*d*_18_. The increased FWHM values of the three molecules might be attributed to additional RTP components. Time-gated PL spectra recorded at various delay times (Figure 3c–f) further support this observation. MesDBP, MesDBPI, and MesDBPI-*d*_18_ exhibited a rising band around 500 to 700 nm after a 6 ms delay, closely resembling its low-temperature profile, confirming its RTP properties. In contrast, the PL spectrum of MesDBA remained nearly unchanged during this period, emitting a steady blue glow. A similar phenomenon was observed for those of thermally treated films, as shown in Figure S21. In short, the results suggest that MesDBA’s afterglow likely originates from TADF, while the others may contain additional RTP contributions.

**Figure 2:**
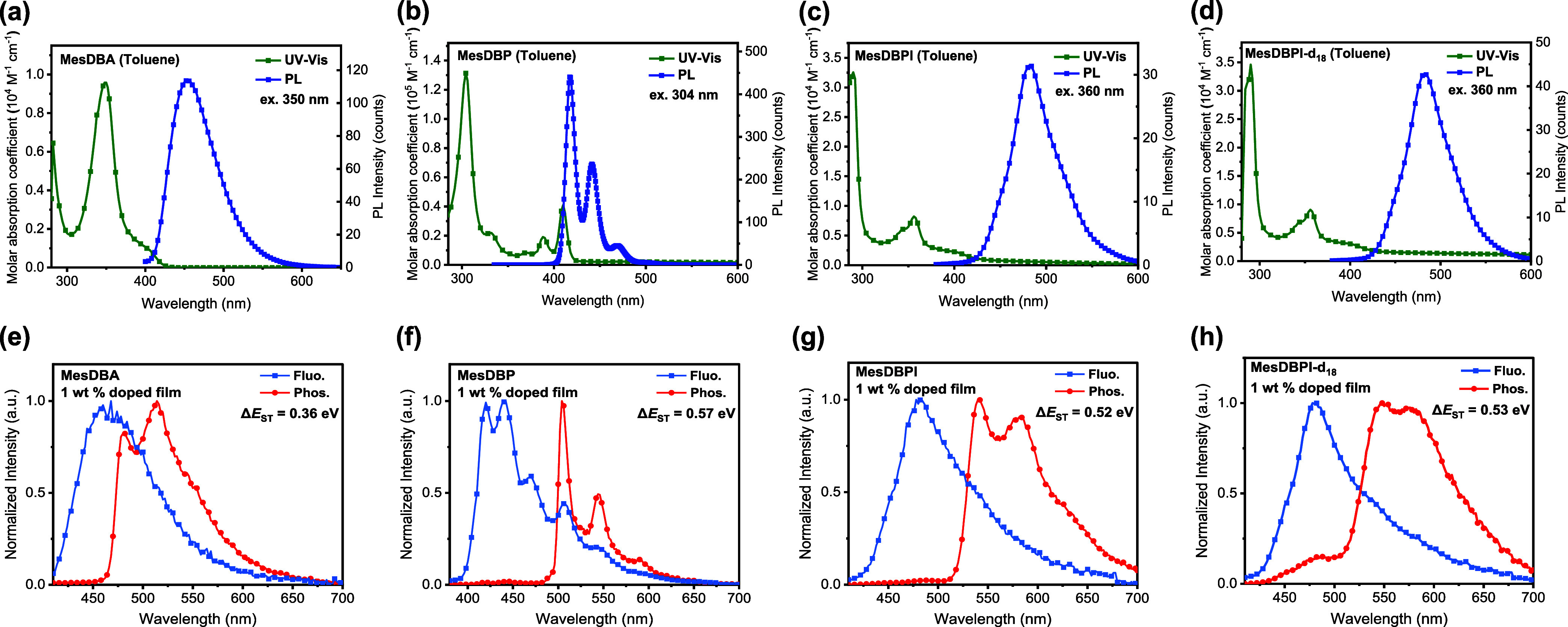
UV/vis absorption and fluorescence spectra of (a) MesDBA, (b) MesDBP, (c) MesDBPI, and (d) MesDBPI-*d*_18_ in toluene. Fluorescence (300 K) and phosphorescence (100 K) spectra of (e) MesDBA, (f) MesDBP, (g) MesDBPI, and (h) MesDBPI-*d*_18_ in 1 wt % doped PMMA films.

**Figure 3:**
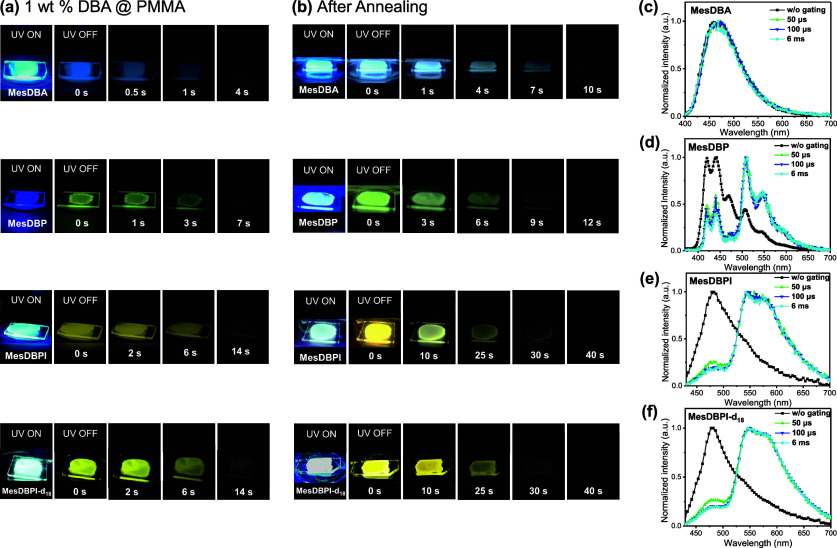
Photographs of (a) 1 wt % DBA doped PMMA and (b) annealed films with and without UV illumination. Time-gated PL spectra of (c) MesDBA, (d) MesDBP, (e) MesDBPI, and (f) MesDBPI-*d*_18_ in 1 wt % doped PMMA films.

### Afterglow Mechanism

To further elucidate the origin of the afterglow in the four molecules, temperature-dependent PL measurements, transient PL spectroscopy, transient absorption spectroscopy, and theoretical calculations were conducted. At first, temperature-dependent PL spectra of all emitters were measured in 1 wt % doped PMMA films from 100 to 300 K after a 6 ms delay (Figure 4a–d). The time-gated emission spectra of four emitters at 300 K are composed of higher-energy band peaks at 460 nm for MesDBA, 440 nm for MesDBP, and 482 nm for MesDBPI/MesDBPI-*d*_18_, respectively, and are nearly identical to prompt fluorescence spectra. The intensities of these high-energy bands increased from 100 to 300 K, which may originate from TADF, likely facilitated by thermal activation at 300 K. Moreover, the lower-energy bands observed at around 500–700 nm for MesDBP, MesDBPI, and MesDBPI-*d*_18_ are consistent with their previously assigned phosphorescent components. All compounds exhibit similar afterglow emission patterns (Figure S22) in the PMMA matrix across different concentrations (0.1, 1, and 10 wt %), thereby ruling out TTA as the origin of delayed fluorescence.

**Figure 4:**
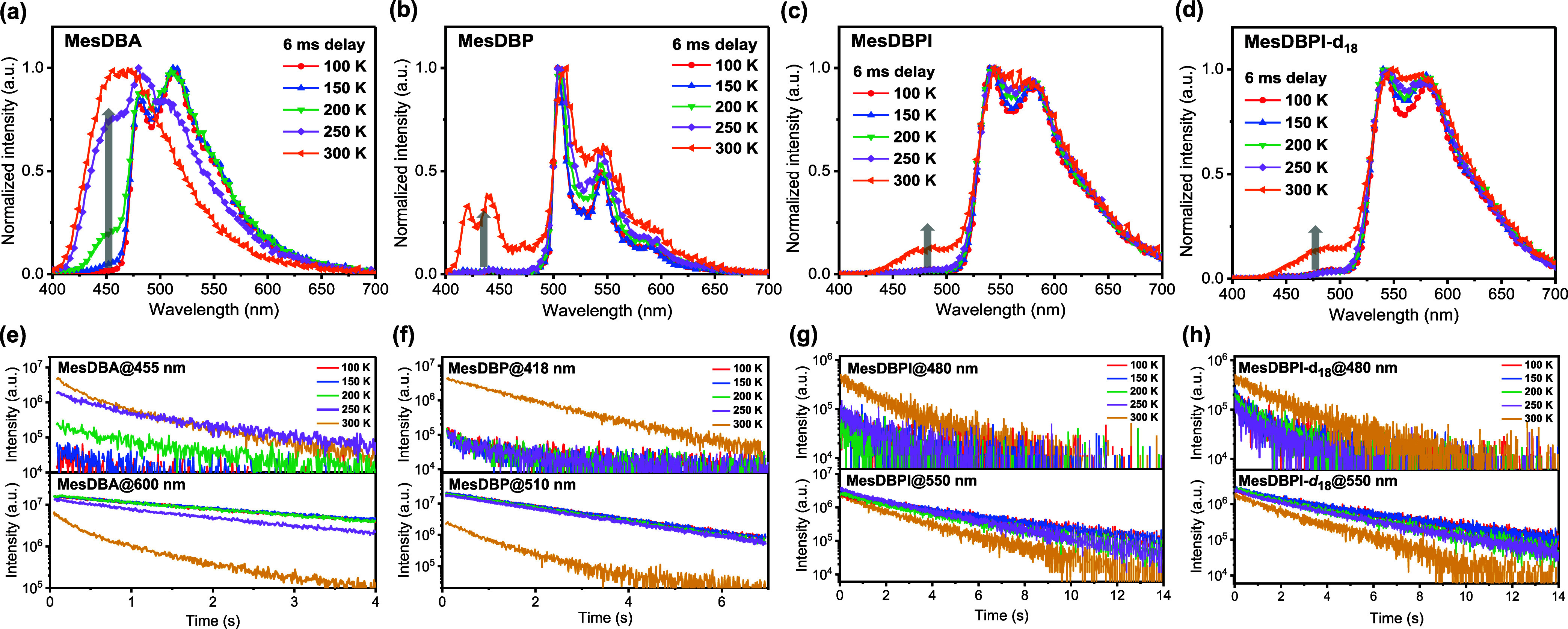
Temperature-dependent PL spectra obtained at 6 ms delay of (a) MesDBA, (b) MesDBP, (c) MesDBPI, and (d) MesDBPI-*d*_18_ in 1 wt % doped PMMA films. Temperature-dependent PL decay curves of (e) MesDBA, (f) MesDBP, (g) MesDBPI, and (h) MesDBPI-*d*_18_ in 1 wt % doped PMMA films, respectively.

To validate the hypothesis, we measured the transient PL spectra of doped PMMA films over the temperature range of 100–300 K (Figure 4e–h). Prompt decays were recorded within a 100 ns window (Figure S23), whereas the long-lived components were monitored up to the second time scale (Figure S24). The prompt fluorescence (τ_PF_) and delayed fluorescence (τ_DF_) lifetimes of MesDBA are fitted as 4.7 ns and 0.58 s, respectively. MesDBP and MesDBPI display an additional phosphorescence component with lifetimes of 4.5 ns (τ_PF_), 1.26 s (τ_DF_), and 1.10 s (τ_Ph_) for MesDBP and 41.8 ns, 2.02 s, and 2.37 s for MesDBPI, respectively. Moreover, MesDBPI-*d*_18_ exhibited the longest lifetimes (52.2 ns, 2.54 s, and 2.80 s) among all compounds. The delayed fluorescence intensities increase with a rising temperature for MesDBA (455 nm), MesDBP (418 nm), and MesDBPI/MesDBPI-*d*_18_ (480 nm), confirming their TADF characteristics. In contrast, the red-shifted bands of MesDBP (510 nm) and MesDBPI/MesDBPI-*d*_18_ (550 nm) exhibit an opposite temperature dependence with increasing intensity upon cooling, which is characteristic of phosphorescence. The PLQYs of MesDBA, MesDBP, MesDBPI, and MesDBPI-*d*_18_ in 1 wt % doped PMMA films at room temperature are 20.1%, 56.1%, 30.7%, and 31.5%, respectively. The afterglow quantum yield (Φ_A_) of MesDBP, MesDBPI, and MesDBPI-*d*_18_ is a sum of persistent TADF and RTP components (Φ_A_ = Φ_DF_ + Φ_Ph_). According to the afterglow spectral composition, only TADF contributes to overall afterglow efficiency in the case of MesDBA (Φ_DF_ = 19.6%), while in terms of MesDBP, it is the result of both TADF (Φ_DF_ = 1.7%) and RTP (Φ_Ph_ = 7.5%). Similarly, MesDBPI also features combined pathways with Φ_DF_ (0.9%) and Φ_Ph_ (12.0%). For MesDBPI-*d*_18_, the corresponding values are 1.2% and 11.9%, respectively. Notably, the introduction of iptycene units into afterglow emitters, as demonstrated by MesDBPI and MesDBPI-*d*_18_, yields simultaneous enhancements in Φ_A_ and afterglow lifetimes compared to those of MesDBP, marking the iptycene extension as a distinctive structural strategy for long-lived emitter design. Transient PL measurements of 0.1 and 10 wt % doped PMMA films (Figures S23 and S24) consistently exhibit similar PL profiles and afterglow characteristics, confirming that the emission arises from intrinsic molecular properties rather than intermolecular mechanisms such as aggregation or TTA. The corresponding photophysical parameters are summarized in Table S3. The combined analysis of emission lifetimes and PLQYs confirms that all emitters possess intrinsically slow RISC processes with *k*_RISC_ of 6.79 × 10^1^ s^–1^ for MesDBA, 5.40 × 10^–2^ s^–1^ for MesDBP, 3.00 × 10^–2^ s^–1^ for MesDBPI, and 3.10 × 10^–2^ s^–1^ for MesDBPI-*d*_18_. The rate constants for films at various concentrations are summarized in Table S4. Following thermal annealing, τ_PF_ remains nearly unchanged across all compounds, while τ_DF_ and τ_Ph_ are significantly prolonged (Figure S25), consistent with the increased durations observed in the imaging results. The PLQY of MesDBA increases substantially from 20.1% to 56.4%, indicating enhanced radiative efficiency. The transition in afterglow bands before and after annealing reflects an altered TADF/RTP balance. Thermal annealing can enhance RTP by optimizing molecular packing and restricting molecular motion.^82^ For both MesDBPI and MesDBPI-*d*_18_, the color shift from green to yellowish (Figures 3 and S21) and the reduced *k*_RISC_ (Table S4) indicate suppressed RISC and enhanced RTP.^37^ A summary of the representative photophysical data is provided in Table 1. Among reported TADF molecules, MesDBA establishes the current record for the longest delayed lifetime (0.72 s), realizing afterglow in the absence of lone pair-containing groups, crystallization, heavy atoms, molecular aggregation, or host–guest interactions.^83^ Furthermore, the iptycene unit was introduced for the first time, yielding a favorable balance between efficient ISC and slow RISC in MesDBPI and affording a hybrid afterglow with two lifetimes of 3.67 and 3.70 s. Ultimately, deuterated methyl groups in MesDBPI-*d*_18_ further extended their lifetimes to 4.00 s (TADF) and 4.22 s (RTP), which represent the longest values reported among organic TADF and organoboron RTP emitters. Although not surpassing the absolute RTP benchmark of coronene derivatives (∼6 s in PMMA),^81,84^ these results remain highly competitive for single-dopant systems in inert polymers and highlight the strong potential of our design strategy. Representative long-lived emitters with second-scale lifetimes are summarized in Table S5 for comparison.^3,85,86^ The afterglow performance of the DBA series could be further enhanced through strategies such as full deuteration and matrix or host engineering, which may suppress nonradiative decay pathways and extend the lifetimes.

**Table 1:** Summary of the Photophysical Properties of MesDBPI, MesDBA, MesDBP, and MesDBP-*d*_18_ Doped in 1 wt % PMMA at Room Temperature

Emitter	λ_PL_ ^a^ [nm]	Δ*E*_ST_ ^b^ [eV]	Φ_PL_ ^c^ [%]	Φ_PF_ ^d^ [%]	Φ_DF_ ^d^ [%]	Φ_Ph_ ^d^ [%]	τ_PF_ ^e^ [ns]	τ_DF_ ^e^ [s]	τ_Ph_ ^e^ [s]	*k*_RISC,exp_ ^f^ (s^–1^)	*k*_RISC,cal_ ^g^ (s^–1^)
MesDBA	460	0.36	20.1	0.5	19.6		4.7	0.58		6.79 × 10^1^	1.06 × 10^1^
MesDBA^h^			56.4	1.6	54.8		5.0	0.72		4.83 × 10^1^	1.21 × 10^1^
MesDBP	440	0.57	56.1	46.9	1.7	7.5	4.5	1.26	1.10	5.40 × 10^–2^	9.65 × 10^–1^
MesDBP^h^			45.3	39.3	0.8	5.2	4.6	1.42	1.41	2.40 × 10^–2^	7.32 × 10^–2^
MesDBPI	482	0.52	30.7	17.8	0.9	12.0	41.8	2.02	2.37	3.00 × 10^–2^	1.10 × 10^–2^
MesDBPI^h^			25.5	16.0	0.8	8.7	46.5	3.67	3.70	1.60 × 10^–2^	3.73 × 10^–3^
MesDBPI-*d*_18_	482	0.53	31.5	18.4	1.2	11.9	52.2	2.54	2.80	3.10 × 10^–2^	9.63 × 10^–3^
MesDBPI-*d*_18_ ^h^			27.7	17.0	0.7	10.0	59.2	4.00	4.22	1.20 × 10^–2^	2.94 × 10^–3^

a PL peak.

b Singlet–triplet gap.

c Absolute PLQY (Φ_PL_).

d The prompt fluorescent (Φ_PF_), delayed fluorescent (Φ_DF_), and phosphorescent (Φ_Ph_) component of PLQY.

e Lifetime of the prompt fluorescence (τ_PF_), delayed fluorescence (τ_DF_), and phosphoresce (τ_Ph_) determined from the transient PL.

f Rate constant of RISC determined from experimental data.

g Rate constant of RISC simulated by eq 2.

h After annealing process.

To further probe the higher-lying triplet excited states (T_*n*_) of MesDBPI, near-infrared transient absorption spectroscopy (NIR-TAS) was conducted in THF solutions (0.50, 0.75, and 1.00 mM). As shown in Figure 5a, two distinct near-infrared (NIR) absorption bands are observed in the 5000 to 8500 cm^–1^ region, likely corresponding to higher triplet–triplet absorption transitions. For quantitative evaluation, the NIR region was segmented into two characteristic spectral ranges, 6000 to 6500 cm^–1^ (B1) and 6800 to 7300 cm^–1^ (B2), based on the absorption pattern. Subsequent integration and time-resolved analyses were performed for each region to investigate their respective decay profiles. To validate the kinetic origins of both bands, each region was integrated and subjected to analysis of their time-resolved decay profiles. Figure 5b exhibits identical decay rates for each band, confirming that they originate from the same T_1_ population, as supported by the theoretical simulations discussed later. For the 0.50 mM THF solution, the decay profile of the B1 band was well fitted with a biexponential function, yielding lifetimes of 24.7 and 174.0 μs. As summarized in Table S6, the slower decay component displays a concentration-dependent reduction in lifetime as the MesDBPI concentration increases from 0.50 to 1.00 mM, suggesting self-quenching effects arising from intermolecular interactions (Figure 5c). Additionally, efforts were made to detect afterglow from MesDBPI in THF solution at room temperature. However, no delayed emission was observed under these conditions. This is likely due to collisional quenching of the triplet state population by the solute molecules themselves in the solution phase. Importantly, the decay curves of the long-lived triplet state exhibit biexponential behavior, a phenomenon rarely observed in organic emitters, possibly due to contributions from triplet sublevels.^87–89^ These findings reinforce the significance of molecular modulation for triplet state levels in achieving organic afterglow.

**Figure 5:**
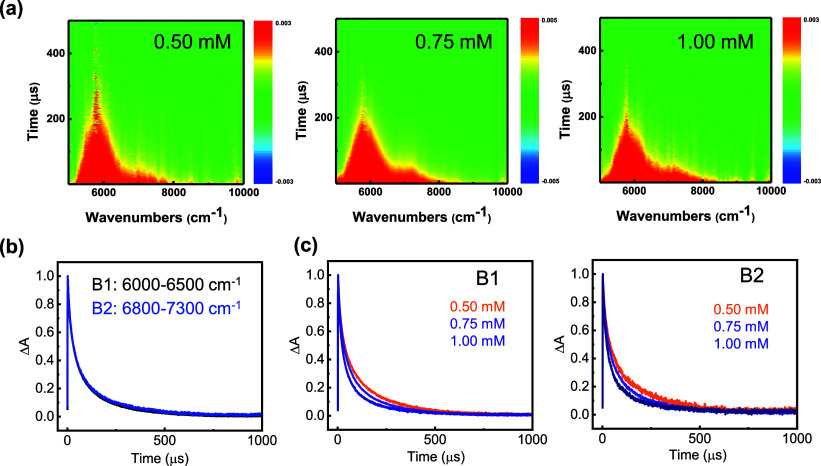
Near-infrared transient absorption spectroscopy (NIR-TAS) of MesDBPI in tetrahydrofuran (THF) solutions. (a) Contour maps of the transient absorption at various concentrations. (b) Normalized decay profiles of differential absorbance integrated over distinct spectral ranges: black, 6000–6500 cm^–1^; blue, 6800–7300 cm^–1^. (c) Normalized decay profiles of the differential absorbance for samples at various concentrations.

Density functional theory (DFT) and time-dependent DFT (TD-DFT) (Figure S26, Tables S7 and S8) were performed to investigate frontier molecular orbitals (FMOs) and electronic excited states for all DBA derivatives. As shown in Figure S27, the HOMO of MesDBA is mainly located on the mesityl (Mes) unit, and the LUMO is located primarily on DBA units. In contrast, the HOMOs of MesDBP, MesDBPI, and MesDBPI-*d*_18_ are mainly located on conjugation DBP and DBPI, respectively, and the LUMO distribution is similar to that of MesDBA. These alternative FMOs indicate strong CT, strong LE, and weak CT characteristics for MesDBA, MesDBP, and MesDBPI, respectively. MesDBPI and MesDBPI-*d*_18_ show narrower HOMO–LUMO gaps of 4.24 eV than MesDBA (4.37 eV) and MesDBP (4.72 eV), indicating that red-shift emission can be attained for MesDBPI. The natural transition orbitals (NTOs) of the singlet and triplet excited states were also simulated, as illustrated in Figure 6. For MesDBA, the S_1_ state possesses a CT character with the transition from Mes to DBA. The S_1_ state of MesDBP shows a similar CT character with MesDBA. The T_2_ and T_3_ states of MesDBA comprise the similar “hole” and “electron” distributions as S_1_, whereas T_1_ possesses a LE nature. For MesDBP, T_*n*_ (*n* = 1–3) states all possess LE characters. However, the S_1_ state of MesDBPI and MesDBPI-*d*_18_ exhibit mixed CT/LE character, mainly localized on the DBPI unit due to its extended conjugation. The T_1_ and T_3_ states of MesDBPI and MesDBPI-*d*_18_ comprise similar “hole” and “electron” distributions as S_1_, whereas T_2_ possesses a hybridized local and charge transfer (HLCT) nature. Moreover, transitions involving T_*n*_ (*n* = 1–5) states of MesDBPI, calculated at the optimized T_1_ geometry, indicate that the B1 and B2 bands observed in the NIR-TAS spectra correspond to the T_1_ → T_3_ and T_1_ → T_4_ transitions with calculated oscillator strengths of 0.27615 and 0.08466, respectively (Figure S28). This result further supports the assignment of the absorptions to the T_1_-origin proposed in Figure 5b.

**Figure 6:**
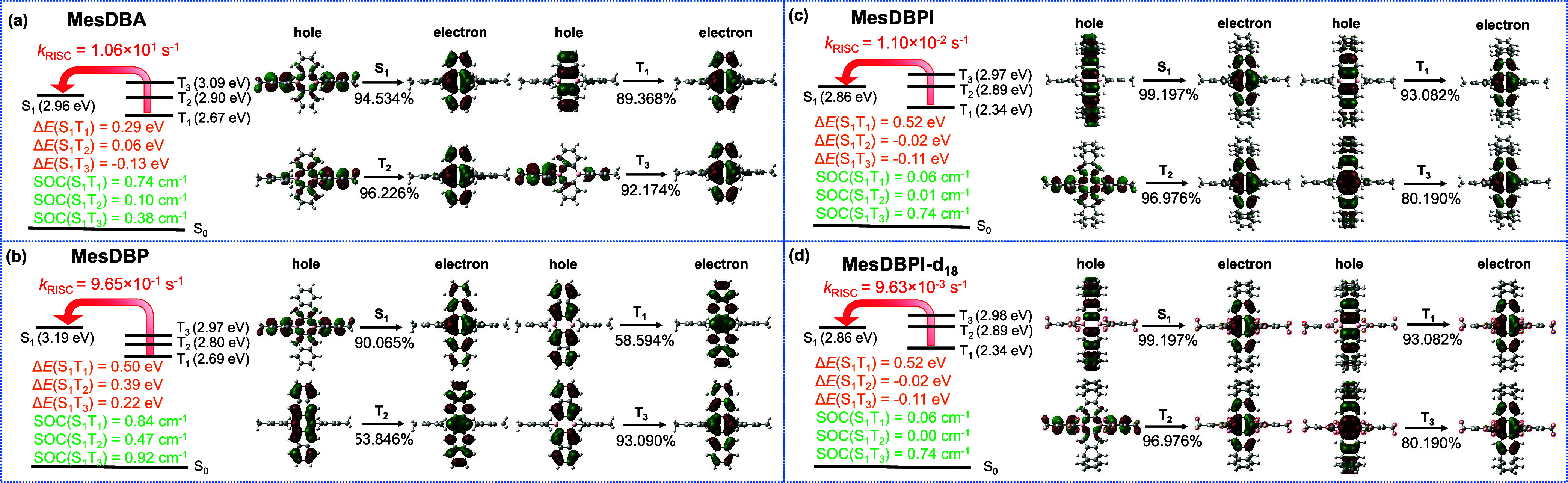
Excited state energy levels, spin–orbit coupling constants, and natural transition orbital analysis of S_1_, T_1_, T_2_, and T_3_ states calculated for (a) MesDBA, (b) MesDBP, (c) MesDBPI, and (d) MesDBPI-*d*_18_ at the BMK/6-31G­(d) level in the solid phase.

The singlet–triplet energy gaps and spin–orbit coupling (SOC) constants between S_1_ and T_*n*_ (*n* = 1–3) play a crucial role in the up-conversion process. For MesDBA, the largest SOC among T_*n*_ (*n* = 1–3) is SOC­(S_1_T_1_), obtained as 0.74 cm^–1^. For MesDBPI and MesDBPI-*d*_18_, the S_1_-T_3_ SOC constant (SOC­(S_1_T_3_)) of 0.74 cm^–1^ is much larger than those of SOC­(S_1_T_1_) and SOC­(S_1_T_2_), consistent with the mixed CT/LE character of T_3_. In contrast, for MesDBP, SOC values between S_1_ and T_*n*_ (*n* = 1–3) are all large, about 0.47–0.92 cm^–1^, due to the LE nature of T_*n*_ (*n* = 1–3). Compared to MesDBA and MesDBP, the triptycene units determine more extended conjugation in the polycyclic scaffolds of MesDBPI and MesDBPI-*d*_18_. Thus, the extended conjugation structures of MesDBPI and MesDBPI-*d*_18_ result in a lower energy of 2.86 eV for S_1_, and Δ*E*(S_1_T_1_) values of MesDBA, MesDBP, MesDBPI, and MesDBPI-*d*_18_ are 0.29, 0.50, 0.52, and 0.52 eV in the solid phase at BMK/6-31G­(d), respectively (Table S7). In Figure S29a, the charge distributions of the HOMO and LUMO in MesAn are both localized on the anthracene (An) unit, indicating a pure LE transition. With boron introduction, the empty p orbitals in MesDBA redistribute the FMOs, producing a clear CT character from the mesityl groups to DBA and reducing Δ*E*(S_1_T_1_) from 1.26 eV (MesAn) to 0.29 eV (MesDBA). The corresponding excited-state analysis of MesAn (Figure S29b) further supports this conclusion: the calculated small SOC constants and LE-dominated T_1_–T_3_ states indicate that both ISC and RISC processes are inefficient. Overall, these results confirm that targeted boron substitution is the key design element for transforming a conventional fluorescent framework into a TADF-active system. Subsequently, larger Δ*E*(S_1_T_1_) and small SOC­(S_1_T_1_) of MesDBP, MesDBPI, and MesDBPI-*d*_18_ may produce triplet excitons for RTP. Moreover, the T_2_ or T_3_ states of MesDBP, MesDBPI, and MesDBPI-*d*_18_ exhibit narrow energy gaps with the S_1_ state (Table S8) and possess strong SOC, enabling possible RISC channels from the higher triplet states to S_1_. The decreasing dihedral angles from MesDBA to MesDBPI-*d*_18_ suggest enhanced planarity and orbital overlap (Figure 1), which may promote SOC and facilitate RISC. It is worth noting that dynamic torsional motions can transiently boost SOC;^90^ however, our solid-state calculations limit conformational sampling, and the SOC contributions vary depending on the specific triplet states involved. In addition, MesDBA exhibits a higher ISC quantum yield and afterglow intensity due to the narrow energy gap between S_1_ and T_*n*_ states and strong SOC consistent with El-Sayed’s rule. In contrast, MesDBPI and MesDBPI-*d*_18_ show slower ISC and RISC rates, which contribute to the longer afterglow duration. According to Fermi’s golden rule and Marcus theory, the RISC rate can be simulated as in the follow equation:^91,92^

1 $$k_{\mathrm{RISC}}=\frac{2\pi }{\hbar }{\left|H_{\mathrm{SOC}}\right|}^{2}\sqrt{\frac{1}{4\pi \lambda_{\mathrm{RISC}}k_{B}T}}\;\exp\left[-\frac{{\Delta E}_{a}^{\mathrm{RISC}}}{k_{B}T}\right]$$

Here, *H*_SOC_ is the SOC between S_1_ and T_*n*_ (*n* = 1–3), *k*_B_ is the Boltzmann constant, Δ*E*_a_^RISC^ is defined as activation energy, ${\Delta E}_{a}^{\mathrm{RISC}}=\frac{{\left({\Delta E}_{\mathrm{ST}}+\lambda_{\mathrm{RISC}}\right)}^{2}}{4\lambda_{\mathrm{RISC}}}$ , *T* is the temperature, and λ_RISC_ is the reorganization energy, λ_RISC_ = *E*(S_1_@T_*n*_) – *E*(S_1_). Here, *E*(S_1_@T_*n*_) is the energy of the S_1_ at the geometry of T_*n*_ (*n* = 1–3).^93^ Based on the singlet–triplet gaps and SOC analyses, high-lying triplet states like T_2_ and T_3_ may participate in the RISC process. Thus, the effective RISC rate (*k*_RISC_^eff^) can be defined as^94^

2 $$k_{\mathrm{RISC}}^{\mathrm{eff}}=\frac{P_{1}{k_{{T_{1}-S}_{1}}}^{2}+P_{2}{k_{{T_{2}-S}_{1}}}^{2}+P_{3}{k_{{T_{3}-S}_{1}}}^{2}}{k_{{T_{1}-S}_{1}}+k_{{T_{2}-S}_{1}}+k_{{T_{3}-S}_{1}}}$$

In eq 2, *P*_1_, *P*_2_, and *P*_3_ represent the Boltzmann distribution ratios of T_*n*_ (*n* = 1–3), respectively. The ratio of T_*n*_ (*n* = 1–3) is calculated using the Boltzmann distribution, $P_{i}=e^{-{\Delta E}_{i}/RT}/\underset{i}{\sum }e^{-{\Delta E}_{i}/RT}$ . As listed in Table 1, *k*_RISC,cal_ values of MesDBA, MesDBP, MesDBPI, and MesDBPI-*d*_18_ are calculated to be 1.06 × 10^1^, 9.65 × 10^–1^, 1.10 × 10^–2^, and 9.63 × 10^–3^ s^–1^, which agree well with experimental data. The decrease in *k*_RISC,cal_ values after the annealing simulation indicates that the RISC processes are further suppressed. For comparison, MesAn exhibits no TADF owing to its extremely low *k*_RISC_ (2.79 × 10^–20^ s^–1^), as shown in Figure S29. These findings highlight that higher-lying triplet states with strong SOC are key determinants of the RISC rate (Table S9) and play a pivotal role in the manifestation of TADF. Though MesDBP and MesDBPI exhibit large Δ*E*_ST_ values, the presence of higher-lying triplet states with strong SOC facilitates RISC, thus enabling TADF. Moreover, high steric hindrance and rigid conformation of MesDBP and MesDBPI suppress intermolecular quenching and the nonradiative triplet decay, thereby enabling RTP. These results show that MesDBA can exhibit pure TADF-type afterglow, while MesDBP, MesDBPI, and MesDBPI-*d*_18_ emit TADF-RTP hybrid afterglow.^16,37^

The radiative rate constant of T_1_ → S_0_ (*k*_r,T_) and its radiative lifetime (τ_r,T_) can be determined by eq 3.^10,95^

3 $$k_{r,T}=\frac{1}{\tau_{r,T}}=\frac{{{\Delta E}_{T_{1}}}^{3}{\left|\mu_{T_{1}}\right|}^{2}}{3\pi \varepsilon_{0}\hbar^{4}c^{3}}$$

where Δ*E*_T_1__ and μ_T_1__ are the energy difference and transition dipole moment between T_1_ and S_0_ states, respectively, ε_0_ is the vacuum permittivity, *c* is the velocity of light, and ℏ is the reduced Planck constant. Compared to MesDBP, triptycene units in MesDBPI extend conjugation, lowering the T_1_ energy to 2.34 eV. Moreover, μ_T_1__ of MesDBPI (7.75 × 10^–5^ Debye) is lower than that of MesDBP (1.65 × 10^–4^ Debye). This difference is attributed to the steric hindrance introduced by the triptycene units, which reduces the level of electron localization along the molecular backbone. According to eq 3, τ_r,T_ is inversely proportional to Δ*E*_T_1__ and μ_T_1__. MesDBP and MesDBPIs' τ_r,T_ values are approximately 14 and 106 s, respectively. Based on the annealing simulation, the τ_r,T_ values of MesDBP and MesDBPI are approximately 17 and 581 s, respectively, indicating significantly prolonged phosphorescence lifetimes after the annealing process. Moreover, suppression of the nonradiative triplet decay process in MesDBPI-*d*_18_ results in a longer phosphorescence lifetime than that of MesDBPI, highlighting the isotope effect.^10^ The trends align well with experimental observations, indicating that the incorporation of conjugated steric hindrance into the diboron framework effectively prolongs the phosphorescence lifetime. This finding provides valuable guidance for the rational design of molecules with extended afterglow lifetimes.

### Afterglow Applications

To assess the EL performance of MesDBA and MesDBPI, we fabricated two OLED devices employing the architecture shown in Figure 7a. The emitting layer (EML, 20 nm) comprises MesDBA or MesDBPI, each doped into the mCPCN host. mCPCN was chosen for its bipolar characteristics and high triplet energy (*E*_T_ = 3.03 eV),^96^ which effectively improves the charge balance and minimizes reverse energy transfer. Full fabrication procedures and molecular structures (Figure S30) are provided in the Supporting Information. Device characterization results are presented in Figure 7b,c and Figure S31, and the performance metrics of the afterglow OLEDs are summarized in Table 2. The optimized MesDBA-based device exhibits a low driving voltage of 3.0 V and an EQE of 8.0%, which is relatively high compared to those of reported afterglow OLEDs (Table S10), while the MesDBPI-based device shows a lower EQE of 1.8%. The delayed time-resolved EL spectra show that, for MesDBA, the delay emission matches the steady-state spectra, indicating TADF-dominated delayed EL. For MesDBPI, a gradual increase of the long-lived component at longer delays suggests a more pronounced RTP contribution compared to that of TADF (Figure 7d,e). In addition, the delayed EL spectra (Figure 7e) exhibit a larger contribution from the TADF region relative to PL, implying that, under electrical excitation, the emission balance could be affected by additional pathways. Among the possible mechanisms, TTA cannot be entirely excluded. Time-resolved EL measurements reveal prolonged emission for both devices with afterglow durations of approximately 0.5 and 1.0 s, respectively. Notably, the MesDBPI-based device maintains a pronounced electrically driven EL with a lifetime of 113 ms, significantly exceeding the 11 ms observed for MesDBA (Figure 7f). Moreover, most reported afterglow devices emit in the green-yellow region, while our work focuses on afterglow OLEDs incorporating both TADF- and RTP-type emitters, and the present devices extend the emission toward the blue region. In addition, the devices exhibit a lower driving voltage, which enhances their practical applicability. To better understand the performance differences, we further investigated the photophysical properties of MesDBA and MesDBPI in the mCPCN host. The relatively high dielectric constant of mCPCN (3.1)^97^ provides a more polar environment, which stabilizes the CT excited state of TADF emitters. This stabilization causes a red-shift in fluorescence emission and reduces Δ*E*_ST_. As a result, the afterglow behavior of MesDBA was not observed in earlier studies because polar solid hosts and solution conditions inhibit its long-lived emission. The higher EQE of the MesDBA-based device can be attributed to its higher PLQY of 32.3% and a narrower Δ*E*_ST_ of 0.18 eV, compared to the lower PLQY of 17.5% for MesDBPI under identical conditions (Figure S32). However, this enhanced upconversion rate in MesDBA also results in the complete shortening of the afterglow emission in the solid state. In contrast, MesDBPI preserves hybrid afterglow characteristics in the same host, though with a reduced emission duration of approximately 6 s. Time-resolved measurements further reveal distinct TADF and RTP lifetimes of 1.09 and 1.44 s, respectively (Figure S33). The corresponding EL afterglow lifetime, however, is limited to 113 ms in the same mCPCN host. Analogous PL-EL asymmetry in afterglow behavior has also been reported, where EL durations on the order of seconds were observed only in devices with modest EQE,^22,24,25^ whereas higher-efficiency devices often exhibited reduced EL lifetime.^26^ These results indicate a trade-off between RISC efficiency and persistent luminescence influenced by fine-tuned host–guest interactions and excited-state dynamics. Future progress toward combining high efficiency with ultralong EL will likely rely on strategies such as controlling dopant concentration, engineering host materials, and optimizing device architecture.

**Table 2:** Summary of Electroluminescent Properties of Afterglow OLEDs

Device^a^	*V*_d_ ^b^ [V]	*L*_max_ ^c^ (cd m^-2^, V)	η_ext_ ^d^ [%]	η_c_ ^e^ [cd A^-1^]	η_p_ ^f^ [lm W^-1^]	λ_max_ ^g^ [nm]	CIE_(*x*,*y*)_ ^h^	EL Lifetime [ms]
MesDBA	3.0	464, 12.5	8.0	19.4	17.4	491	(0.19, 0.37)	11.0
MesDBPI	3.5	461, 16.0	1.8	3.9	3.5	488	(0.16, 0.36)	113.0

a ITO/MoO_3_ (1 nm)/TAPC (50 nm)/mCP (10 nm)/mCPCN:10 wt % MesDBA or MesDBPI (20 nm)/3TPYMB (55 nm)/LiF (1 nm)/Al (100 nm).

b *V*_d_, the driving voltage at the brightness of 1 cd m^-2^.

c *L*_max_, the maximum luminance.

d η_ext_, the maximum external quantum efficiency.

e η_c_, the maximum current efficiency.

f η_p_, the maximum power efficiency.

g λ_max_, the wavelength of the EL spectrum with maximum intensity at 8 V.

h CIE_(*x*,*y*)_, the CIE coordinate of the EL spectrum at 8 V.

**Figure 7:**
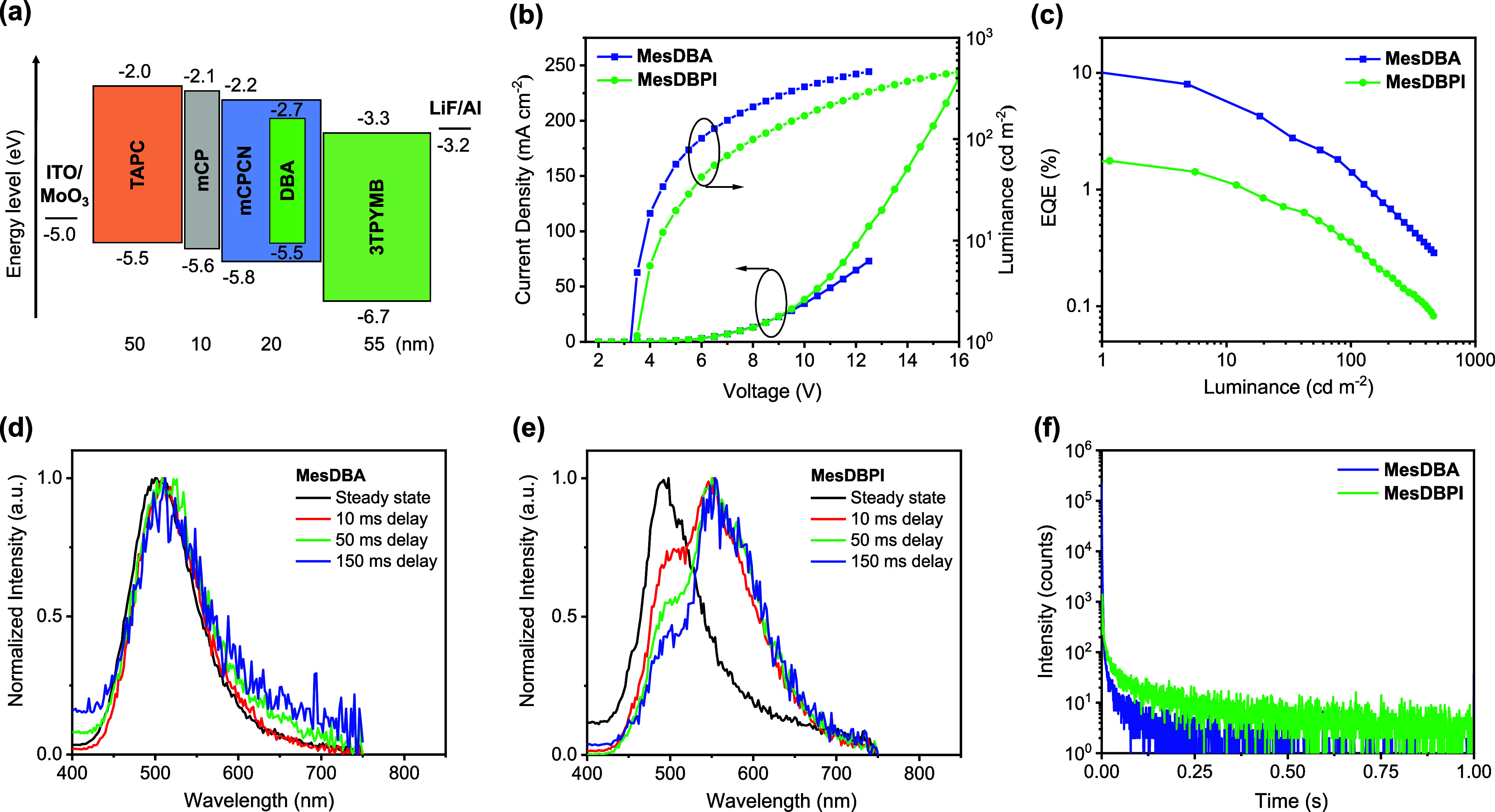
(a) Device configuration and energy level diagram of the materials employed in the devices. (b) Current density–voltage–luminance plot. (c) External quantum efficiency vs luminance plot. EL spectra of 10 wt % (d) MesDBA and (e) MesDBPI, recorded under steady-state conditions and at delayed times of 10, 50, and 150 ms, each with a 50 ms gate width. (f) The decay curves of transient EL spectra. The applied voltage is 5.0 V, while the frequency is 1 kHz.

To demonstrate the practical utility of afterglow DBA materials, their distinct emission lifetimes and colors were applied in dynamic anticounterfeiting and information encryption scenarios (Figure 8). PMMA-based inks incorporated with MesDBA, MesDBP, and MesDBPI were used to create graphical patterns with time-dependent emissive behavior. Additionally, 9,10-diphenylanthracene (DPA), a blue-emissive molecule without an afterglow, was incorporated as a fluorescent reference emitter to enhance the contrast between short-lived and persistent signals. In the first demonstration (Figure 8a), the letters “NTHU” were patterned on a zinc alloy engraved substrate using PMMA inks containing MesDBA (unannealed), MesDBP (annealed), and MesDBPI (annealed), respectively. Under a nitrogen atmosphere, Movie S1 captures a multicolor emission sequence upon removal of the UV excitation source: a brief blue “N” (∼4 s from MesDBA), a green “U” (∼8 s from MesDBP), and a long-lasting yellow “TH” (∼30 s from MesDBPI). This selective thermal treatment strategy enables precise modulation of emission lifetimes, allowing temporal encoding of multilevel visual information for anticounterfeiting purposes. In the second demonstration (Figure 8b), the initial numeric pattern “8888” was coated on an acrylic substrate using DPA and afterglow inks and was clearly visible under UV illumination. Upon UV removal, the blue-emitting DPA rapidly faded, transforming the pattern into “2025” as the persistent emission from the DBA derivatives emerged. Continued decay of the afterglow components ultimately revealed “25”, demonstrating a programmable, stepwise information release enabled by rational utilization of afterglow emitters with distinct durations. It is worth noting that the afterglow durations of three inks were somewhat reduced on alternative substrates compared to those on quartz plates, potentially due to differences in substrate surface properties or thermal characteristics. These observations highlight the importance of substrate selection in achieving optimal afterglow performance. Together, the demonstrations highlight the potential of single-molecule DBA emitters for next-generation time-gated photonic encryption. By linking precise molecular design with device-level functionality, this work establishes a universal strategy for controlling afterglow behavior across both optical and electroluminescent platforms, paving the way for advanced optoelectronic and security applications.

**Figure 8:**
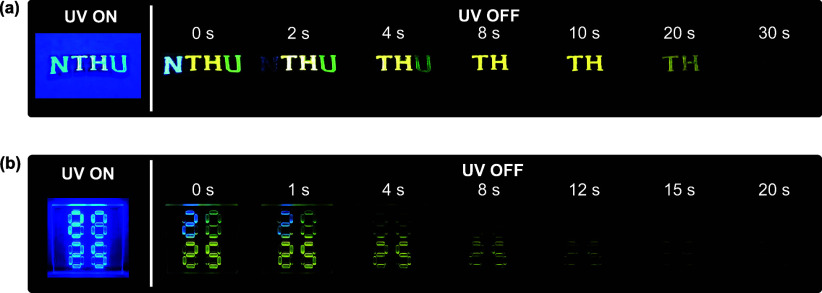
(a) Visual demonstration of a multilevel anticounterfeiting application featuring the NTHU letters, recorded under 365 nm UV excitation and in the absence of illumination. (b) Photographic sequence of an encryption demonstration, in which the initial pattern “8888” is converted to “2025” upon turning off the 365 nm UV light, followed by gradual fading to “25” over time.

## Conclusion

In summary, diboron-based systems exhibit distinct afterglow profiles at room temperature, demonstrating the versatility of DBA scaffolds in modulating singlet–triplet excited-state transitions. Both theoretical calculations and experimental measurements demonstrate that precise tuning of the singlet–triplet splitting and RISC rate enables systematic control over key afterglow characteristics, including emission type (TADF or hybrid dominant), luminescence color, and persistence. Nearly three decades after its first report, MesDBA has been redefined from a conventional fluorophore into the first pure TADF emitter of the DBA family, exhibiting the longest emission lifetime (0.72 s) among organic TADF emitters, together with a 10 s afterglow duration. This unprecedented performance originates from the synergistic interplay of molecular and environmental factors: boron substitution and mesityl donor generate a moderate Δ*E*_ST_ that enables slow yet effective RISC, while the rigid and low-polar PMMA matrix minimizes nonradiative decay to sustain ultralong TADF. Subsequently, π-extension in MesDBP drives its evolution by introducing an additional RTP component and extending the duration to 12 s, highlighting the critical role of structural modifications in stabilizing triplet excitons. Further structural refinement via iptycene incorporation yields MesDBPI, which achieves a hybrid TADF-RTP afterglow in PMMA. The emission shows a duration of up to 40 s and a lifetime of 3.70 s, marking a benchmark among boron-induced afterglow systems. Notably, the site-selectively deuterated MesDBPI-*d*_18_ further extends this record to 4.00 s (TADF) and 4.22 s (RTP), highlighting the effectiveness of steric and isotopic engineering in modulating singlet–triplet radiative pathways. These results also unveil the critical role of the diboron framework in enabling efficient ISC and triplet-state stabilization without the need for heavy atoms or matrix-induced effects. Complementing these experimental advances, we also establish a predictive theoretical model based on Marcus theory that accurately estimates their RISC rate constants and aligns well with measured values across all systems. The outstanding afterglow properties of these air-stable DBA molecules further translate into optical and electronic applications with the OLEDs based on MesDBA and MesDBPI achieving EQEs of 8.0% and 1.8%, respectively. This study bridges TADF and RTP through rational scaffold engineering and establishes a unified molecular design strategy linking the architecture, excited-state dynamics, and device performance. Insights from comparisons between boron-substituted and boron-free frameworks further indicate that this concept can extend to a broader class of boron-doped π-conjugated systems, opening new avenues for organic afterglow emitters toward next-generation optoelectronic and information security technologies.

## Supplementary Material





## Notes

Synthetic details, molecule characterization, theoretical calculations, and photophysical properties (PDF)
Movie showing multicolor emission sequence upon removal of the UV excitation source (MP4)
