# Supplementary material for: Unveiling the Evolution of Afterglow in Diboraanthracene Scaffolds: From Thermally Activated Delayed Fluorescence to Room-Temperature Phosphorescence
Source: J Am Chem Soc. 2025 Nov 28;147(49):45603–17. doi: 10.1021/jacs.5c16948 (PMC12703752; doi:10.1021/jacs.5c16948)
Supplement: Supplementary file 1 [file ja5c16948_si_001.pdf]

Supporting Information

Unveiling the Evolution of Afterglow in Diboraanthracene Scaffolds: From Thermally Activated Delayed Fluorescence to Room-Temperature Phosphorescence

Yi-Kuan Chen,[‖,a] Jian Lei,[‖,b] Po-Cheng Liu,[‖,a] Cheng-Han Lin,[a] Yu-Ming Chen,[a] Wun-Shuo Chang,[a] I-Chia Chen,[a] Liang-Yan Hsu,[c,d,e] and Tien-Lin Wu*,[a,f]

[a]Department of Chemistry, National Tsing Hua University, No. 101, Sec. 2, Kuang-Fu Rd., Hsinchu 300044, Taiwan.
[b]Department of Public Foundation, Chongqing Three Gorges Medical College, Chongqing 404120, China.
[c]Institute of Atomic and Molecular Sciences, Academia Sinica, Taipei 106319, Taiwan.
[d]Department of Chemistry, National Taiwan University, No. 1, Sec. 4, Roosevelt Rd., Taipei 106319, Taiwan.
[e]Physics Division, National Center for Theoretical Sciences, No. 1, Sec. 4, Roosevelt Rd., Taipei 106319, Taiwan.
[f]College of Semiconductor Research, National Tsing Hua University, No. 101, Sec. 2, Kuang-Fu Rd., Hsinchu 300044, Taiwan.

E-mail: tlwu@mx.nthu.edu.tw

[‖]Y.-K. C., J. L. and P.-C. L. contributed equally.

Table of Contents

1. **General method, synthetic and characterization, and NMR spectra** S2
2. **Single-crystal X-ray diffraction** S11
3. **Thermal and electrochemical properties** S12
4. **Photophysical properties** S13
5. **Time-resolved measurements** S15
6. **Computational methods** S24
7. **OLEDs fabrication and measurement** S29
8. **Calculated optimized geometries** S31
9. **References** S56

1. General method, synthetic and characterization, and NMR spectra

General method

All chemicals and reagents were purchased from commercial suppliers without further purification. ^{1}H NMR, ^{13}C NMR spectra were recorded on a Bruker AM-400 spectrometer. Mass spectra were obtained using a JEOL JMS-T200GC AccuTOF GCx instrument. Single-crystal XRD was performed using a Rigaku XtaLAB Synergy DW instrument. Elemental analyses were performed using an analyzer (Vario EL III CHN-OS Rapid, Elementar). The thermogravimetric analysis (TGA) was recorded on Mettler-Toledo, 2-HT (Ramp:10 °C min^{-1} under N_2). The differential scanning calorimetry (DSC) was recorded on TA Q10. IR spectra were recorded using a Bruker Vertex80v FTIR spectrometer equipped with an ATR accessory. Solid powder samples were measured directly without further preparation. UV-vis absorption spectra were recorded on a Hitachi U-3300 spectrophotometer. Fluorescence spectra were recorded on a Hitachi F-7000 spectrophotometer. The LUMO levels of compounds were determined with an electrochemical analyzer. Emission spectra and decay curves were recorded on an Edinburgh FLS980 spectrometer equipped with a xenon arc lamp, a microsecond flashlamp, and a nanosecond flashlamp. Prompt emission decays were measured within a 100 ns time window, while the kinetic scan mode was used to capture complete decay profiles of afterglow emissions. The kinetic scans were performed using a 450 W Xenon lamp at 365 nm, while the photographic and video recordings were carried out under a handheld 18 W UV lamp at 365 nm. Transient EL measurements were performed on FL980 equipped with an arbitrary function generator (AFG 31000, Tektronix). NIR-TAS spectra were recorded on step-scan FT-NIR spectrometer (Vertex80, Bruker). Third harmonic generation of Nd: YAG laser (355 nm) were used as excitation source. All solutions were degassed by freeze-thaw-pump methods for 3 cycles before the TAS measurement. Photographs and videos were captured by Canon EOS M50 or iPhone 13 cameras. The PMMA doped films (1 wt % DBA) were annealed at 90 °C for 30 minutes under a nitrogen atmosphere and then allowed to cool naturally to room temperature.

Synthesis and characterization

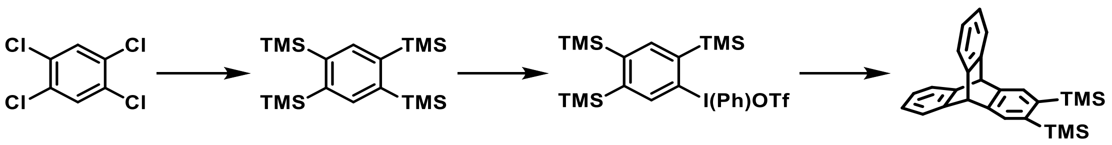

Synthesis of 2,3-bis(trimethylsilyl)-9,10-dihydro-9,10-[1,2]benzenoanthracene. 2,3-bis(trimethylsilyl)-9,10-dihydro-9,10-[1,2]benzenoanthracene was obtained by the reported methods.[1] The total yield was 29.3%.

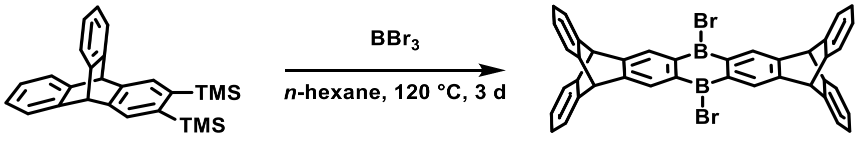

Synthesis of 9,10-dibromo-9,10-diborapentiptycene (DBDBPI). 2,3-bis(trimethylsilyl)-9,10-dihydro-9,10-[1,2]benzenoanthracene (1 g, 2.51 mmol) and boron tribromide (2.2 g, 8.78 mmol) were dissolved in dry *n*-hexane

(7.4 mL). Then the solution was stirred and heated at 120 °C for 3 d. After cooling, the excess BBr_3 and solvent was removed under reduced pressure and the brown solid was used for the next step without further purification. We failed to obtain the NMR spectrum due to its poor solubility in common deuterated solvent.

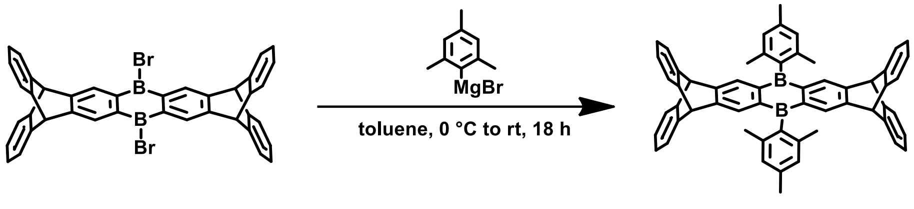

MesDBPI (25%)

Synthesis of MesDBPI. DBDBPI was dissolved in dry toluene (1.25 mmol, 32 mL). At 0 °C, 2-mesitylmagnesium bromide solution (2.6 mL, 1 M in THF) was added dropwise to the solution of DBDBPI, and the temperature was slowly returned to room temperature. After 18 hours, the reaction mixture was quenched with saturated ammonium chloride solution and extracted by DCM. The solution was dried over magnesium sulfate and evaporated under reduced pressure. Then the mixture was purified by column chromatography on silica gel (hexane/DCM, 4:1) and gradient-temperature sublimation method to afford a yellow solid **MesDBPI**. The total yield of two steps was 25%. ^{1}H NMR (400 MHz, $CDCl_3$, δ): 1.91 (s, 12H), 2.46 (s, 6H), 5.35 (s, 4H), 6.93-6.95 (m, 12H), 7.28-7.30 (m, 8H), 7.44 (s, 4H). ^{13}C NMR (100 MHz, $CDCl_3$, δ): 21.39, 22.83, 53.95, 123.83, 125.21, 126.76, 133.50, 136.52, 138.44, 141.43, 143.46, 144.65, 150.06. HR-MS (FD): m/z 764.37863, calcd for $C_{58}H_{46}B_2$; 764.37911. Anal. Calcd. For $C_{58}H_{46}B_2$: C, 91.11; H, 6.06 found: C, 91.00; H, 6.08. Melting point: 460 °C (dec).

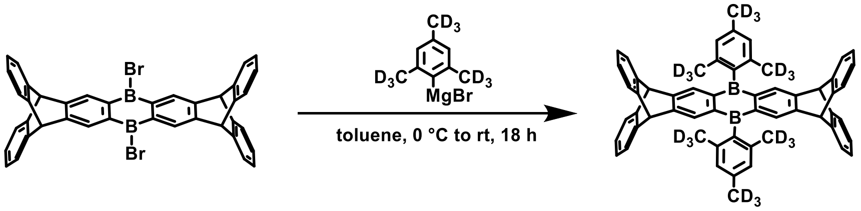

MesDBPI-d_{18} (26%)

Synthesis of MesDBPI-d$_{18}$. DBDBPI was dissolved in dry toluene (1.00 mmol, 26 mL). At 0 °C, 2,4,6-tris(methyl-d$_3$)phenylmagnesium bromide solution (2.1 mL, 1 M in THF) was added dropwise to the solution of DBDBPI, and the temperature was slowly returned to room temperature. After 18 hours, the reaction mixture was quenched with saturated ammonium chloride solution and extracted by DCM. The solution was dried over magnesium sulfate and evaporated under reduced pressure. Then the mixture was purified by column chromatography on silica gel (hexane/DCM, 4:1) and gradient-temperature sublimation method to afford a yellow solid **MesDBPI-d$_{18}$**. The total yield of two steps was 26%. ^{1}H NMR (400 MHz, $CDCl_3$, δ): 1.88 (s, 1.22H, 90% D), 2.44 (s, 0.59H, 90% D), 5.36 (s, 4H), 6.93-6.95 (m, 12H), 7.27-7.30 (m, 8H), 7.44 (s, 4H). ^{13}C NMR (100 MHz, $CDCl_3$, δ): 20.57, 21.78, 21.97, 22.16, 22.46, 53.96, 123.83, 125.21, 126.77, 133.52, 136.41, 138.33, 141.47, 143.48, 144.66, 150.07. HR-MS (FD): m/z 782.48339, calcd for $C_{58}H_{28}D_{18}B_2$; 782.48321. Anal. Calcd. For $C_{58}H_{28}D_{18}B_2$: C, 89.00; H, 8.24 found: C, 89.03; H, 8.14. Melting point: 465 °C (dec).

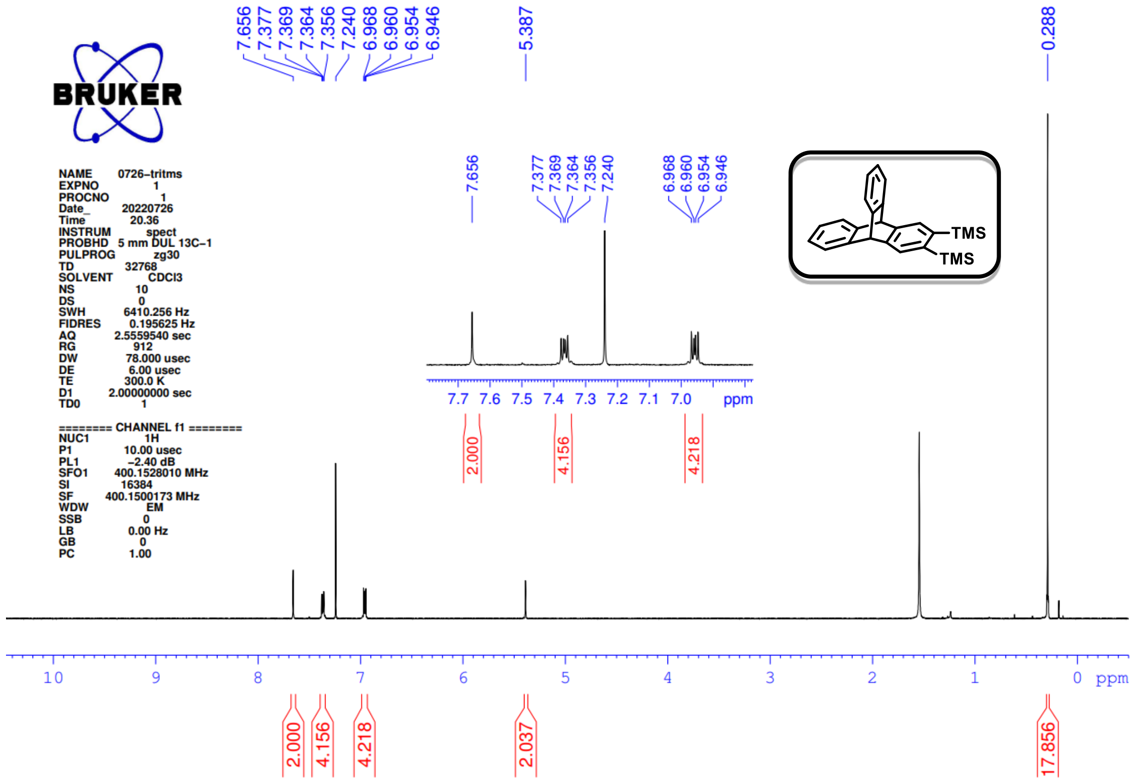

Figure S1. The ^{1}H NMR of 2,3-bis(trimethylsilyl)-9,10-dihydro-9,10-[1,2]benzenoanthracene in $CDCl_3$

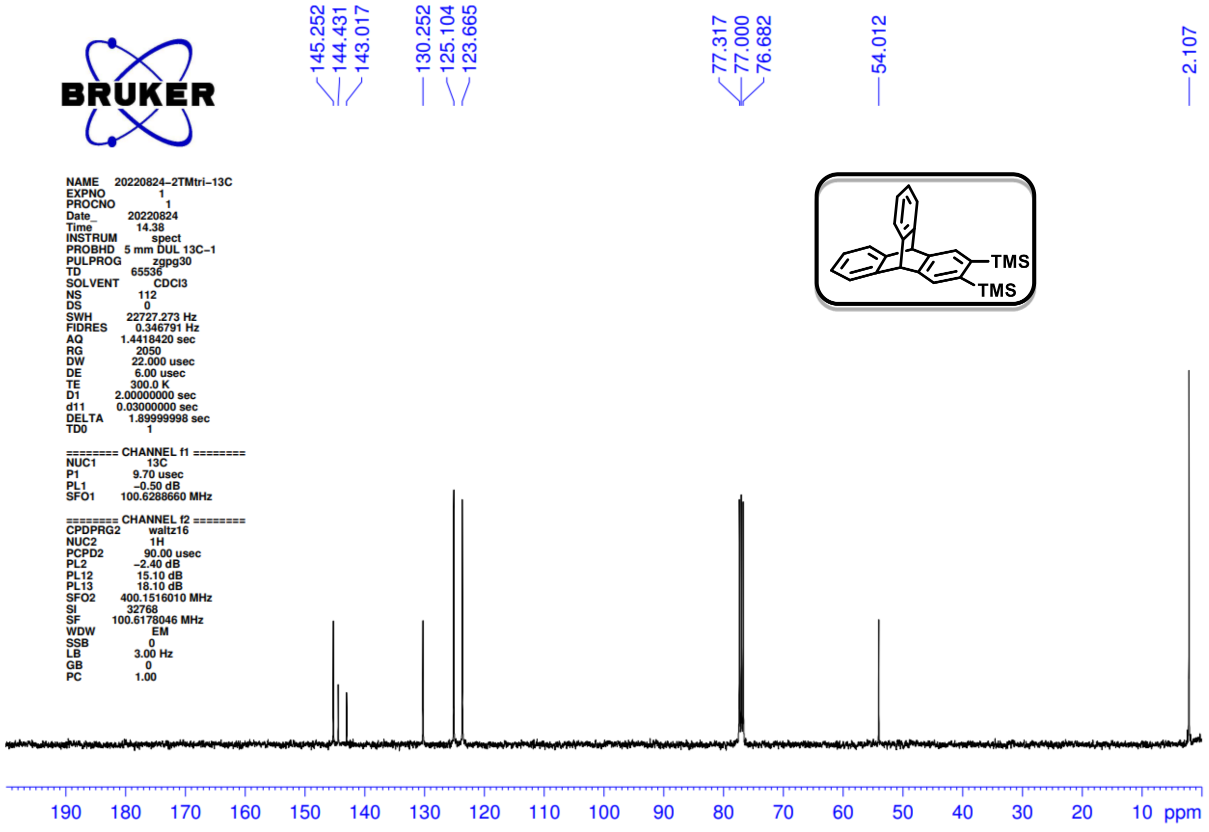

Figure S2. The ^{13}C NMR of 2,3-bis(trimethylsilyl)-9,10-dihydro-9,10-[1,2]benzenoanthracene in $CDCl_3$

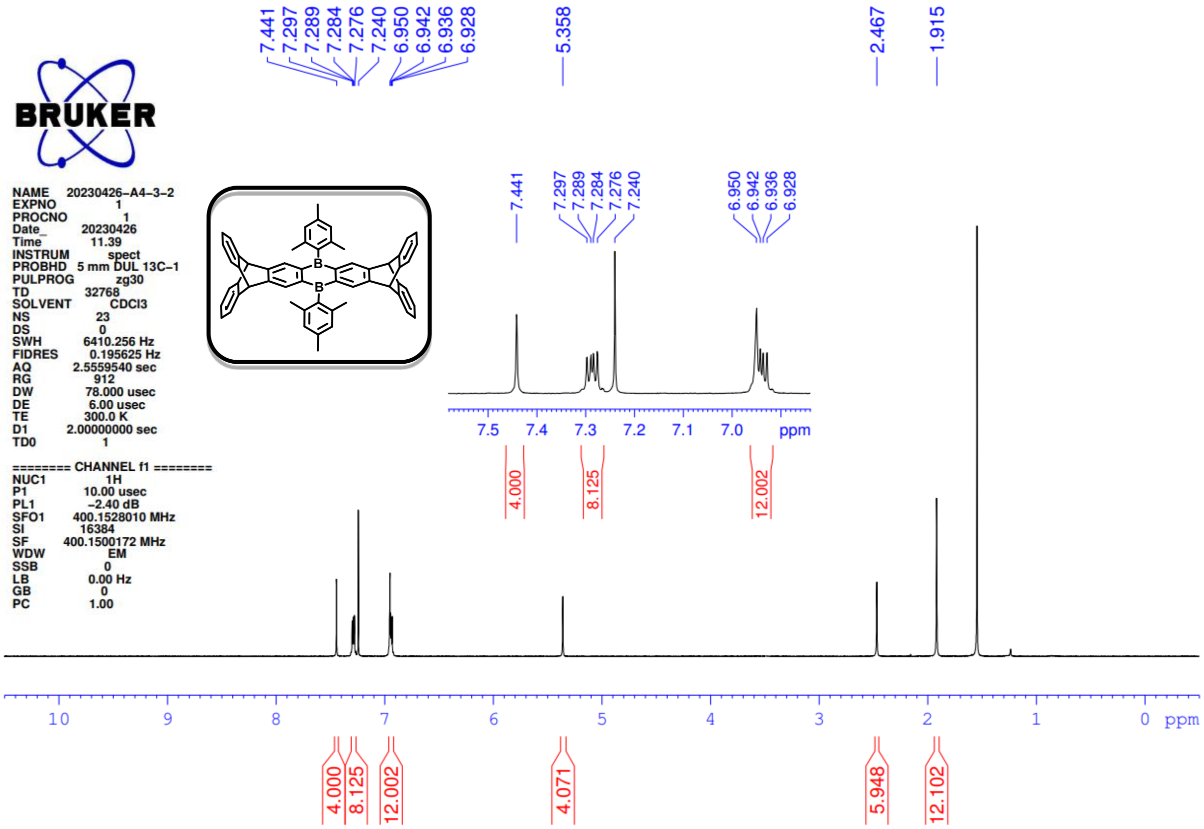

Figure S3. The ^{1}H NMR of MesDBPI in $CDCl_3$.

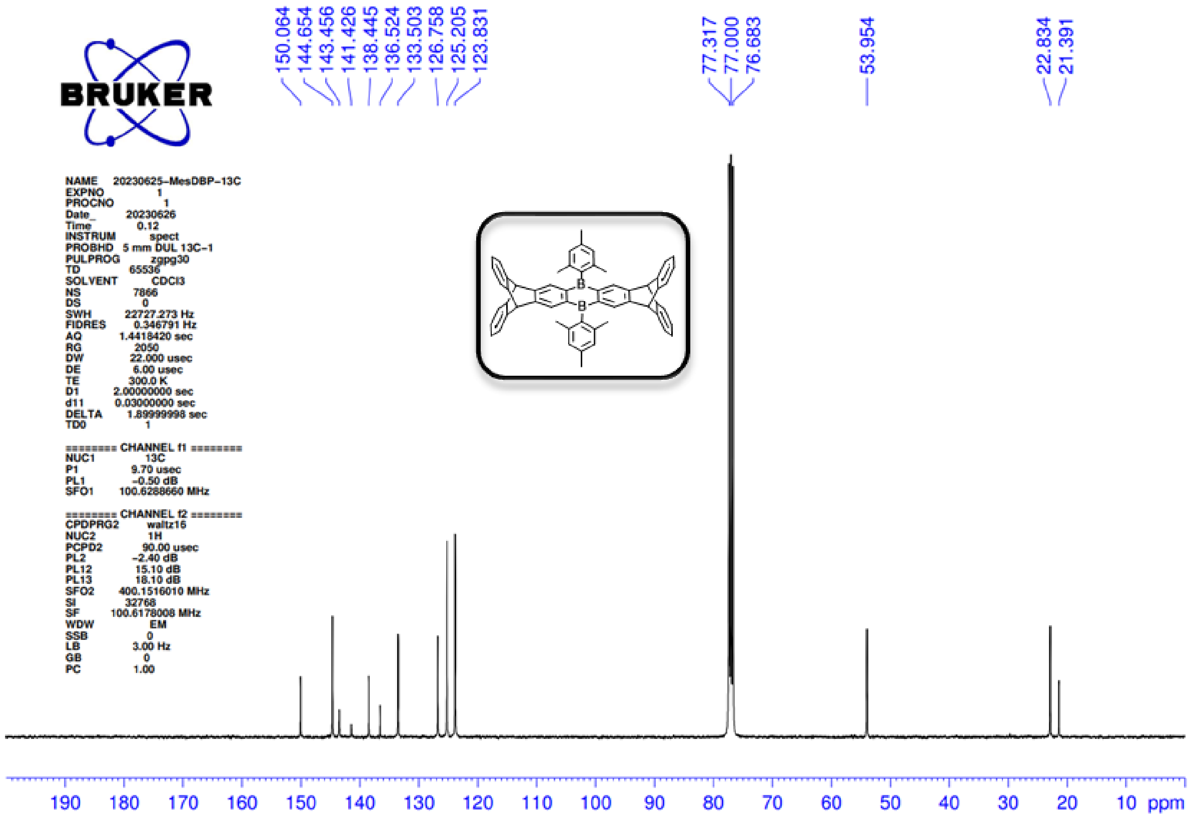

Figure S4. The ^{13}C NMR of MesDBPI in $CDCl_3$.

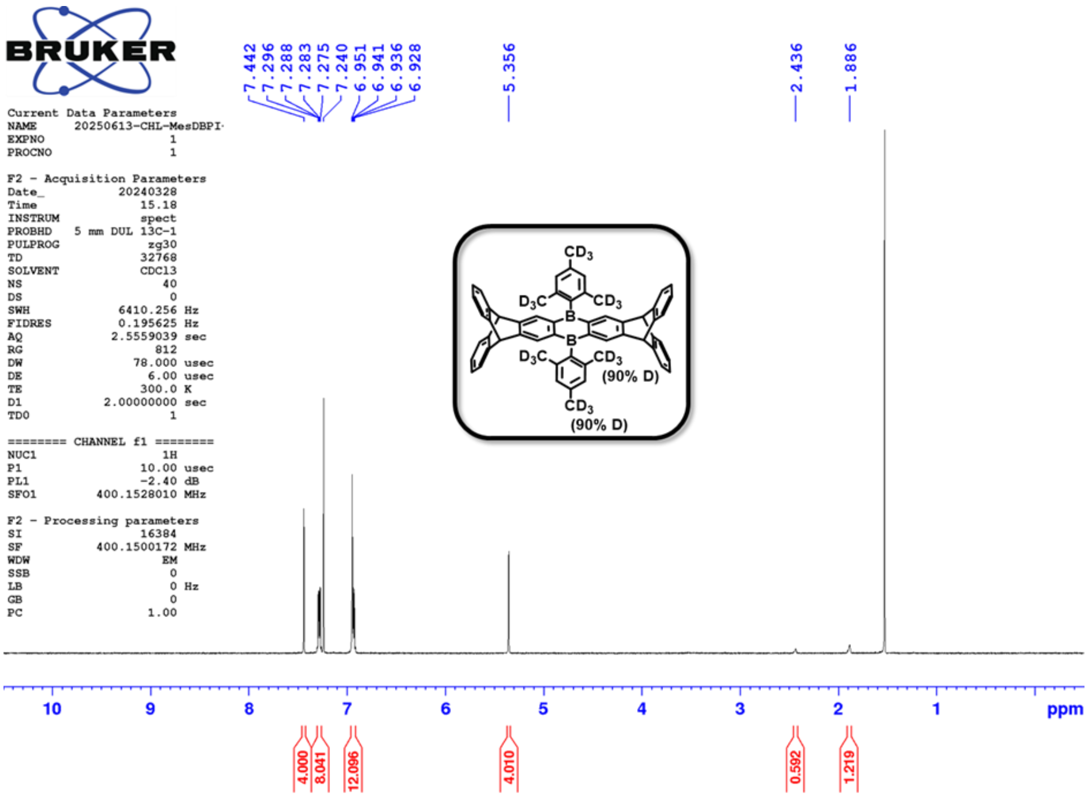

Figure S5. The ^{1}H NMR of MesDBPI-d_{18} in $CDCl_3$.

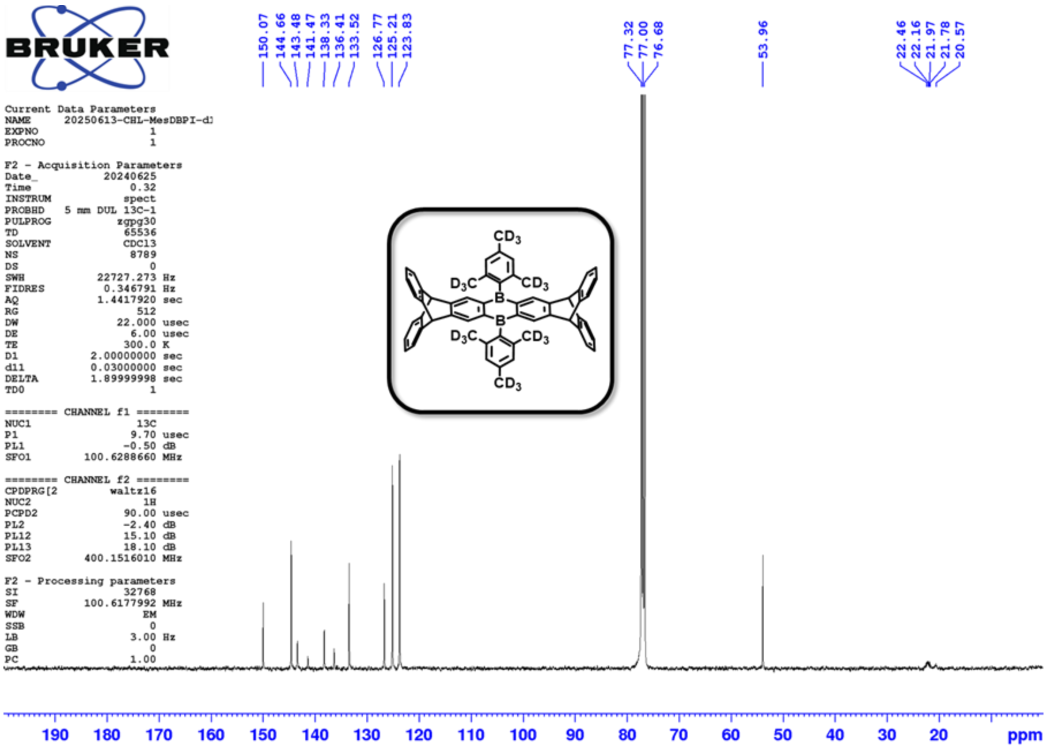

Figure S6. The ^{13}C NMR of MesDBPI-d_{18} in $CDCl_3$.

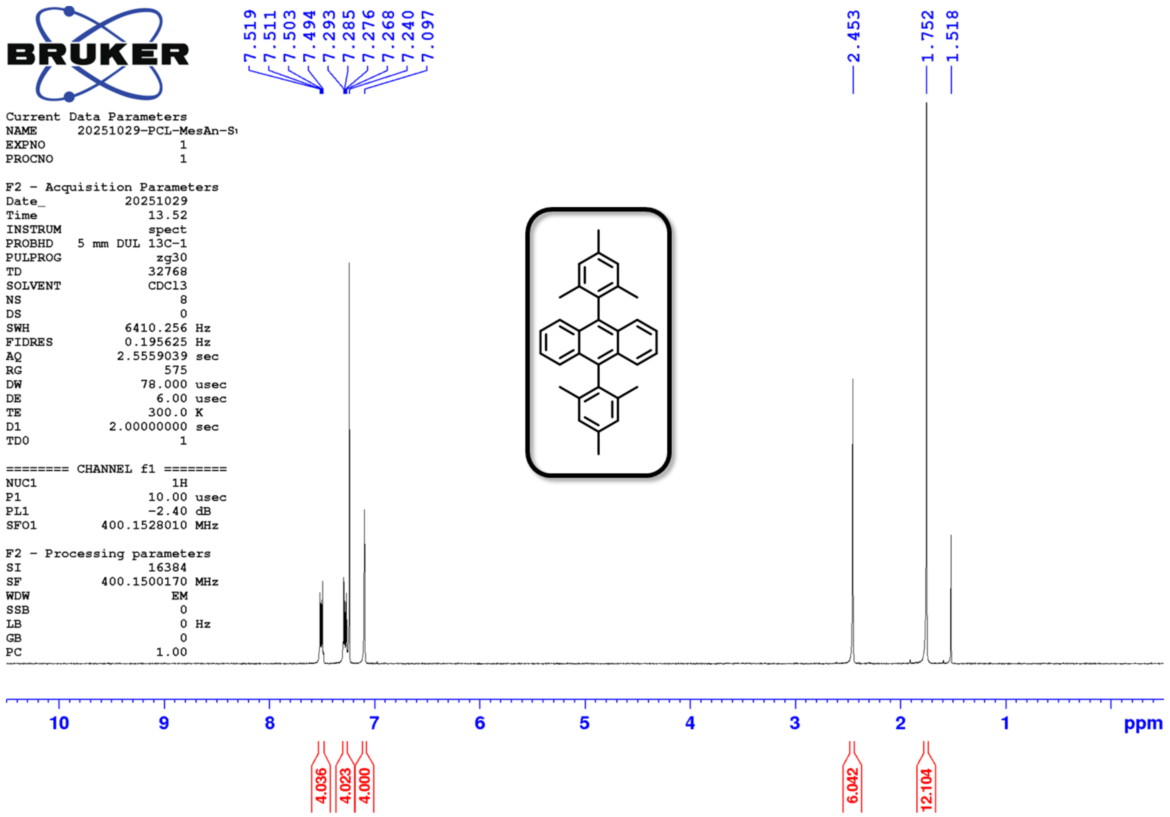

Figure S7. The ^{1}H NMR of MesAn in $CDCl_3$.

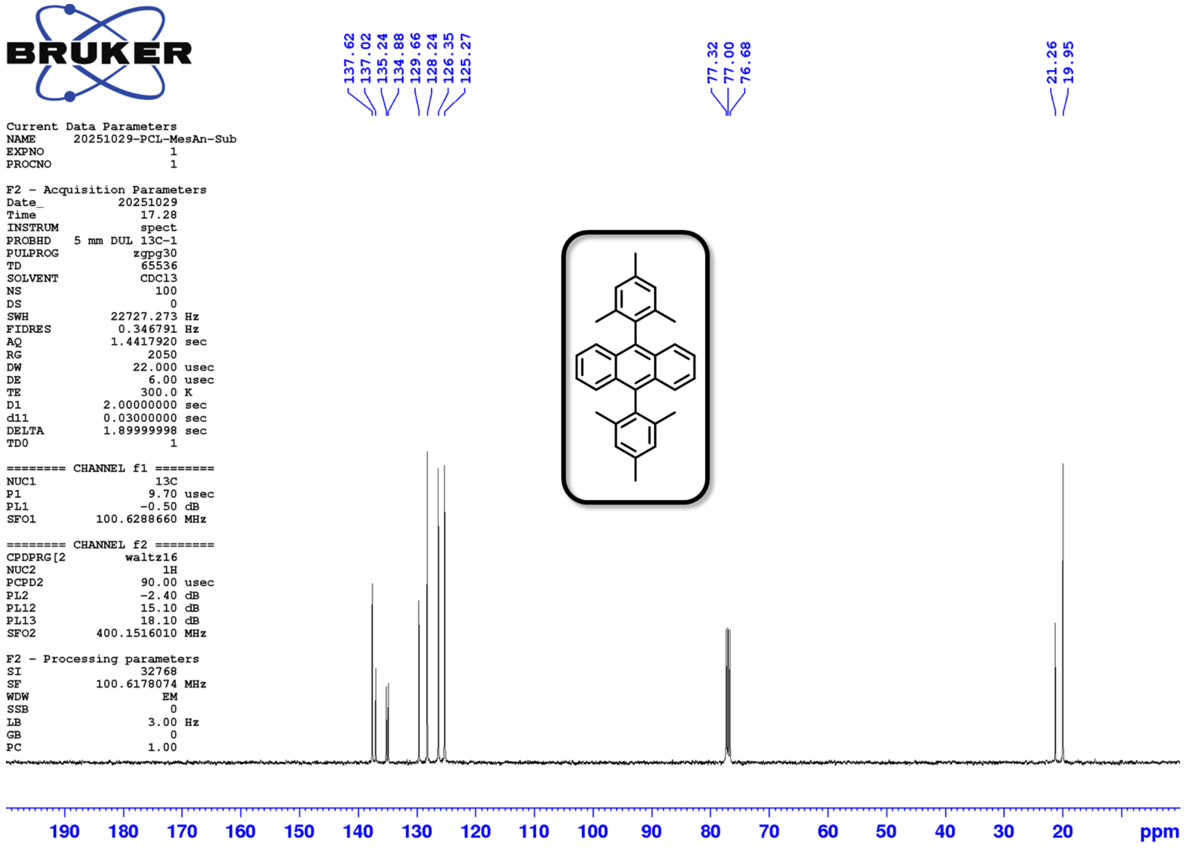

Figure S8. The ^{13}C NMR of MesAn in $CDCl_3$.

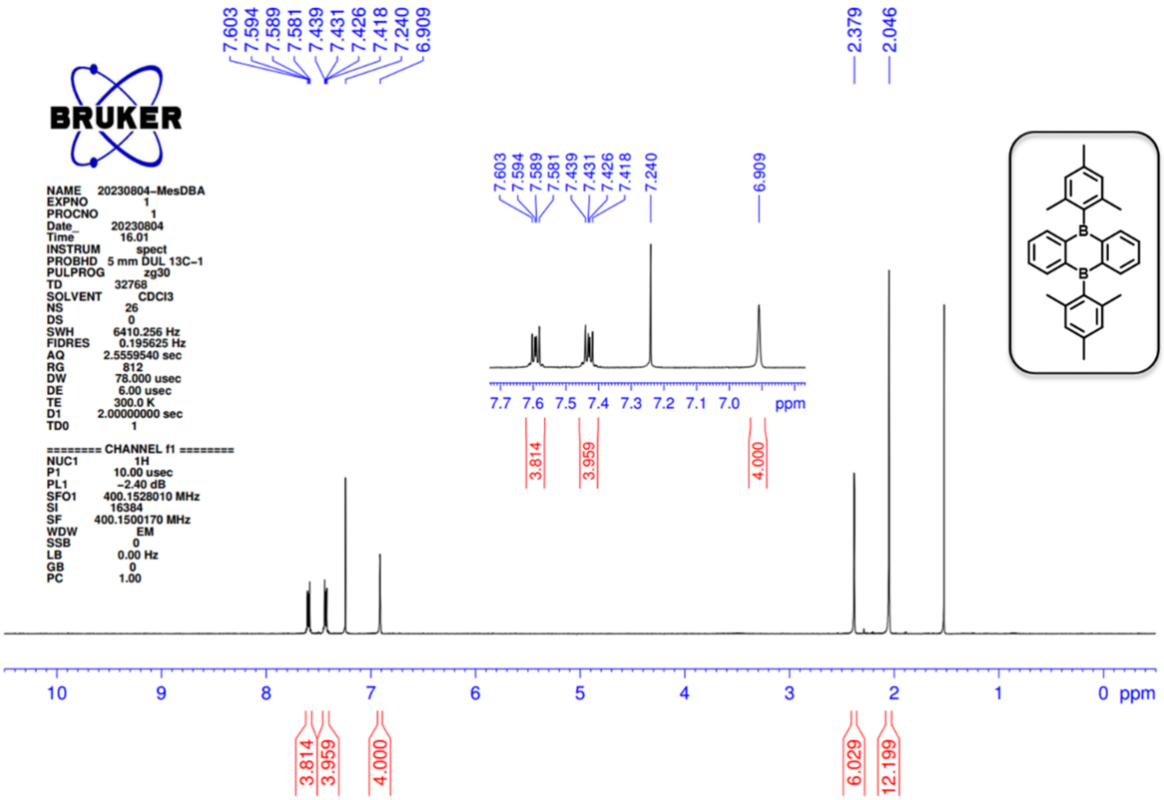

Figure S9. The ^{1}H NMR of MesDBA in $CDCl_3$.

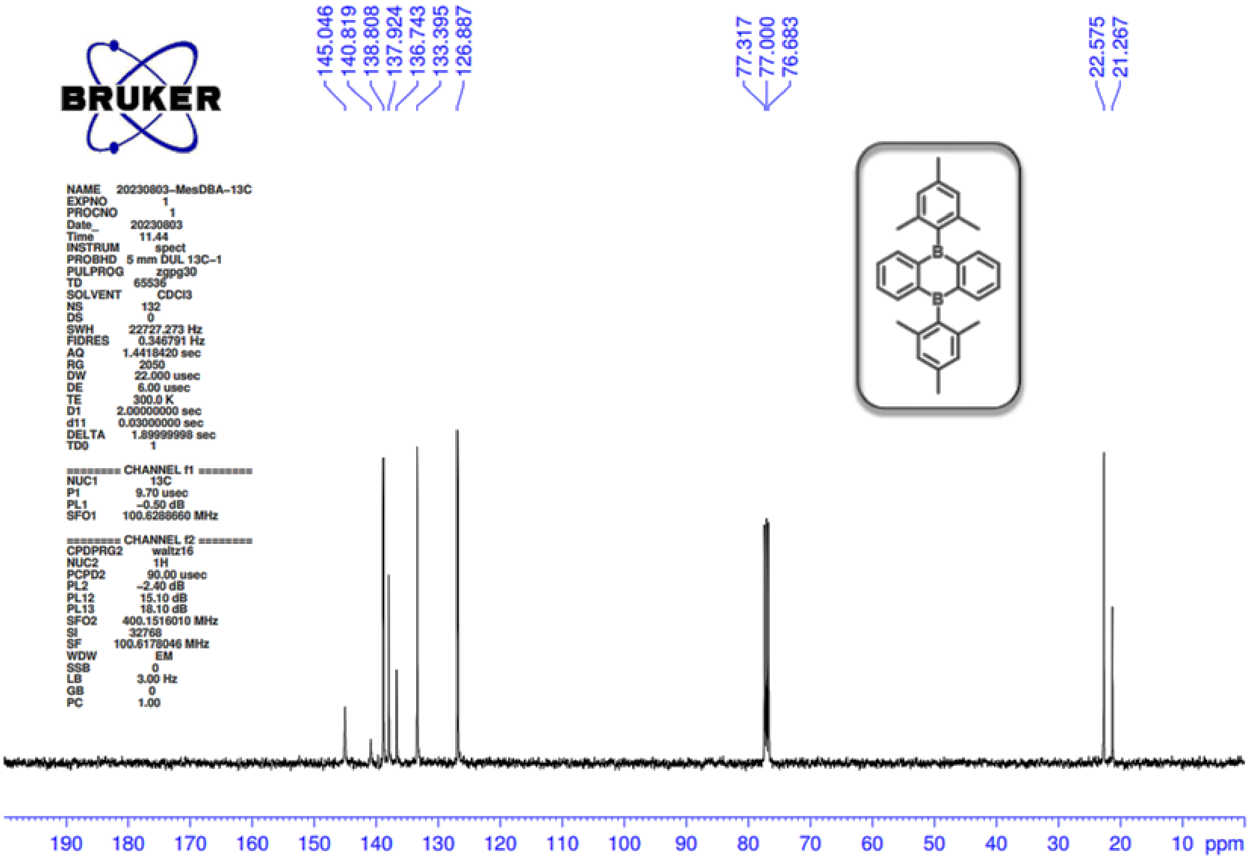

Figure S10. The ^{13}C NMR of MesDBA in $CDCl_3$.

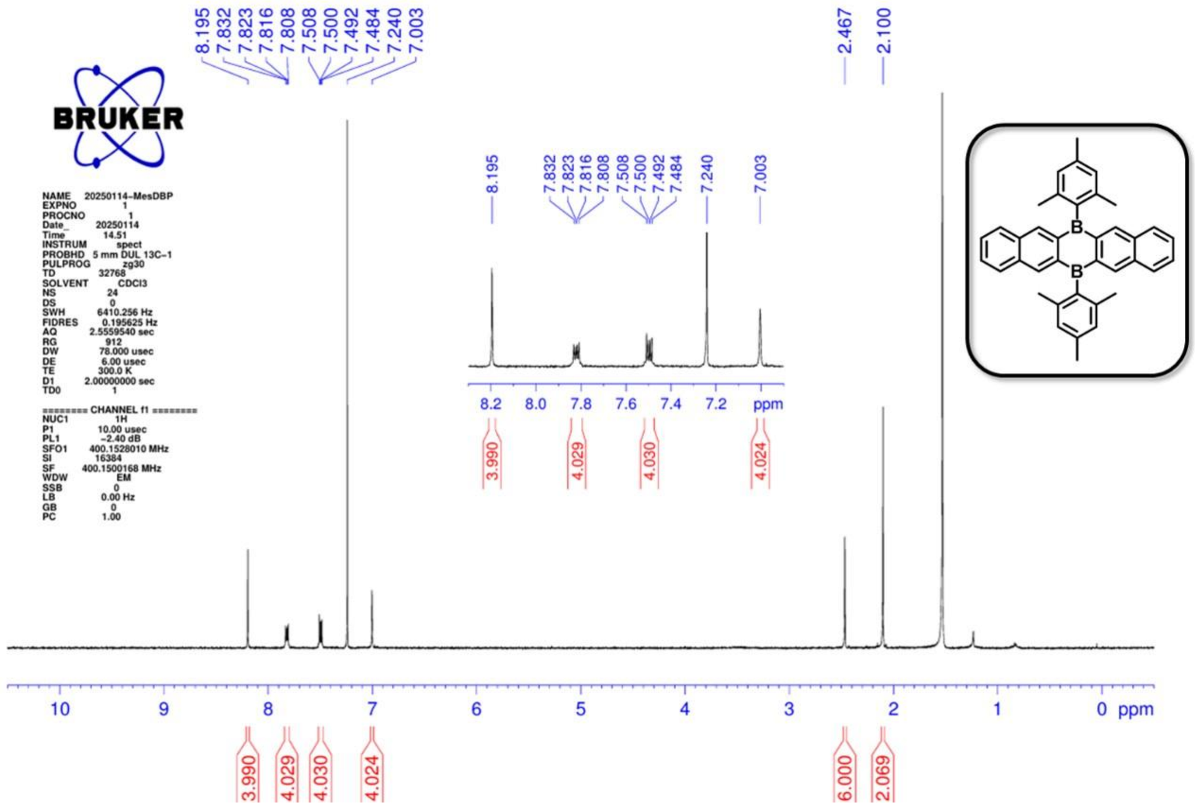

Figure S11. The ^{1}H NMR of MesDBP in $CDCl_3$.

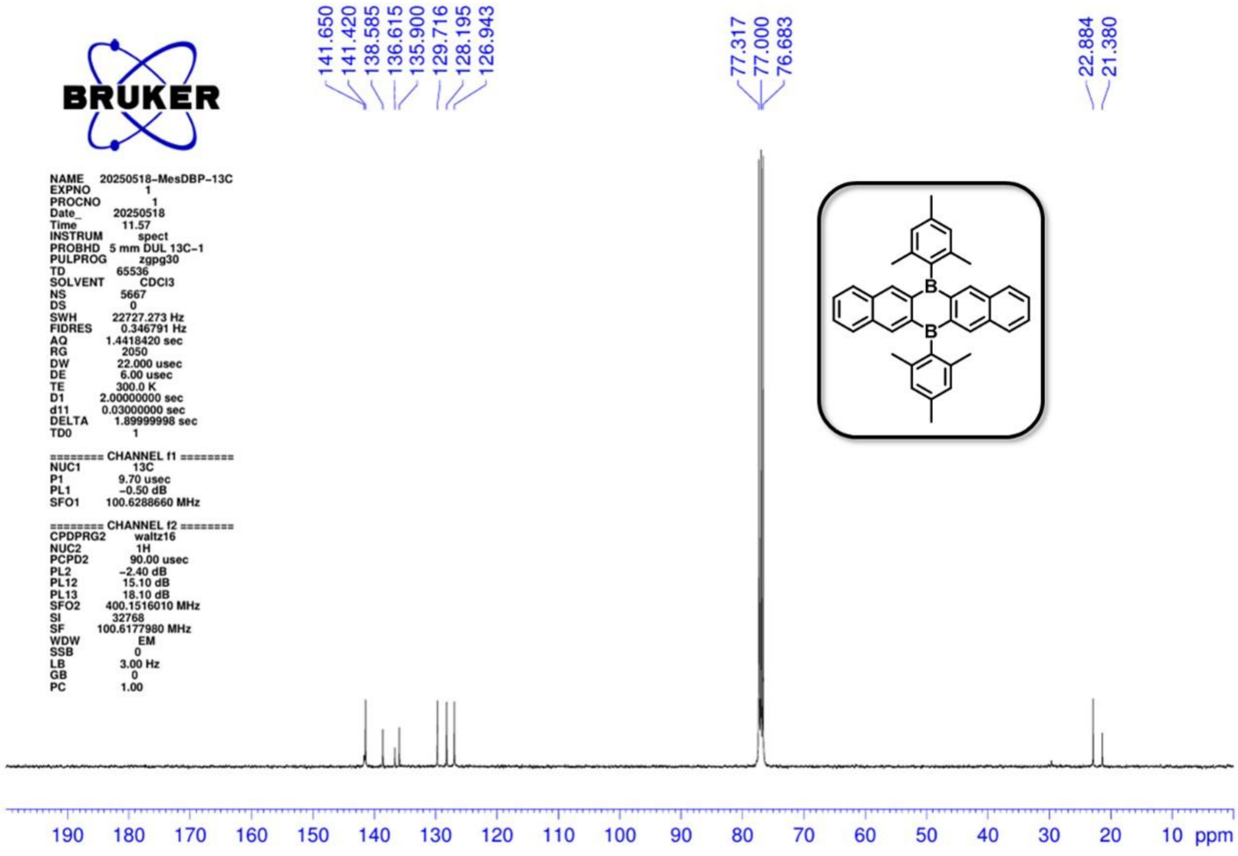

Figure S12. The ^{13}C NMR of MesDBP in $CDCl_3$.

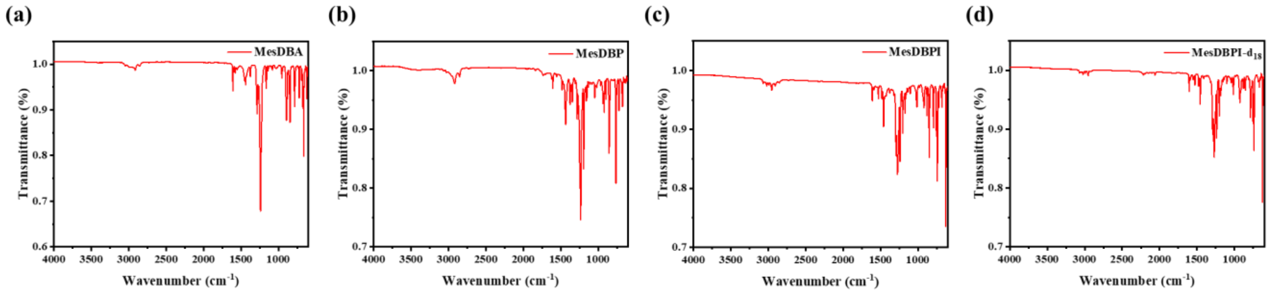

Figure S13. IR spectroscopy of **(a)** MesDBA, **(b)** MesDBP, **(c)** MesDBPI and **(d)** MesDBPI-d_{18}.

MesDBA: FTIR (cm^{-1}): 3043, 3000, 2913, 2853, 1608, 1439, 1376, 1284, 1238, 1163, 954, 891, 844, 784, 722, 673, 662, 621.
MesDBP: FTIR (cm^{-1}): 3027, 2919, 2852, 1607, 1483, 1435, 1376, 1345, 1282, 1234, 1193, 1159, 1147, 1046, 918, 897, 855, 847, 763, 722, 674.
MesDBPI: FTIR (cm^{-1}): 3068, 3021, 2990, 2954, 2908, 2853, 1610, 1528, 1481, 1458, 1292, 1275, 1239, 1203, 1171, 1014, 920, 879, 849, 794, 753, 741, 722, 678, 628.
MesDBPI-d_{18}: FTIR (cm^{-1}): 3069, 3020, 2990, 2953, 2216, 2058, 1605, 1528, 1482, 1459, 1292, 1270, 1238, 1202, 1182, 1015, 929, 883, 857, 788, 753, 740, 670, 628.

2. Single-crystal X-ray diffraction

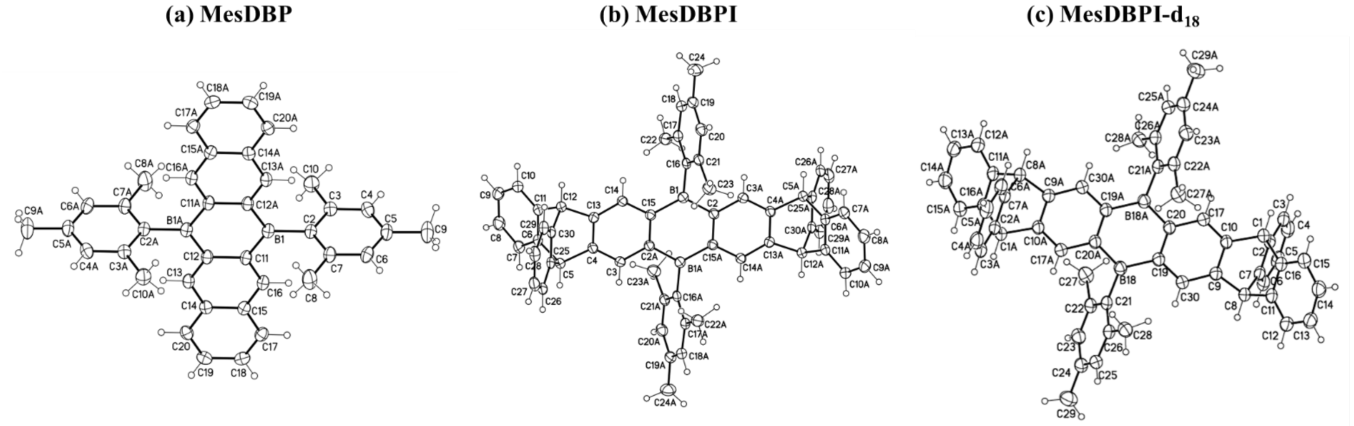

Figure S14. The single-crystal structure of **(a)** MesDBP, **(b)** MesDBPI and **(c)** MesDBPI-d_{18}.

Table S1. Crystal data and structure refinement for MesDBP, MesDBPI and MesDBPI-d_{18}

Molecule	MesDBP	MesDBPI	MesDBPI-d_{18}
Empirical formula	$C_{38}H_{34}B_2$	$C_{58}H_{46}B_2$	$C_{58}H_{28}B_2D_{18}$
Formula weight	512.27	764.57	782.68
Temperature/K	99.97(15)	100.00(10)	100.00(10)
Crystal system	trigonal	triclinic	triclinic
Space group	R-3	P-1	P-1
a/Å	30.3037(3)	8.2343(3)	8.2249(4)
b/Å	30.3037(3)	11.8265(5)	11.8251(5)
c/Å	8.50810(10)	12.8263(3)	12.8083(6)
α/°	90	64.328(3)	64.301(4)
β/°	90	85.252(2)	85.202(4)
γ/°	120	70.854(3)	70.874(4)
Volume/Å^3	6766.35(16)	1060.90(7)	1058.06(9)
Z	9	2	1
ρ_{calc}g/cm^3	1.131	1.197	1.228
μ/mm^{-1}	0.468	0.502	0.504
F(000)	2448.0	404.0	404.0
Crystal size/mm^3	0.1 × 0.05 × 0.04	0.14 × 0.12 × 0.11	0.07 × 0.05 × 0.04
Radiation	Cu Kα (λ = 1.54184)	Cu Kα (λ = 1.54184)	Cu Kα (λ = 1.54184)
2Θ range for data collection/°	10.112 to 148.876	7.666 to 134.146	7.678 to 146.118
Index ranges	-35 ≤ h ≤ 37, -37 ≤ k ≤ 35, -10 ≤ l ≤ 9	-9 ≤ h ≤ 9, -14 ≤ k ≤ 13, -15 ≤ l ≤ 13	-9 ≤ h ≤ 9, -14 ≤ k ≤ 9, -15 ≤ l ≤ 15
Reflections collected	7362	12229	13040
Independent reflections	7362 [R_{int} = ?, R_{sigma} = 0.0115]	3765 [R_{int} = 0.0199, R_{sigma} = 0.0212]	4035 [R_{int} = 0.0256, R_{sigma} = 0.0341]
Data/restraints/parameters	7362/0/185	3765/0/275	4035/0/275
Goodness-of-fit on F^2	1.039	1.066	1.065
Final R indexes [I>=2σ (I)]	R_1 = 0.0470, wR_2 = 0.1289	R_1 = 0.0363, wR_2 = 0.0933	R_1 = 0.0444, wR_2 = 0.1237
Final R indexes [all data]	R_1 = 0.0543, wR_2 = 0.1344	R_1 = 0.0406, wR_2 = 0.0965	R_1 = 0.0589, wR_2 = 0.1344
Largest diff. peak/hole / e Å^{-3}	0.24/-0.21	0.21/-0.18	0.19/-0.19

3. Thermal and electrochemical properties

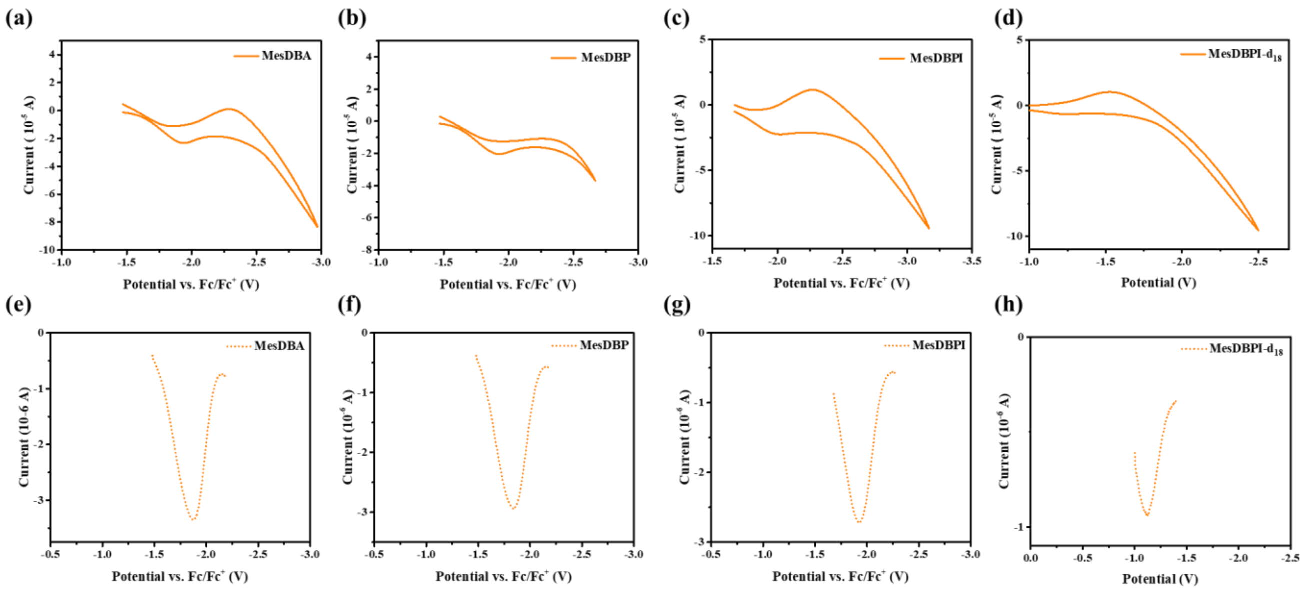

Figure S15. The cyclic voltammetry (CV) for **(a)** MesDBA, **(b)** MesDBP, **(c)** MesDBPI and **(d)** MesDBPI-d_{18}. The differential pulse voltammetry (DPV) for **(e)** MesDBA, **(f)** MesDBP, **(g)** MesDBPI and **(h)** MesDBPI-d_{18}. All measurements were conducted in anhydrous THF with ferrocene as the standard.

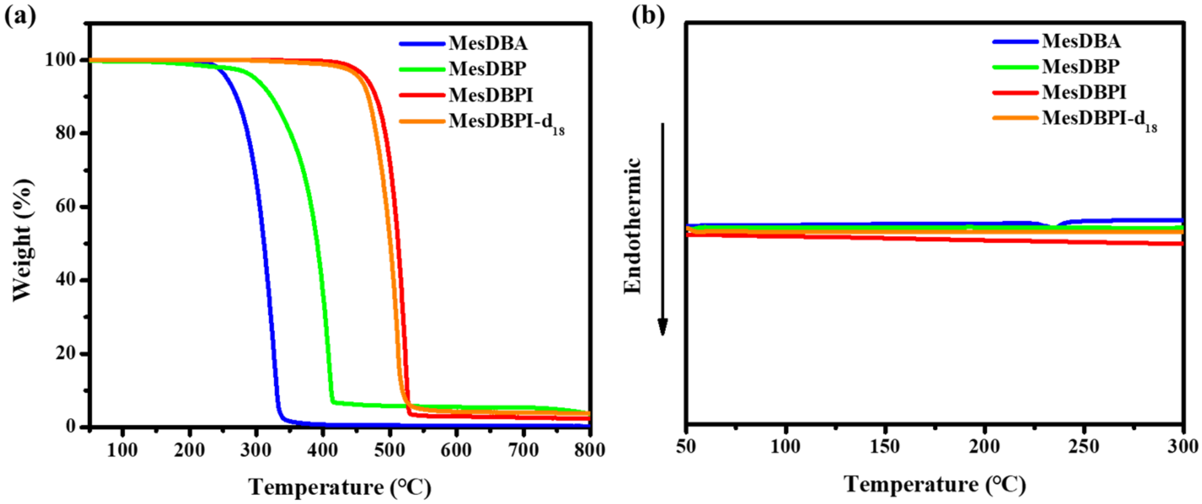

Figure S16. (a) Thermal gravimetric analysis and **(b)** differential scanning calorimetry (DSC) analysis of MesDBA, MesDBP, MesDBPI and MesDBPI-d_{18}.

4. Photophysical properties

Table S2. Summary of the photophysical properties of four emitters

Emitter	λ_{abs}[a] [nm]	λ_{fl}[a] [nm]	FWHM[b] [nm]	S_1[c] [eV]	T_1[c] [eV]	ΔE_{ST}[c] [eV]	HOMO[d] [eV]	LUMO[d] [eV]	E_g[d] [eV]	T_d[e] [°C]	T_g[e] [°C]
MesDBA	350	455,	68	3.02	2.66	0.36	-5.51	-2.68	2.83	256	235
MesDBP	304, 410	417, 441, 469	12	3.07	2.50	0.57	-5.66	-2.70	2.96	298	N.D.
MesDBPI	290, 358	481	56	2.91	2.39	0.52	-5.50	-2.65	2.85	464	N.D.
MesDBPI-d_{18}	290, 358	482	57	2.93	2.40	0.53	-5.48	-2.66	2.82	435	N.D.

[a]Absorption (abs) and fluorescence (fl) emission peak in toluene (10^{-4} M). [b]Full-width at half-maximum (FWHM) of fluorescence in toluene (10^{-4} M). [c] S_1 and T_1 energy levels were obtained from the onset of the fluorescence and phosphorescence spectra and $\Delta E_{ST} = S_1 - T_1$ in 1 wt % doped PMMA films. [d]The LUMO energy levels were determined from cyclic voltammetry, HOMO = LUMO - E_g. Bandgap calculated from onset of UV–visible absorption. [e]Determined from TGA and DSC.

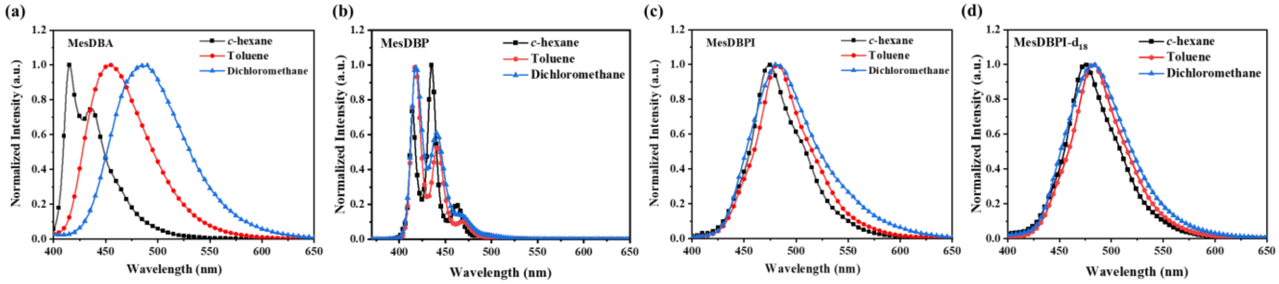

Figure S17. PL spectra of **(a)** MesDBA, **(b)** MesDBP, **(c)** MesDBPI and **(d)** MesDBPI-d_{18} measured in various solvents.

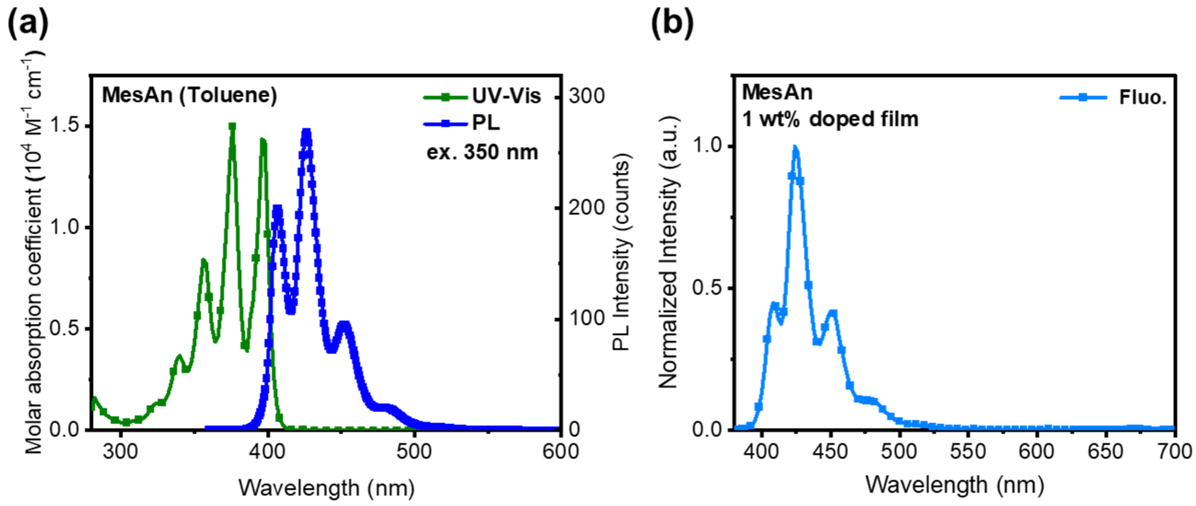

Figure S18. **(a)** The UV/Vis absorption and fluorescence spectra of MesAn in toluene (10^{-4} M). **(b)** Fluorescence (300 K) spectrum of a 1 wt % MesAn-doped PMMA film.

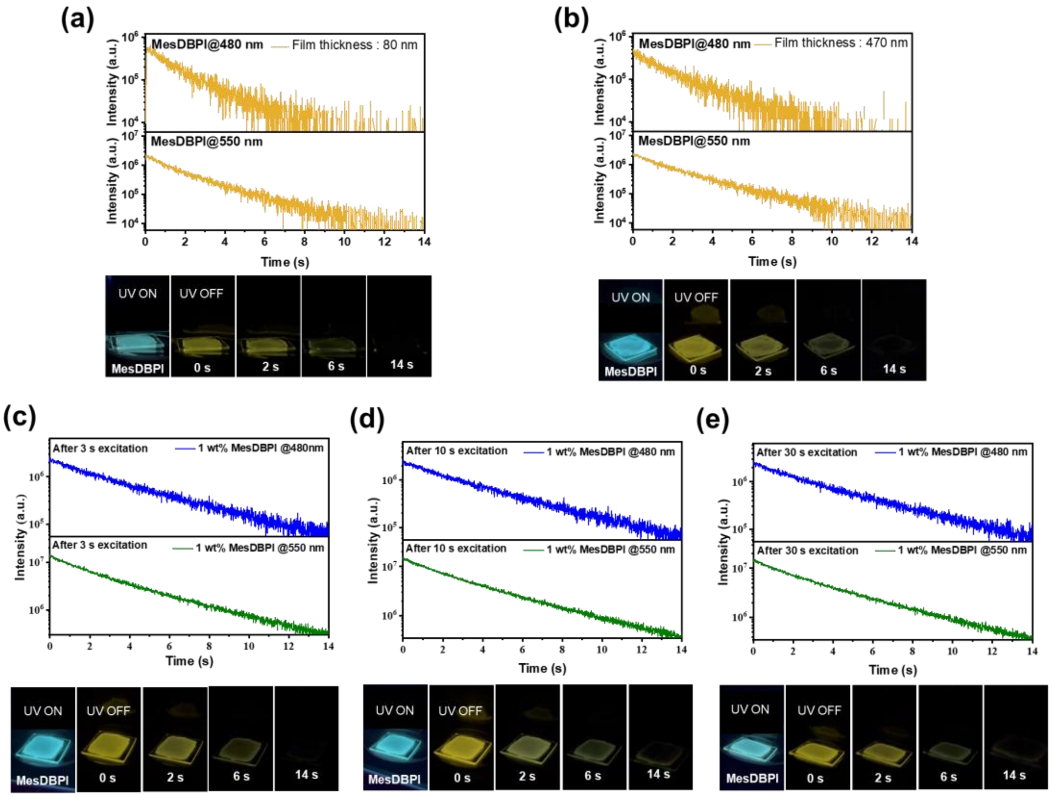

Figure S19. PL decay curves and photographs of 1 wt % MesDBPI-doped PMMA films: **(a)** thickness of 80 nm; **(b)** thickness of 470 nm; **(c)** after 3 s excitation; **(d)** after 10 s excitation; **(e)** after 30 s excitation.

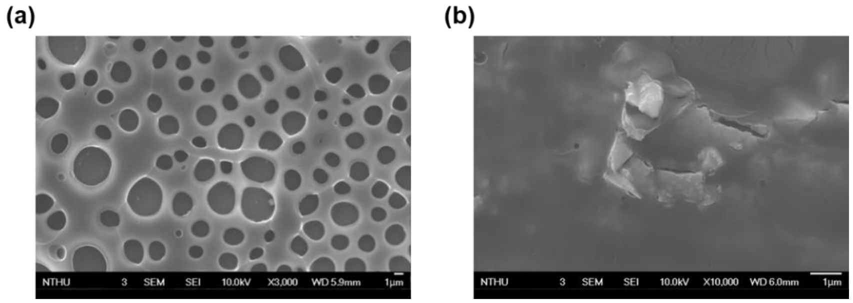

Figure S20. SEM images of PMMA films doped with 1 wt % MesDBPI: **(a)** as-cast and **(b)** after thermal annealing.

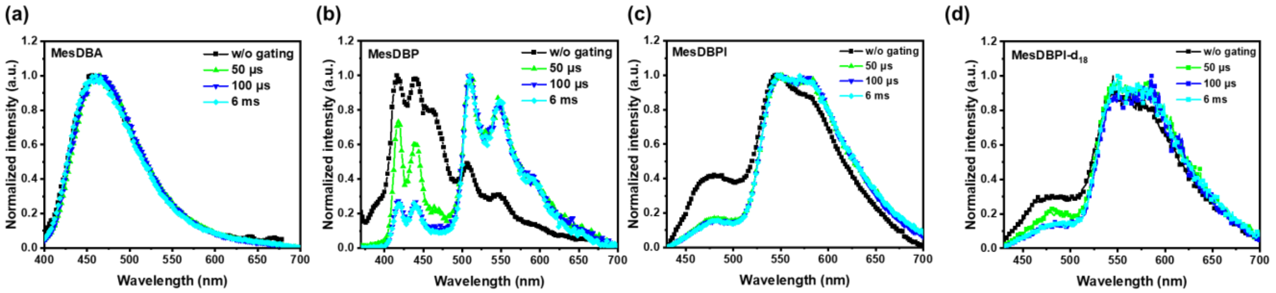

Figure S21. Time-gated PL spectra of four 1 wt % DBAs doped PMMA films after annealing.

5. Time-resolved measurements

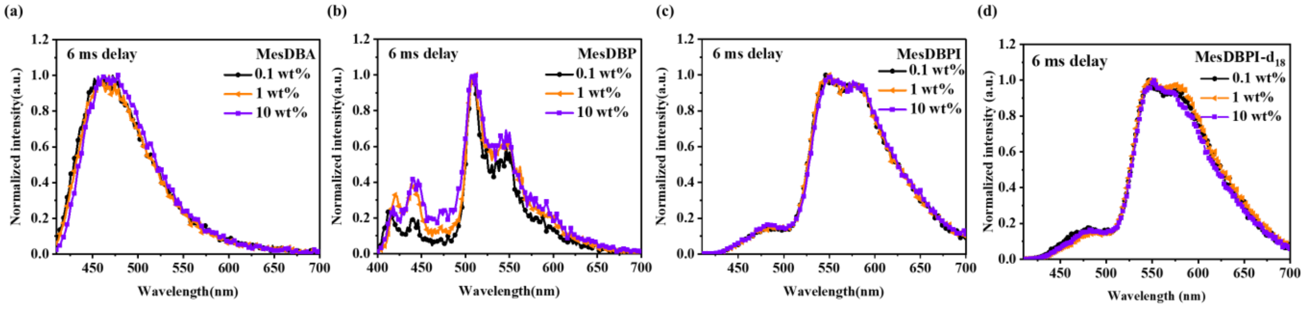

Figure S22. PL spectra obtained at 6 ms delay of **(a)** MesDBA, **(b)** MesDBP, **(c)** MesDBPI and **(d)** MesDBPI-d_{18} in PMMA matrix at different concentrations.

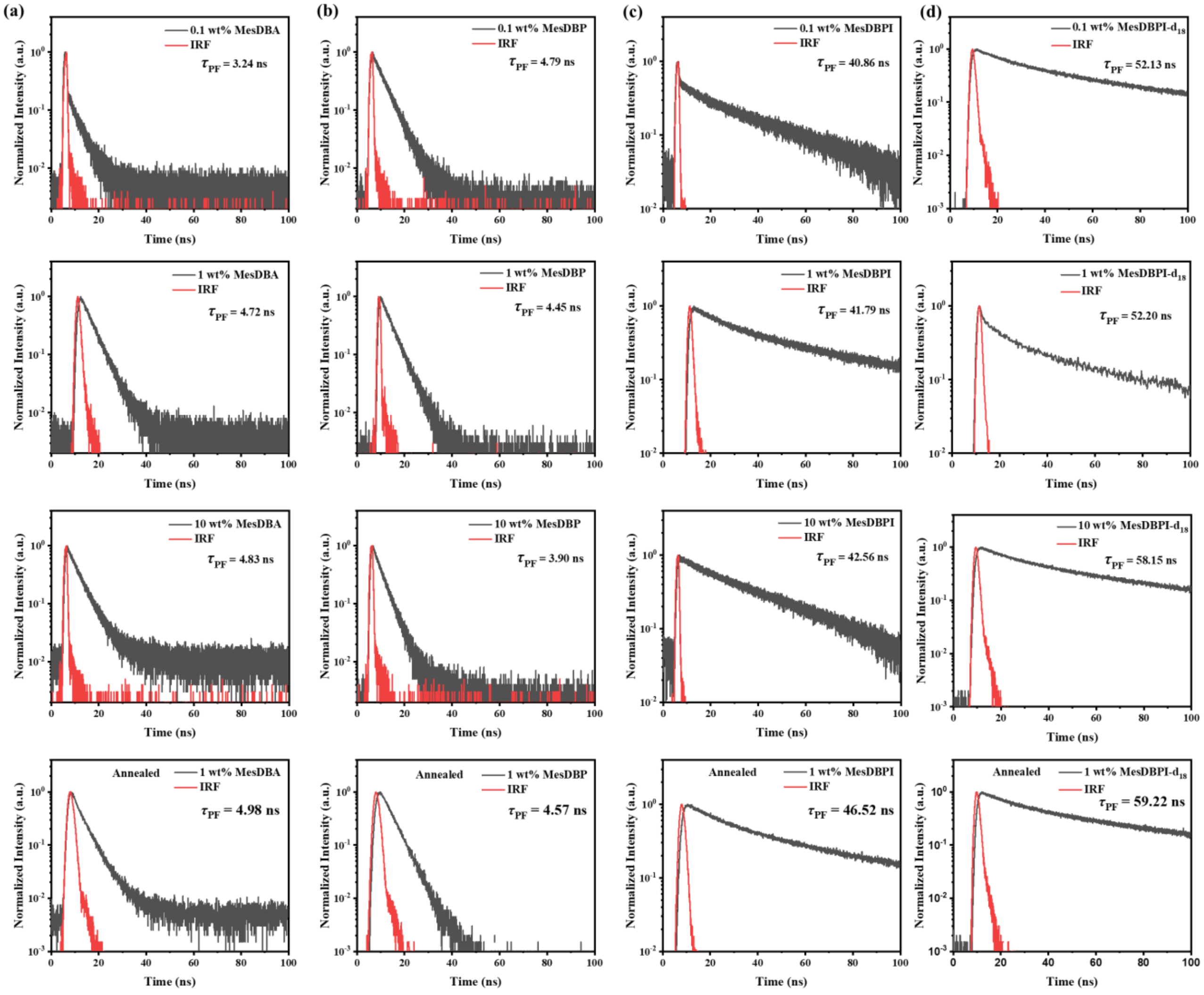

Figure S23. Transient prompt decay curves of (a) MesDBA, (b) MesDBP, (c) MesDBPI and (d) MesDBPI-d_{18} in 0.1, 1, 10 wt % doped PMMA films and their 1 wt % annealed films, where IRF is the instrument response function.

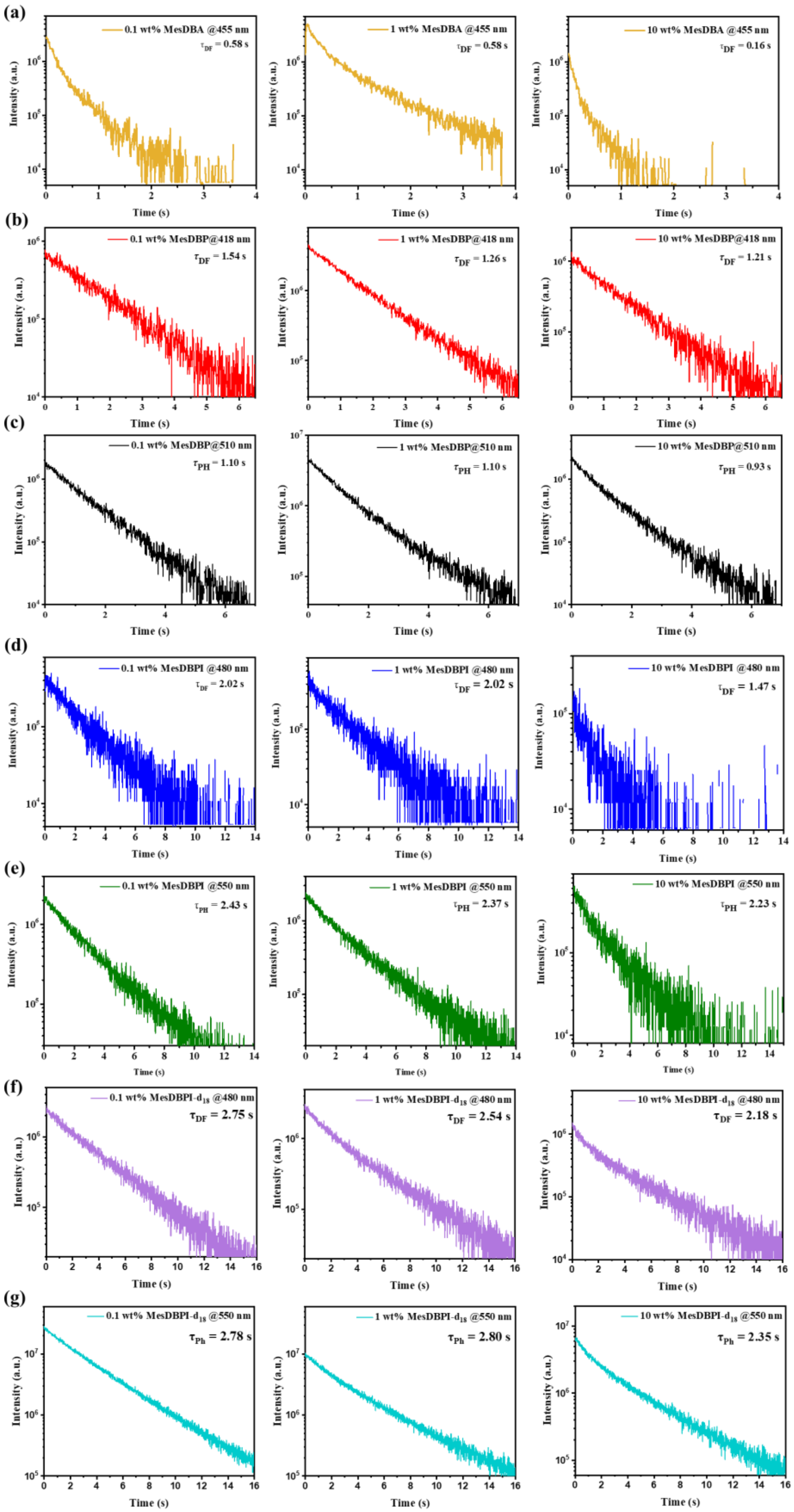

Figure S24. Transient PL decay curves of **(a)** MesDBA at 455 nm, MesDBP at **(b)** 418 nm and **(c)** 510 nm, MesDBPI at **(d)** 480 and **(e)** 550 nm, and MesDBPI-d_{18} at **(f)** 480 and **(g)** 550 nm in 0.1, 1, 10 wt % doped PMMA films at room temperature, respectively.

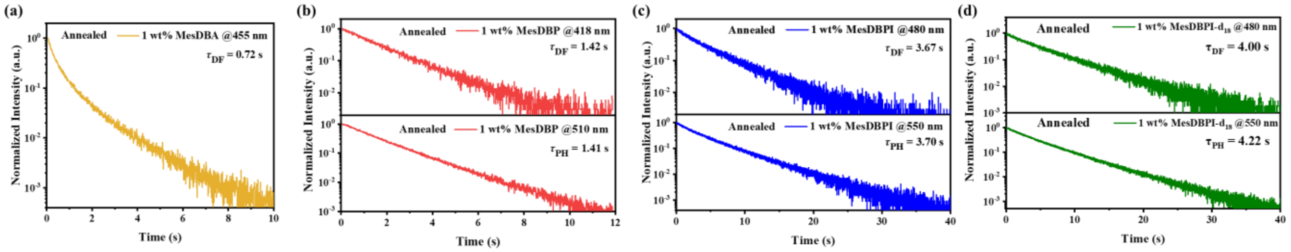

Figure S25. Transient PL decay curves of **(a)** MesDBA at 455 nm, **(b)** MesDBP at 418 nm and 510 nm, **(c)** MesDBPI at 480 and 550 nm and **(d)** MesDBPI-d_{18} at 480 and 550 nm in annealed 1 wt % doped PMMA films, respectively.

The relative rate constants could be estimated using the following equation:[2]

$$k_{\mathrm{PF}} = 1/\tau_{\mathrm{PF}} \tag{S1}$$

$$k_{\mathrm{DF}} = 1/\tau_{\mathrm{DF}} \tag{S2}$$

$$k_{\mathrm{r,S}} = \Phi_{\mathrm{PF}} k_{\mathrm{PF}} \tag{S3}$$

$$\Phi_{\mathrm{DE}} = \Phi_{\mathrm{DF}} + \Phi_{\mathrm{Ph}} \tag{S4}$$

$$R_{\mathrm{DE}}^{\mathrm{DF}} = 1 - \frac{\Phi_{\mathrm{Ph}}}{\Phi_{\mathrm{Ph}} + \Phi_{\mathrm{DF}}} \tag{S5}$$

$$k_{\mathrm{ISC}} = k_{\mathrm{PF}} \Phi_{\mathrm{ISC}} \tag{S6}$$

$$k_{\mathrm{RISC}} = \frac{k_{\mathrm{PF}} k_{\mathrm{DF}} \Phi_{\mathrm{DF}}}{k_{\mathrm{ISC}} \Phi_{\mathrm{PF}}} \tag{S7}$$

$$k_{\mathrm{r,T}} = k_{\mathrm{DF}} \frac{\Phi_{\mathrm{Ph}}}{\Phi_{\mathrm{ISC}}} \tag{S8}$$

$$k_{\mathrm{nr,T}} = k_{\mathrm{DF}} - (1 - \Phi_{\mathrm{ISC}}) k_{\mathrm{RISC}} - k_{\mathrm{r,T}} \tag{S9}$$

Assuming that $k_{\mathrm{nr,S}} \approx 0$,

$$\Phi_{\mathrm{ISC}} = \frac{k_{\mathrm{ISC}}}{k_{\mathrm{r,S}} + k_{\mathrm{nr,S}} + k_{\mathrm{ISC}}} \approx 1 - \Phi_{\mathrm{PF}} \tag{S10}$$

$$k_{\mathrm{nr,T}} = k_{\mathrm{DF}} - (1 - \Phi_{\mathrm{ISC}}) k_{\mathrm{RISC}} - k_{\mathrm{r,T}} \tag{S11}$$

Assuming that $k_{\mathrm{nr,T}} \approx 0$,

$$\Phi_{\mathrm{ISC}} = \frac{\Phi_{\mathrm{DE}} - \Phi_{\mathrm{Ph}}(1 - \Phi_{\mathrm{PF}})}{\Phi_{\mathrm{DE}} + \Phi_{\mathrm{PF}} - \Phi_{\mathrm{Ph}}} \tag{S12}$$

$$k_{\mathrm{nr,S}} = k_{\mathrm{PF}} - k_{\mathrm{ISC}} - k_{\mathrm{r,S}} \tag{S13}$$

Where k_{PF} and k_{DF} represent the decay rate constants for prompt and delayed fluorescence, respectively. τ_{PF}, τ_{DF}, and τ_{Ph} are prompt, delayed fluorescence and phosphorescence decay time, respectively, experimentally

determined from transient PL characteristics. Φ_{PF}, Φ_{DF}, and Φ_{Ph} indicate prompt fluorescence, delayed fluorescence, and phosphorescence components of total Φ_{PL}, respectively. Moreover, $k_{r,S}$, $k_{nr,S}$, k_{ISC}, k_{RISC}, $k_{r,T}$, and $k_{nr,T}$ represent the radiative decay rate of the S_1 state, the nonradiative decay rate of the S_1 state, intersystem crossing (ISC) rate, reverse intersystem crossing (RISC) rate, the radiative decay rate of the T_1 state, the nonradiative decay rate of the T_1 state.

Table S3. Quantum yield and decay lifetime of three compounds doped in PMMA with different concentrations

wt % in PMMA	λ_{max}[a] [nm]	Φ_{PL}[b] [%]	Φ_{PF}[c] [%]	Φ_{DF}[c] [%]	Φ_{Ph}[d] [%]	τ_{PF}[e] [ns]	τ_{DF}[e] [s]	τ_{Ph}[e] [s]
				MesDBA				
0.1	460	6.7	0.3	6.4	-	3.24	0.58	-
10	460	40.6	0.9	39.7	-	4.83	0.16	-
				MesDBP				
0.1	414	62.4	55.1	1.1	6.2	4.79	1.54	1.10
10	440	29.1	21.6	1.5	6.0	3.90	1.21	0.93
				MesDBPI				
0.1	482	13.6	10.2	0.2	3.2	40.86	2.02	2.43
10	482	28.2	13.6	1.0	13.6	42.56	1.47	2.23
				MesDBPI-d_{18}				
0.1	482	21.6	13.6	0.6	7.4	52.13	2.75	2.78
10	482	29.1	16.1	1.6	11.4	58.15	2.18	2.35

[a]PL peak. [b]Absolute PLQY (Φ_{PL}). [c]The prompt (Φ_{PF}) and delayed (Φ_{DF}) fluorescent component of PLQY. [d] The phosphorescent component of PLQY [e]Lifetime of the prompt (τ_{PF}), delayed fluorescent component (τ_{DF}), and phosphoresce(τ_{Ph}) as determined from the transient PL.

Table S4. Relative rate constants of three compounds doped in different concentrations PMMA matrix

Emitters	wt %	k_{PF}[a] (10^7 s^{-1})	k_{DF}[a] (s^{-1})	$k_{r,S}$[b] (10^7 s^{-1})	$k_{nr,S}$[b] (10^7 s^{-1})	k_{ISC}[c] (10^7 s^{-1})	k_{RISC}[d] (s^{-1})	$k_{r,T}$[e] (s^{-1})	$k_{nr,T}$[e] (s^{-1})
MesDBA	0.1	30.9	2.08	0.09	1.29	29.5	44.6	-	1.95
	1[f]	21.2 (20.1)	1.72 (1.39)	0.11 (0.32)	0.42 (0.25)	20.7 (19.5)	67.9 (48.3)	-	1.38 (0.62)
	10	20.7	6.25	0.19	0.27	20.2	278.2	-	3.75
MesDBP	0.1	20.9	0.65	11.50	7.70	1.68	0.03	0.09	0.54
	1	22.5 (21.9)	0.79 (0.70)	10.54 (8.60)	9.52 (11.73)	2.41 (1.55)	0.05 (0.02)	0.11 (0.06)	0.66 (0.63)
	10	25.6	0.83	5.54	17.0	3.10	0.07	0.06	0.75
MesDBPI	0.1	2.45	0.50	0.25	2.07	0.12	0.01	0.02	0.48
	1	2.39 (2.15)	0.50 (0.27)	0.43 (0.34)	1.58 (1.53)	0.39 (0.28)	0.03 (0.02)	0.07 (0.03)	0.42 (0.24)
	10	2.35	0.68	0.32	1.57	0.46	0.06	0.11	0.57
MesDBPI-d_{18}	0.1	1.92	0.36	0.26	1.44	0.22	0.02	0.03	0.33
	1	1.92 (1.69)	0.39 (0.25)	0.35 (0.29)	1.23 (1.17)	0.33 (0.23)	0.03 (0.01)	0.06 (0.03)	0.33 (0.22)
	10	1.72	0.46	0.28	1.11	0.33	0.05	0.06	0.39

[a]The rate constant of prompt (k_{PF}) and delayed (k_{DF}) fluorescent component. [b]The rate constant of the radiative ($k_{r,S}$) and the non-radiative singlet decay ($k_{nr,S}$). [c]The rate constant of intersystem crossing (k_{ISC}). [d]The rate constant of reverse intersystem crossing (k_{RISC}). [e]The rate constant of the radiative ($k_{r,T}$) and the non-radiative triplet decay ($k_{nr,T}$). [f]The values in parentheses represent the experimental results obtained after annealing.

Table S5. Summary of various afterglow systems reported with second-scale lifetimes

TADF-type afterglow (lifetime > 0.1 s)				
Dopant	Host/Matrix	Φ_{DF} [%][a]	τ_{DF} [s][b]	Reference
MesDBA	PMMA	54.8	0.72	*This work*
$DPOMeBF_2$	PhB	68.7	0.309	*Angew. Chem. Int. Ed.* **2021**, *60*, 17138.
$6\text{-}BF_2bdk$	PhB	83.0	0.433	*Sci. China: Chem.* **2023**, *66*, 1120.
Pyrylium salts	PhB	48.0	0.225	*Chem. Eng. J.* **2022**, *431*, 134197.
$CorBF_2$	MeOBP	42.5	0.471	*J. Mater. Chem. C* **2023**, *11*, 2291.
$Me2TPABF_2$	MeOBP	42.8	0.375	*Adv. Funct. Mater.* **2021**, *32*, 2110207.
3-PhB	PhB	64.7	0.199	*Adv. Opt. Mater.* **2023**, *12*, 2301619.
$N\beta NBF_2$	MeOBP	40.1	0.250	*J. Mater. Chem. C* **2022**, *10*, 4795.
TBuDTPPAO	SF	8.4	0.298	*Nat. Commun.* **2024**, *15*, 1596.
PEF	BA	75.7	0.271	*Anal. Chem.* **2024**, *96*, 5640.
4- MeOBP	MeOBP	77.7	0.171	*Adv. Opt. Mater.* **2025**, *13,* 2403551.

RTP-type or hybrid-type afterglow (lifetime > 1 s)				
Dopant	Host/Matrix	Φ_{Ph} [%][c]	τ_{Ph} [s][d]	Reference
MesDBP	PMMA	5.2	1.41	*This work*
MesDBPI	PMMA	8.7	3.70	*This work*
$MesDBPI\text{-}d_{18}$	PMMA	10.0	4.22	*This work*
phenDBA	PMMA	15	3.2	*Angew. Chem. Int. Ed.* **2023**, 62, e202215071.
1,2-OPh-PLA	Main chain polymer	3.9	1.20	*Angew. Chem. Int. Ed.* **2016**, *55*, 9872.
IPA	LDH	3.0	1.23	*Chem. Sci.* **2017**, *8*, 590.
Phenanthrene	β-CD-Chloroform	-	2.30	*Russ. Chem. Bull.* **1996**, *45*, 969.
Phenanthrene	β-CD	-	2.40	*Russ. Chem. Bull.* **1997**, *46*, 1386.
Naphthalene	β-CD	-	1.78	*Russ. Chem. Bull.* **1997**, *46*, 1386.
$Naphthalene\text{-}d_8$	β-CD	-	10.80	*Russ. Chem. Bull.* **1997**, *46*, 1386.
Phenanthrene	β-CD-Cyclohexane	-	3.00	*Russ. Chem. Bull.* **1997**, *46*, 1386.
Naphthalene	β-CD-Cyclohexane	-	14.20	*Russ. Chem. Bull.* **1997**, *46*, 1386.
$Naphthalene\text{-}d_8$	β-CD-Adamantane	-	15.50	*Russ. Chem. Bull. Int. Edit.* **2003**, *52*, 916.
$Naphthalene\text{-}d_8$	β-CD-Diadamantyl	-	11.90	*Russ. Chem. Bull. Int. Edit.* **2005**, *54*, 2752.
GE9	Cholesterol + THEB	8.0	1.22	*Adv. Funct. Mater.* **2013**, *23*, 3386.
GE9	β-Estradiol	9.4	1.67	*Adv. Funct. Mater.* **2013**, *23*, 3386.

GE10	β-Estradiol	7.7	3.75	*Adv. Funct. Mater.* **2013**, *23*, 3386.
GE17	β-Estradiol	6.0	1.04	*Adv. Funct. Mater.* **2013**, *23*, 3386.
GE18	β-Estradiol	12.1	1.60	*Adv. Funct. Mater.* **2013**, *23*, 3386.
GE19	β-Estradiol	4.4	1.39	*Adv. Funct. Mater.* **2013**, *23*, 3386.
GE20	β-Estradiol	7.8	1.74	*Adv. Funct. Mater.* **2013**, *23*, 3386.
GE21	β-Estradiol	12.9	3.98	*Adv. Funct. Mater.* **2013**, *23*, 3386.
GE22	β-Estradiol	13.1	1.74	*Adv. Funct. Mater.* **2013**, *23*, 3386.
GE23	β-Estradiol	6.0	1.30	*Adv. Funct. Mater.* **2013**, *23*, 3386.
GE24	β-Estradiol	10.0	4.04	*Adv. Funct. Mater.* **2013**, *23*, 3386.
GE25	β-Estradiol	9.8	2.16	*Adv. Funct. Mater.* **2013**, *23*, 3386.
GE26	β-Estradiol	4.3	2.72	*Adv. Funct. Mater.* **2013**, *23*, 3386.
GE27	β-Estradiol	10.6	2.39	*Adv. Funct. Mater.* **2013**, *23*, 3386.
GE30	β-Estradiol	6.2	1.36	*Chem. Phys. Lett.* **2014**, *591*, 119.
GE21	Cholesterol + THEB	3.1	1.40	*Adv. Opt. Mater.* **2013**, *1*, 483.
6EhHBC	β-Estradiol	2.6	3.90	*Adv. Opt. Mater.* **2017**, *5*, 1600996.
N-CNDs	Polyurethane	7.0	1.06	*Chem. Mater.* **2016**, *28*, 8221.
CPBA	BA	-	1.97	*Adv. Opt. Mater.* **2023**, *11*, 2300207.
PCz	DTT	16.2	1.08	*J. Am. Chem. Soc.* **2023**, *145*, 13392.
Ch1	β-Estradiol	-	1.00	*ACS Mater. Lett.* **2023**, *5*, 1649.
p-Bph-BOH	β-CD	3.3	1.03	*ACS Nano*, **2023**, *17*, 12895.
DPAF	β-Estradiol	6.2	1.19	*Adv. Sci.* **2023**, *10*, 2304374.
Carboline 3	CB7	3.0	1.20	*Small*, **2024**, *20*, 2307318.
Carboline 3	CB7+β-CD	7.0	2.10	*Small*, **2024**, *20*, 2307318.
TMA	Cyanuric acid	9.3	1.13	*Adv. Opt. Mater.* **2024**, *12*, 2301937.
TMA, water	Cyanuric acid	46.1	1.67	*Adv. Opt. Mater.* **2024**, *12*, 2301937.
H1	DTT	-	1.08	*Small*, **2024**, *20*, 2310226.
TTCA	CA	1.8	2.86	*Adv. Opt. Mater.* **2024**, *12*, 2401270.
TTCA	CA:MA=3:1	7.3	3.21	*Adv. Opt. Mater.* **2024**, *12*, 2401270.
TTCA	CA:MA=1:1	5.6	3.16	*Adv. Opt. Mater.* **2024**, *12*, 2401270.
TTCA	CA:MA=1:3	4.7	2.92	*Adv. Opt. Mater.* **2024**, *12*, 2401270.
TTCA	CA:BU=3:1	3.7	3.30	*Adv. Opt. Mater.* **2024**, *12*, 2401270.
TTCA	CA:BU=1:1	5.2	3.36	*Adv. Opt. Mater.* **2024**, *12*, 2401270.

TTCA	CA:BU=1:3	3.9	3.19	*Adv. Opt. Mater.* **2024**, *12*, 2401270.
TTCA	BU	2.6	2.88	*Adv. Opt. Mater.* **2024**, *12*, 2401270.
TTCA	CA:UA=3:1	3.5	3.32	*Adv. Opt. Mater.* **2024**, *12*, 2401270.
TTCA	CA:UA=1:1	3.3	3.16	*Adv. Opt. Mater.* **2024**, *12*, 2401270.
TTCA	CA:UA=1:3	2.1	2.80	*Adv. Opt. Mater.* **2024**, *12*, 2401270.
MHA	BA	-	1.85	*Adv. Sci.* **2023**, *10*, 2300139.
PHA	BA	-	2.01	*Adv. Sci.* **2023**, *10*, 2300139.
PAC1	BA	8.5	1.71	*J. Am. Chem. Soc.* **2024**, *146*, 25211.
PAC2	BA	10.9	1.99	*J. Am. Chem. Soc.* **2024**, *146*, 25211.
PAC4	BA	15.9	2.21	*J. Am. Chem. Soc.* **2024**, *146*, 25211.
PAC5	BA	10.4	1.13	*J. Am. Chem. Soc.* **2024**, *146*, 25211.
PAC7	BA	7.3	2.32	*J. Am. Chem. Soc.* **2024**, *146*, 25211.
PAC11	BA	14.2	1.00	*J. Am. Chem. Soc.* **2024**, *146*, 25211.
PAC12	BA	12.0	1.05	*J. Am. Chem. Soc.* **2024**, *146*, 25211.
BCz	Cz	2.0	1.04	*Angew. Chem. Int. Ed.* **2023**, *62*, e202310335.
BTDA-H	TCN-H	10.7	1.03	*Chem. Eng. J.* **2024**, *493*, 152492.
IaCzA	PVA	19.8	1.81	*Angew. Chem. Int. Ed.* **2022**, *61*, e202201820.
IbCzA	PVA	8.2	1.72	*Angew. Chem. Int. Ed.* **2022**, *61*, e202201820.
5, 7-ICz	PVA	5.5	1.50	*Adv. Funct. Mater.* **2023**, *33*, 2208895.
5, 12-ICz	PVA	10.8	1.54	*Adv. Funct. Mater.* **2023**, *33*, 2208895.
11, 12-ICz	PVA	44.1	2.04	*Adv. Funct. Mater.* **2023**, *33*, 2208895.
PCz	DTT	16.2	1.08	*J. Am. Chem. Soc.* **2023**, *145*, 13392.
DPCz	PVA	0.8	2.04	*J. Am. Chem. Soc.* **2021**, *143*, 13675.
DPP-BOH	PVA	7.5	2.43	*Nat. Commun.*, **2022**, *13*, 347.
CzA	PVA	20.1	2.76	*Nat. Commun.*, **2022,** *13*, 4890
m-Bp-BOH	PVA	5.4	2.43	*Sci. Adv.*, **2022**, *8*, eabl8392.
p-Bp-BOH	PVA	12.3	2.34	*Sci. Adv.*, **2022**, *8*, eabl8392.
Nap-BOH	PVA	5.7	1.48	*Sci. Adv.*, **2022**, *8*, eabl8392.
BNap-BOH	PVA	5.8	1.33	*Sci. Adv.*, **2022**, *8*, eabl8392.
910DAPT	UF	11.85	3.326	*Nat. Commun.*, **2024**, *15*, 4415.

Crystal-type afterglow (lifetime > 1 s)	Φ_{Ph} [%][c]	τ_{Ph} [s][d]	Reference

DECzT	0.6	1.35	*Nat. Mater.* **2015**, *14*, 685.
DPhCzT	1.3	1.06	*Nat. Mater.* **2015**, *14*, 685.
PB	-	1.20	*ChemPhotoChem* **2017**, *1*, 102.
PDBEG	-	1.60	*ChemPhotoChem* **2017**, *1*, 102.
PBd2	-	1.10	*ChemPhotoChem* **2017**, *1*, 102.
PBd5	-	1.30	*ChemPhotoChem* **2017**, *1*, 102.
p-F-PBEG	-	1.70	*ChemPhotoChem* **2017**, *1*, 102.
PDBPr	-	2.10	*ChemPhotoChem* **2017**, *1*, 102.
PDBBu	-	1.60	*ChemPhotoChem* **2017**, *1*, 102.
CE-26	2.0	1.85	*J. Am. Chem. Soc.* **2017**, *139*, 2728.
CE-27	-	1.79	*J. Am. Chem. Soc.* **2017**, *139*, 2728.
CE-29	-	1.73	*J. Am. Chem. Soc.* **2017**, *139*, 2728.
CE-30	-	1.57	*J. Am. Chem. Soc.* **2017**, *139*, 2728.
CE-35	-	1.39	*J. Am. Chem. Soc.* **2017**, *139*, 2728.
Zn-IPA	3.4	1.32	*Adv. Opt. Mater.* **2016**, *4*, 897.

Coronene system (lifetime > 1 s)

Dopant	Host/Matrix	Φ_{Ph} [%][c]	τ_{Ph} [s][d]	Reference
Coronene	PMMA	4	6.0	*J. Phys. Chem.* **1967**, *71*, 4499.
Coronene-d_{12}	PMMA	12	23.0	*J. Phys. Chem.* **1967**, *71*, 4499.
Coronene	ZIF-8	1.8	7.4	*Adv. Opt. Mater.* **2016**, *4*, 1015.
Coronene-d_{12}	ZIF-8	3.7	22.4	*Adv. Opt. Mater.* **2016**, *4*, 1015.
Coronene-d_{12}	β-Estradiol	18.0	17.0	*Adv. Opt. Mater.* **2017**, *5*, 1600996.
Coronene-d_{12}	CzSte	5.3	4.7	*Adv. Mater.* **2016**, *28*, 655.
2	β-Estradiol	11	3.1	*JACS Au* **2021**, *1*, 945.
3	β-Estradiol	19	1.8	*JACS Au* **2021**, *1*, 945.
2D	β-Estradiol	16	3.8	*JACS Au* **2021**, *1*, 945.
3D	β-Estradiol	35	2.2	*JACS Au* **2021**, *1*, 945.
Coronene-d_{12}	PMMA emulsion	-	7.66	*Chem. Commun.* **2023**, *59*, 10500.
Coronene	MF resin	22.7	4.83	*Angew. Chem.* **2024**, 63, e202318516.
CoDe	PhB	38	1.5	*Angew. Chem. Int. Ed.* **2025**, e202513685

[a]Quantum yield of delayed fluorescence component. [b]Lifetime of delayed fluorescence component. [c]Quantum yield of phosphorescence component. [d]Lifetime of phosphorescence component.

Table S6. NIR-TAS decay lifetimes obtained at various concentrations of MesDBPI across different integrated spectral regions

[MesDBPI][a] (mM)	$\tau_{1,B1}$[b] [μs]	$\tau_{2,B1}$[b] [μs]	$\tau_{1,B2}$[c] [μs]	$\tau_{2,B2}$[c] [μs]
1.00	18.1	116.6	19.0	129.0
0.75	23.8	147.3	24.3	154.2
0.50	24.7	174.0	23.1	179.5

[a]MesDBPI in THF solution. [b]Integration range of 6000-6500 cm^{-1}. [c]Integration range of 6800-7300 cm^{-1}.

6. Computational methods

In this work, the electronic structures of two emitters were calculated with Gaussian 16[3] unless specified otherwise. The ground state (S_0) geometries were optimized using the density functional theory (DFT). The time-dependent density functional theory (TD-DFT) or Tamm-Dancoff approximated density functional theory (TDA-DFT) were used to optimize the geometries of the first excited singlet state (S_1) and the triplet excited states (T_1, T_2, and T_3).[4] We utilized the polarizable continuum model (PCM)[5] and the two-layer ONIOM model[6] of the quantum mechanics/molecular mechanics (QM/MM) method, respectively, to simulate basic photophysical data in toluene and the solid phase. The innermost molecule was found to be the higher layer by the accurate high-level QM method, and the outermost molecules were treated as the lower layer using the efficient universal force field (UFF) method.[7] In the QM/MM geometry optimization process, only the atoms in the QM part can move and others in the MM part were frozen. For after annealing simulation, all atoms in the QM and MM parts can move. The initial geometries of MesDBA, MesDBP, MesDBPI, and MesDBPI-d_{18} in the solid phase were based on the measured crystalline structures.

Several functionals with different percentages of Hartree-Fock exchange (HF%), including B3LYP (20%), PBE0 (25%), BMK (42%), M06-2X (54%), CAM-B3LYP (19% HF at short range and 65% HF at long range), and ωB97XD (22.2% HF at short range and 100% HF at long range) are tested.[4, 8, 9] It should be noted that most of the experimental ΔE_{ST} values are deduced from fluorescence and phosphorescence spectra.[4] The computational results of the vertical absorption energy (ΔE_{VA}), vertical emission energy (ΔE_{VE}), adiabatic energy (ΔE_{AD}), and the energy gap (ΔE_{ST}) were calculated. Absorption and emission spectra were simulated by TD-DFT based on vertical energies at the stable geometry structures. It was found that the emission wavelength changes significantly when functionals were different, and the best approach for calculating emission peak ($\lambda_{Fl,max}$ and $\lambda_{Ph,max}$), the first adiabatic singlet and triplet excited energies and ΔE_{ST} was BMK/6-31G (d). The spin-orbit coupling (SOC) matrix between S_1 and T_n (n =1-3) states were calculated with the optimized geometries of S_1 by the ORCA program, respectively.[10] And the transition dipole moments between T_1 and S_0 states were calculated with the optimized geometries of T_1 by the Dalton package.[11]

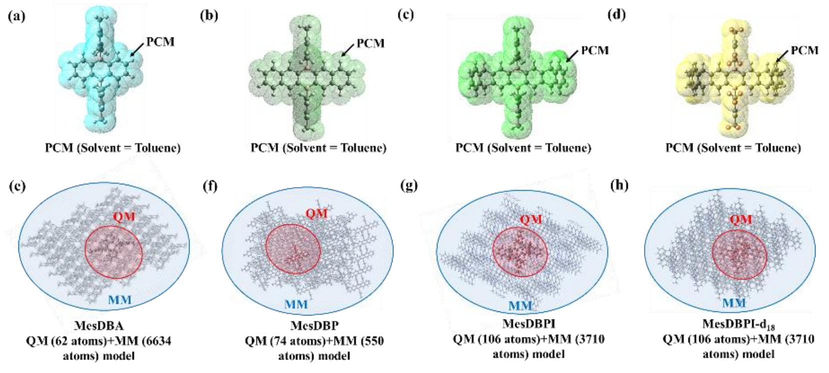

Figure S26. (a) PCM model(toluene) and **(b)** ONIOM model of MesDBA, MesDBP, MesDBPI, and MesDBPI-d_{18}. In ONIOM model, single DBA molecule is treated as the high layer (QM), respectively, and surrounding molecules are regarded as the low layer (MM).

Table S7. Calculated excited-state characteristics of MesDBA, MesDBP, MesDBPI, and MesDBPI-d$_{18}$ in toluene and the solid phase.

Molecules	Method	$\lambda_{Fl,max}$[a] [nm]	$\lambda_{Ph,max}$[b] [nm]	S_1[c] [eV]	T_1[d] [eV]	ΔE_{ST}[e] [eV]	HOMO[f] [eV]	LUMO[f] [eV]
				Toluene				
MesDBA	B3LYP	544	594	2.51	2.35	0.16	-5.95	-2.69
	CAM-B3LYP	392	605	3.36	2.37	0.99	-7.36	-1.49
	PBE0	511	604	2.66	2.32	0.33	-6.23	-2.64
	BMK	453	527	2.97	2.65	0.32	-6.55	-2.18
	M06-2X	394	503	3.32	2.78	0.53	-7.35	-1.84
	ωB97X-D	413	560	3.43	2.53	0.90	-7.96	-0.96
MesDBP	B3LYP	489	548	2.76	2.49	0.27	-5.87	-2.40
	CAM-B3LYP	361	556	3.54	2.63	0.91	-7.15	-1.27
	PBE0	463	550	2.90	2.51	0.40	-6.15	-2.35
	BMK	417	521	3.21	2.69	0.52	-6.63	-1.91
	M06-2X	360	493	3.57	2.87	0.70	-7.16	-1.65
	ωB97X-D	354	523	3.62	2.70	0.92	-7.75	-0.72
MesDBPI (MesDBPI-d$_{18}$)	B3LYP	534	688	2.55	2.04	0.51	-5.83	-2.62
	CAM-B3LYP	440	687	2.83	1.88	0.95	-7.16	-1.58
	PBE0	502	696	2.69	2.03	0.66	-6.11	-2.57
	BMK	476	600	2.85	2.34	0.51	-6.47	-2.23
	M06-2X	439	565	3.14	2.49	0.65	-7.17	-1.92
	ωB97X-D	427	630	3.18	2.28	0.90	-7.76	-1.00
				Solid				
MesDBA	B3LYP	554	594	2.49	2.36	0.13	-5.88	-2.44
	CAM-B3LYP	396	604	3.35	2.37	0.98	-7.30	-1.25
	PBE0	518	605	2.64	2.33	0.31	-6.15	-2.37
	BMK	458	529	2.96	2.67	0.29	-6.70	-1.90
	M06-2X	398	506	3.32	2.80	0.52	-7.27	-1.59
	ωB97X-D	411	560	3.44	2.54	0.90	-7.88	-0.65
MesDBP	B3LYP	497	556	2.74	2.49	0.25	-5.75	-2.13
	CAM-B3LYP	375	541	3.56	2.63	0.93	-7.04	-1.03
	PBE0	469	557	2.88	2.50	0.38	-6.05	-2.09
	BMK	422	526	3.19	2.69	0.50	-6.56	-1.63
	M06-2X	379	495	3.52	2.87	0.65	-7.05	-1.39
	ωB97X-D	348	528	3.67	2.70	0.97	-7.62	-0.45
MesDBPI	B3LYP	536	686	2.54	2.04	0.49	-5.75	-2.39
	CAM-B3LYP	439	516	3.09	2.40	0.69	-7.08	-1.23
	PBE0	489	693	2.72	2.04	0.69	-6.01	-2.32
	BMK	473	598	2.86	2.34	0.52	-6.52	-1.88
	M06-2X	430	563	3.17	2.50	0.66	-7.08	-1.58
	ωB97X-D	425	629	3.19	2.28	0.91	-7.67	-0.63
MesDBPI-d$_{18}$	B3LYP	536	634	2.54	2.17	0.37	-5.68	-2.26
	CAM-B3LYP	439	575	3.09	2.43	0.66	-7.00	-1.15
	PBE0	503	619	2.69	2.23	0.46	-5.95	-2.22
	BMK	473	598	2.86	2.34	0.52	-6.49	-1.76
	M06-2X	430	529	3.16	2.62	0.54	-7.01	-1.51
	ωB97X-D	425	550	3.19	2.54	0.66	-7.58	-0.54

[a]Fluorescence peak. [b]Phosphorescence peak. [c]The adiabatic singlet excited state energy. [d]The adiabatic triplet excited state energy. [e]The adiabatic singlet-triplet splitting energy (ΔE_{ST}). [f]The energy level of HOMO and LUMO.

Table S8. Calculated vertical absorption energies of MesDBA, MesDBP, MesDBPI, and MesDBPI-d$_{18}$ in toluene and the solid phase.

Molecules	Method	S_1[a] [eV]	T_1[a] [eV]	T_2[a] [eV]	T_3[a] [eV]	ΔE_{S1T1}[b] [eV]	ΔE_{S1T2}[b] [eV]	ΔE_{S1T3}[b] [eV]
Toluene								
MesDBA	B3LYP	2.69	2.60	2.62	2.77	0.09	0.07	-0.09
	CAM-B3LYP	3.49	2.64	3.23	3.34	0.85	0.26	0.15
	PBE0	2.82	2.57	2.73	2.92	0.25	0.09	-0.11
	BMK	2.77	2.53	2.70	3.02	0.24	0.07	-0.25
	M06-2X	3.44	3.08	3.33	3.62	0.36	0.10	-0.19
	ωB97X-D	3.61	2.80	3.38	3.45	0.80	0.23	0.16
MesDBP	B3LYP	2.94	2.41	2.60	2.65	0.53	0.35	0.29
	CAM-B3LYP	3.65	2.33	2.56	2.99	1.32	1.09	0.66
	PBE0	3.07	2.32	2.55	2.66	0.76	0.52	0.41
	BMK	3.39	2.61	2.86	2.87	0.78	0.53	0.52
	M06-2X	3.68	2.91	3.09	3.14	0.77	0.59	0.55
	ωB97X-D	3.73	2.49	2.70	3.09	1.23	1.03	0.64
MesDBPI (MesDBPI-d$_{18}$)	B3LYP	2.72	2.25	2.65	2.80	0.46	0.07	-0.09
	CAM-B3LYP	3.33	2.41	3.04	3.37	0.92	0.29	-0.03
	PBE0	2.85	2.26	2.76	2.86	0.59	0.09	-0.01
	BMK	3.09	2.45	3.05	3.14	0.64	0.03	-0.05
	M06-2X	3.40	2.77	3.34	3.36	0.63	0.06	0.03
	ωB97X-D	3.44	2.57	3.18	3.47	0.88	0.27	-0.03
Solid								
MesDBA	B3LYP	2.70	2.60	2.63	2.79	0.10	0.07	-0.09
	CAM-B3LYP	3.50	2.65	3.23	3.35	0.86	0.28	0.16
	PBE0	2.83	2.57	2.74	2.94	0.26	0.10	-0.11
	BMK	3.15	2.76	3.05	3.28	0.38	0.10	-0.14
	M06-2X	3.46	3.09	3.35	3.64	0.36	0.10	-0.18
	ωB97X-D	3.62	2.81	3.38	3.45	0.81	0.24	0.17
MesDBP	B3LYP	2.97	2.40	2.60	2.64	0.57	0.37	0.33
	CAM-B3LYP	3.69	3.41	3.46	3.54	0.29	0.23	0.15
	PBE0	3.10	2.31	2.53	2.66	0.80	0.57	0.44
	BMK	3.41	2.60	2.84	2.87	0.81	0.57	0.54
	M06-2X	3.70	2.90	3.08	3.14	0.80	0.62	0.56
	ωB97X-D	3.76	2.49	2.69	3.08	1.27	1.07	0.68
MesDBPI	B3LYP	2.73	2.25	2.65	2.78	0.48	0.08	-0.05
	CAM-B3LYP	3.32	2.40	3.03	3.28	0.92	0.30	0.04
	PBE0	2.86	2.25	2.75	2.84	0.61	0.11	0.02
	BMK	3.07	2.44	3.04	3.11	0.64	0.03	-0.04
	M06-2X	3.40	2.77	3.34	3.36	0.63	0.06	0.03
	ωB97X-D	3.43	2.56	3.16	3.40	0.88	0.27	0.03
MesDBPI-d$_{18}$	B3LYP	2.73	2.25	2.65	2.78	455	0.48	0.08
	CAM-B3LYP	3.32	2.40	3.03	3.28	373	0.92	0.30
	PBE0	2.86	2.25	2.76	2.84	433	0.61	0.10
	BMK	3.07	2.44	3.04	3.11	403	0.64	0.03
	M06-2X	3.39	2.77	3.34	3.36	365	0.63	0.06
	ωB97X-D	3.43	2.55	3.16	3.40	361	0.88	0.27

[a]The vertical absorption singlet and triplet energies. [b]The vertical absorption ΔE_{ST}.

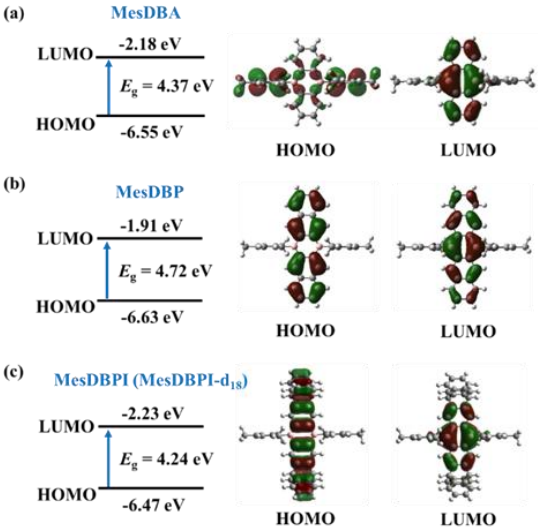

Figure S27. Frontier molecular orbitals (FMOs) of **(a)** MesDBA, **(b)** MesDBP, and **(c)** MesDBPI (MesDBPI-d_{18}) in toluene at BMK/6-31G(d).

To understand the contributions of RISC rate from T_1, T_2, and T_3 states to S_1, we estimated the quantitative contributions of each pathway. The effective RISC rate ($k_{\mathrm{RISC}}^{\mathrm{eff}}$) can be rewritten as:

$$k_{\mathrm{RISC}}^{\mathrm{eff}} = \frac{P_1 k_{\mathrm{T_1-S_1}}^2}{k_{\mathrm{T_1-S_1}} + k_{\mathrm{T_2-S_1}} + k_{\mathrm{T_3-S_1}}} + \frac{P_2 k_{\mathrm{T_2-S_1}}^2}{k_{\mathrm{T_1-S_1}} + k_{\mathrm{T_2-S_1}} + k_{\mathrm{T_3-S_1}}} + \frac{P_3 k_{\mathrm{T_3-S_1}}^2}{k_{\mathrm{T_1-S_1}} + k_{\mathrm{T_2-S_1}} + k_{\mathrm{T_3-S_1}}} \quad \text{(S14)}$$

$$k_{\mathrm{RISC}}^{\mathrm{eff}} = k_{\mathrm{T_1-S_1}}^{\mathrm{eff}} + k_{\mathrm{T_2-S_1}}^{\mathrm{eff}} + k_{\mathrm{T_3-S_1}}^{\mathrm{eff}} \quad \text{(S15)}$$

In Eq.(S15), $k_{\mathrm{T_1-S_1}}^{\mathrm{eff}}$, $k_{\mathrm{T_2-S_1}}^{\mathrm{eff}}$, and $k_{\mathrm{T_3-S_1}}^{\mathrm{eff}}$ are the effective RISC rates from T_1, T_2, and T_3 to S_1, respectively. The contributions of T_1, T_2, and T_3 states to RISC are defined as: $A_n = \frac{k_{\mathrm{T_n-S_1}}^{\mathrm{eff}}}{k_{\mathrm{RISC}}^{\mathrm{eff}}}$, n = 1-3, respectively. For three molecules, RISC is predominantly mediated by the T_3 state.

Table S9. The effective RISC rate from T_1, T_2, and T_3 to S_1 and their contributions.

Molecules	$k_{\mathrm{T_1-S_1}}^{\mathrm{eff}}$ [s^{-1}]	$k_{\mathrm{T_2-S_1}}^{\mathrm{eff}}$ [s^{-1}]	$k_{\mathrm{T_3-S_1}}^{\mathrm{eff}}$ [s^{-1}]	A_1 [%]	A_2 [%]	A_3 [%]
MesDBA	1.44×10^{-2}	1.66×10^{0}	8.90×10^{0}	0.1	15.7	84.1
MesDBP	1.32×10^{-5}	7.42×10^{-18}	9.65×10^{-1}	0.0	0.0	100.0
MesDBPI	6.27×10^{-38}	3.38×10^{-10}	1.10×10^{-2}	0.0	0.0	100.0
MesDBPI-d_{18}	9.11×10^{-38}	-	9.63×10^{-3}	0.0	0.0	100.0
MesDBA[a]	5.07×10^{-2}	4.52×10^{0}	7.55×10^{0}	0.4	37.3	62.3
MesDBP[a]	1.31×10^{-9}	3.45×10^{-2}	3.87×10^{-2}	0.0	47.2	52.8
MesDBPI[a]	1.32×10^{-98}	3.15×10^{-6}	3.73×10^{-3}	0.0	0.0	100
MesDBPI-d_{18}[a]	1.32×10^{-111}	3.15×10^{-7}	2.94×10^{-3}	0.0	0.0	100

[a]After annealing simulation.

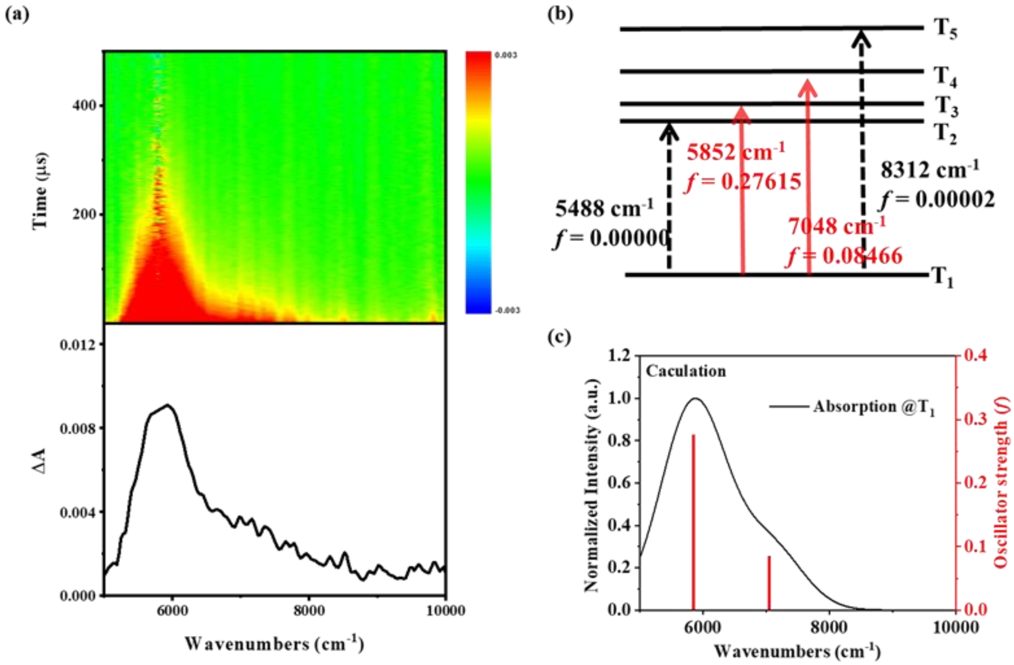

Figure S28. TAS characterization of MesDBPI in THF solution. **(a)** 3D contour plot of differential absorption at 500 µM and the corresponding spectrum at 1.28 µs. **(b)** Calculated vertical absorption energies and oscillator strengths (f) of triplet-triplet transitions in THF. **(c)** Calculated absorption spectra based on vertical excitations from the T_1-optimized geometry in THF.

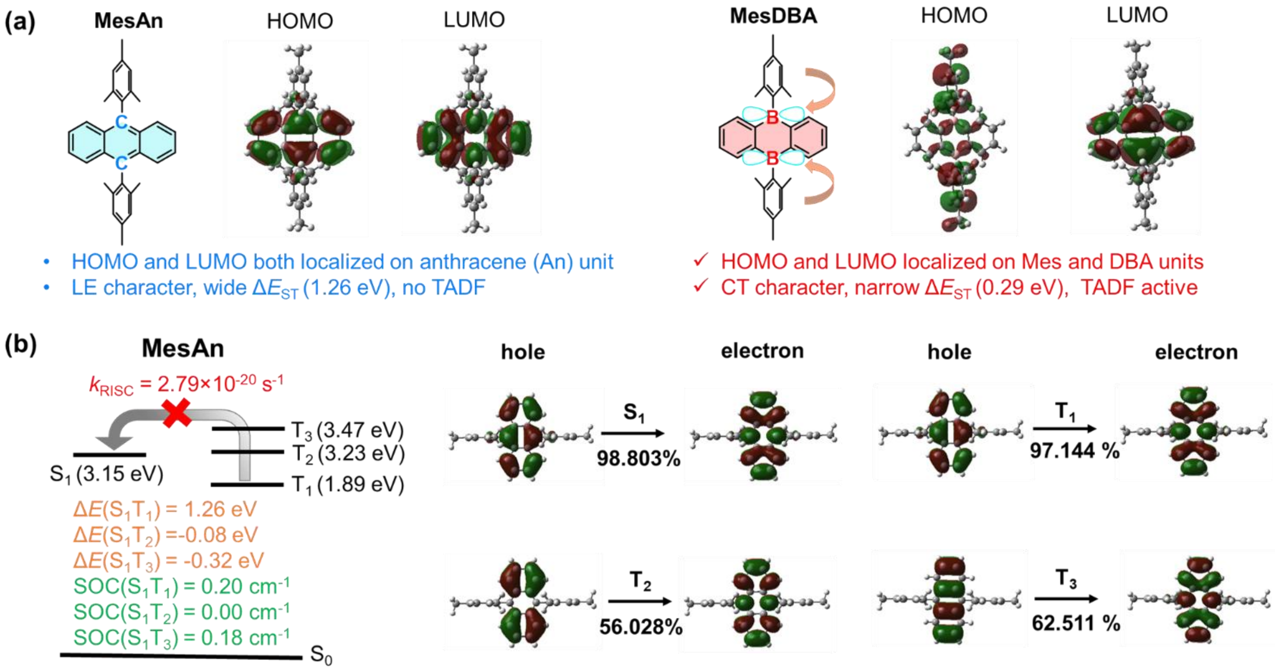

Figure S29. **(a)** Scheme of molecular design and FMOs of MesAn and MesDBA calculated in toluene at the BMK/6-31G(d) level. **(b)** Excited state energy levels, SOC constants, and NTO analyses of the S_1, T_1, T_2, and T_3 states of MesAn calculated in the solid phase at the BMK/6-31G(d) level.

7. OLEDs fabrication and measurement

Organic materials used in device fabrication were purified by sublimation. Devices were fabricated by vacuum deposition onto pre-coated ITO glass with a sheet resistance of 25 Ω/square at a pressure lower than 10^{-6} torr. MoO_3 (1 nm) is utilized as the hole injection layer (HIL), while TAPC (50 nm) functions as the hole transport layer (HTL). mCP (10 nm) serves a dual role as both the HTL and the exciton blocking layer (EBL). 3TPYMB (50 nm) acts as both the electron transport layer (ETL) and the hole blocking layer (HBL). And, LiF (0.5 nm) and Al (100 nm) are employed as the electron injection layer (EIL) and the cathode, respectively. Organic materials were deposited at the rate of 0.5~1.2 Å s^{-1}. LiF and Al were deposited at the rate of 0.1 Å s^{-1} and 3~10 Å s^{-1}, respectively. The rest of the procedures is similar to the reported method. Current-voltage-luminance (I-V-L) characterization and electroluminescent spectra were measured and recorded by using a programmable source meter (2400, Keithley) and a spectroradiometer (CS2000A, Konica Minolta). The Lambertian emission assumption determined external quantum efficiencies and power efficiencies. All devices were encapsulated in a glove box. Then, the EL measurements were performed at room temperature. In the device, 1,1-bis((di-4-tolylamino)phenyl)cyclohexane (TAPC) served as a hole transport layer, N,N-dicarbazoyl-3,5-benzene (mCP) served as an exciton blocking layer, 9-(3-(9H-carbazol-9-yl)phenyl)-9H-carbazole-3-carbonitrile (mCPCN) was used as the host material, and Tris-[3-(3-pyridyl)mesityl]borane (3TPYMB) served as an electron transporter. ITO and Al serve as the anode and cathode, respectively.

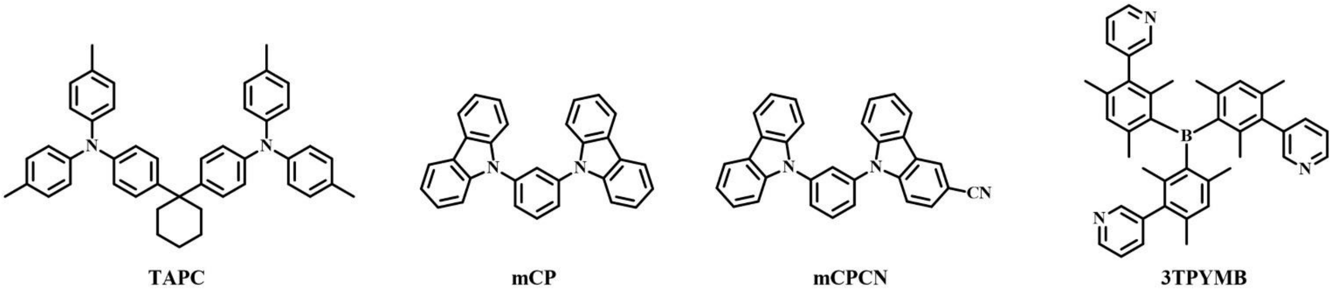

Figure S30. The molecular structures of materials used in the device.

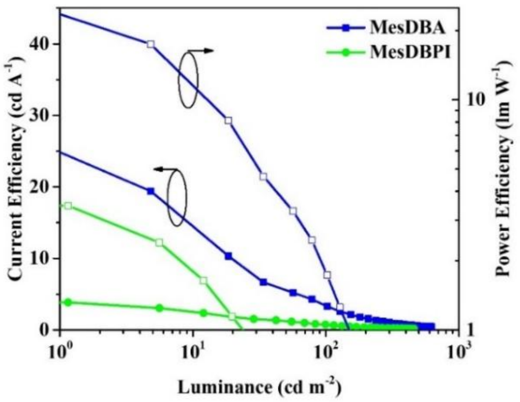

Figure S31. Current efficiency and power efficiency versus luminance characteristics of MesDBA and MesDBPI.

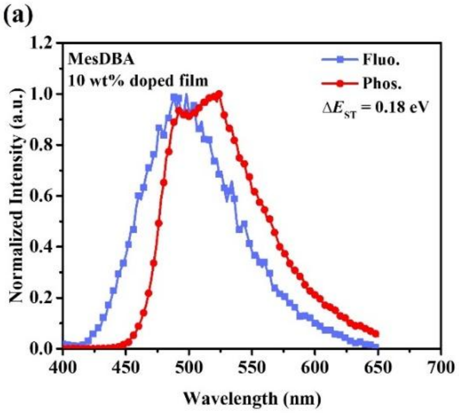

Figure S32. Fluorescence (300 K) and phosphorescence (100 K) spectra of **(a)** MesDBA **(b)** MesDBPI in 10 wt % doped mCPCN films.

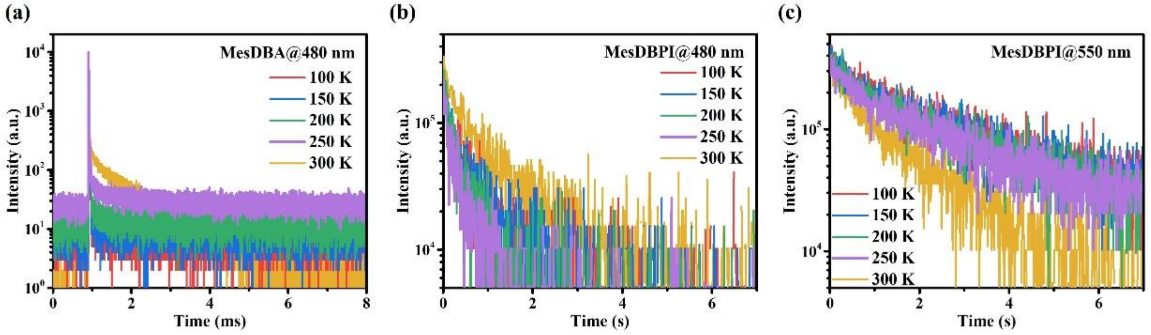

Figure S33. Temperature-dependent transient PL spectra of **(a)** MesDBA at 480 nm, and MesDBPI at **(b)** 480 nm and **(c)** 550 nm in 10 wt % doped mCPCN films, respectively.

Table S10. The summary of electroluminescent properties of afterglow OLEDs

Device	V_d[a] [V]	η_{ext}[b] [%]	η_c[c] [cd/A]	η_p[d] [lm/W]	λ_{max}[e] [nm]	$CIE_{(x,y)}$	τ_{EL}[f] [ms]	Reference
MesDBA	3.0	8.0	19.4	17.4	491	(0.19, 0.37)	11	*This work*
MesDBPI	3.5	1.8	3.9	3.5	488	(0.16, 0.36)	113	*This work*
1% DMFLTPD-d_{36}/CzSte	-	~1.0	-	-	~520	-	390	*Adv. Mater*. **2016**, *28*, 655
1% coronene-d_{12}/CzSte	-	-	-	-	-	-	4300	*Adv. Mater*. **2016**, *28*, 655
1% m-MTDATA/PPT	-	~0.8	-	-	-	-	-	*Adv. Mater.* **2021**, *33*, 2008844
5% NPB/PPT	5.8	1.47	-	-	544	-	356	*Appl. Phys. Rev.* **2022**, *9*, 031410
TPXZPhCor	4.3	3.3	-	-	525	-	-	*Angew. Chem. Int. Ed.* **2023**, *62*, e202309718
BO-NO	2.9	14.7	49.2	53.3	530	(0.33, 0.60)	147	*Matter* **2023**, *6*, 1231

[a]Driving voltage at 1 cd m^{-2}. [b]Maximum external quantum efficiency. [c]Maximum current efficiency. [d]Maximum power efficiency. [e]EL maximum peak. [f]EL lifetime.

8. Calculated optimized geometries

Table S11. Cartesian coordinates of optimized S_0 state of MesDBPI as the QM layer of ONIOM model at BMK/6-31G(d) in the solid phase

Elements	X	Y	Z
B	1.92642200	-2.92198500	-3.00299800
C	1.91157800	-2.58842900	-4.54013800
C	1.79537500	-1.25501900	-5.00740800
C	1.66406300	-0.10500800	-4.01791600
H	1.55715800	0.85479300	-4.53644200
H	2.54335600	-0.03821100	-3.36074200
H	0.78652700	-0.23945900	-3.36994200
C	1.80636300	-0.99012000	-6.38430700
H	1.73158000	0.04053000	-6.72704800
C	1.89529000	-2.01592300	-7.33399600
C	1.88926900	-1.70802600	-8.82185200
H	2.90847900	-1.52829200	-9.19562800
H	1.29492200	-0.81115400	-9.03906300
H	1.46980700	-2.54246900	-9.39798700
C	2.00303200	-3.33018200	-6.86460700
H	2.04835000	-4.15125800	-7.57593500
C	2.03354000	-3.62709300	-5.49512200
C	2.22245300	-5.07169200	-5.05133000
H	3.26226000	-5.25284500	-4.74103200
H	1.98843600	-5.77116100	-5.86379500
H	1.58617700	-5.32063900	-4.19171300
C	3.28473700	-3.19790400	-2.28487500
C	4.48907000	-3.09274600	-3.00936700
H	4.45820400	-2.85424800	-4.07207700
C	5.71578200	-3.28591700	-2.38070900
C	7.08831600	-3.14236100	-3.03522900
H	7.03020400	-2.86217500	-4.09269500
C	7.84206300	-2.11360200	-2.18227400
C	7.92026800	-2.47061100	-0.82237700
C	8.58003400	-1.64811900	0.08558000
H	8.63599200	-1.92645400	1.13707100
C	9.18335500	-0.46442400	-0.37526900
H	9.73180500	0.17225700	0.31562800
C	9.08799300	-0.10175000	-1.71921400
H	9.54799400	0.81842900	-2.06393100
C	7.22455300	-3.80358400	-0.51435700
C	7.88980200	-4.83057500	-1.44476700
C	8.55454000	-5.99574300	-1.07154600
C	9.08430100	-6.83831700	-2.06594500
C	8.93135200	-6.51790800	-3.41766800
C	8.27844800	-5.33133600	-3.79155500
H	8.14239900	-5.06646100	-4.83791900
H	9.32428900	-7.18282700	-4.18296900
H	9.61138900	-7.74693600	-1.78252800
H	8.66773100	-6.24508100	-0.01943200
H	7.28174100	-4.08236500	0.54254100

C	8.40548300	-0.92395400	-2.62939600
H	8.30610500	-0.63023400	-3.67097400
C	7.78707200	-4.48464500	-2.80495300
C	5.77976100	-3.63323700	-1.01867300
C	4.59848700	-3.75043300	-0.28272500
H	4.63851500	-4.00309600	0.77798600
C	3.34107400	-3.52622500	-0.89419000
B	2.02090000	-3.60423500	-0.07286100
C	2.03625500	-3.94050800	1.46391300
C	1.91908100	-2.90549000	2.42307100
C	1.73637800	-1.45714500	1.98882200
H	2.34729900	-1.21388300	1.10950800
H	2.00746100	-0.76444700	2.79564700
H	0.68951000	-1.25745000	1.71632500
C	1.94963000	-3.20824700	3.79145600
H	1.90855400	-2.38976200	4.50590700
C	2.05344700	-4.52424900	4.25588500
C	2.05983900	-4.83735900	5.74264400
H	2.47575800	-4.00323700	6.32181100
H	2.65746700	-5.73269700	5.95719500
H	1.04111600	-5.02231800	6.11519000
C	2.13906100	-5.54659500	3.30197900
H	2.21231500	-6.57873600	3.64047700
C	2.14940600	-5.27622200	1.92629000
C	2.27841100	-6.42276400	0.93247200
H	3.15907500	-6.29012400	0.28833600
H	1.40130300	-6.48239700	0.27169900
H	2.37858100	-7.38517500	1.44751900
C	0.66242800	-3.32941900	-0.79134200
C	-0.54227600	-3.43674400	-0.06770700
C	-1.76879700	-3.24444500	-0.69705200
C	-1.83253600	-2.89623200	-2.05886300
C	-0.65098800	-2.77717200	-2.79398800
H	-0.69064700	-2.52396600	-3.85458600
C	-3.27725300	-2.72667300	-2.56394300
H	-3.33392400	-2.44733700	-3.62075400
C	-3.94337900	-1.70045200	-1.63328800
C	-3.84042700	-2.04686300	-0.27326500
C	-4.33061700	-1.19998700	0.71371300
H	-4.19301900	-1.46486000	1.75984700
C	-4.98356900	-0.01324500	0.34047800
H	-5.37571300	0.65158200	1.10622300
C	-5.13737500	0.30735800	-1.01112500
H	-5.66428900	1.21618200	-1.29426600
C	-4.60806900	-0.53504000	-2.00593700
H	-4.72122500	-0.28513600	-3.05794100
C	-3.97215600	-4.06034300	-2.25698000
C	-4.63058400	-4.88321300	-3.16565300
H	-4.68643400	-4.60454000	-4.21707300
C	-5.23273900	-6.06785000	-2.70565200
H	-5.78036900	-6.70469700	-3.39705200
C	-5.13739800	-6.43121800	-1.36185900
H	-5.59627900	-7.35225300	-1.01777300

C	-4.45613500	-5.60866400	-0.45104800
H	-4.35630800	-5.90299000	0.59030300
C	-3.89416700	-4.41796800	-0.89722400
C	-3.14155900	-3.38915300	-0.04335100
H	-3.08389800	-3.66979900	1.01401400
H	-0.51182600	-3.67614700	0.99483500
C	0.60629500	-3.00018800	-2.18185800

Table S12. Cartesian coordinates of optimized S_1 state of MesDBPI as the QM layer of ONIOM model at BMK/6-31G(d) in the solid phase

Elements	X	Y	Z
B	1.92290000	-2.91046000	-3.03036000
C	1.90274000	-2.57644000	-4.57648000
C	1.78761000	-1.24102000	-5.04580000
C	1.65093000	-0.09092000	-4.05711000
H	1.56475000	0.87062000	-4.57666000
H	2.51495000	-0.03868000	-3.37965000
H	0.76007000	-0.21554000	-3.42537000
C	1.79891000	-0.96645000	-6.42112000
H	1.72569000	0.06601000	-6.75847000
C	1.88693000	-1.98595000	-7.37730000
C	1.88187000	-1.67136000	-8.86388000
H	2.89967000	-1.47878000	-9.23557000
H	1.27837000	-0.78006000	-9.07950000
H	1.47287000	-2.50809000	-9.44455000
C	1.99145000	-3.30137000	-6.91350000
H	2.03164000	-4.11878000	-7.62915000
C	2.02056000	-3.60604000	-5.54472000
C	2.20253000	-5.05880000	-5.12552000
H	3.24918000	-5.26152000	-4.85251000
H	1.93489000	-5.74310000	-5.94104000
H	1.59435000	-5.30543000	-4.24683000
C	3.24956000	-3.20053000	-2.31468000
C	4.47332000	-3.11819000	-3.03848000
H	4.44001000	-2.88113000	-4.10119000
C	5.68525000	-3.30139000	-2.41359000
C	7.05776000	-3.16106000	-3.05778000
H	7.00317000	-2.88357000	-4.11581000
C	7.80263000	-2.12968000	-2.20357000
C	7.88412000	-2.48274000	-0.84096000
C	8.54591000	-1.65503000	0.06401000
H	8.60532000	-1.93043000	1.11600000
C	9.15000000	-0.47657000	-0.40288000
H	9.70407000	0.16057000	0.28306000
C	9.05139000	-0.11752000	-1.75021000
H	9.51474000	0.79958000	-2.09916000
C	7.20077000	-3.81747000	-0.52547000
C	7.85375000	-4.84670000	-1.45835000
C	8.52383000	-6.01006000	-1.08349000

C	9.05498000	-6.85093000	-2.07589000
C	8.89362000	-6.53697000	-3.43047000
C	8.23255000	-5.35849000	-3.80748000
H	8.09307000	-5.09961000	-4.85492000
H	9.29004000	-7.20169000	-4.19395000
H	9.59104000	-7.75402000	-1.79195000
H	8.64171000	-6.25469000	-0.03096000
H	7.26524000	-4.09352000	0.53152000
C	8.36509000	-0.93934000	-2.65504000
H	8.26542000	-0.65095000	-3.69794000
C	7.74519000	-4.50569000	-2.82118000
C	5.75485000	-3.64722000	-1.01283000
C	4.59268000	-3.75325000	-0.27669000
H	4.63448000	-4.00051000	0.78484000
C	3.31110000	-3.53560000	-0.87796000
B	2.02452000	-3.62087000	-0.04875000
C	2.04466000	-3.95580000	1.49716000
C	1.92714000	-2.92682000	2.46604000
C	1.74471000	-1.47377000	2.04800000
H	2.35153000	-1.22663000	1.16853000
H	2.01359000	-0.78997000	2.86354000
H	0.69765000	-1.27082000	1.77672000
C	1.95685000	-3.23245000	3.83456000
H	1.91764000	-2.41544000	4.55056000
C	2.06115000	-4.54811000	4.29765000
C	2.06643000	-4.86334000	5.78410000
H	2.47534000	-4.02677000	6.36508000
H	2.67010000	-5.75463000	5.99933000
H	1.04870000	-5.05622000	6.15583000
C	2.14867000	-5.56708000	3.34081000
H	2.22173000	-6.59982000	3.67740000
C	2.15976000	-5.29154000	1.96567000
C	2.29631000	-6.44099000	0.97621000
H	3.18834000	-6.31710000	0.34594000
H	1.43328000	-6.49142000	0.29736000
H	2.38036000	-7.40311000	1.49504000
C	0.69786000	-3.33068000	-0.76439000
C	-0.52591000	-3.41327000	-0.04067000
C	-1.73776000	-3.22962000	-0.66556000
C	-1.80741000	-2.88335000	-2.06624000
C	-0.64523000	-2.77740000	-2.80233000
H	-0.68694000	-2.52998000	-3.86383000
C	-3.25340000	-2.71310000	-2.55366000
H	-3.31775000	-2.43672000	-3.61059000
C	-3.90670000	-1.68431000	-1.62049000
C	-3.79766000	-2.02555000	-0.25778000
C	-4.28409000	-1.17263000	0.72887000
H	-4.14328000	-1.43142000	1.77613000
C	-4.94550000	0.00582000	0.35241000
H	-5.34137000	0.67035000	1.11629000

C	-5.10779000	0.31982000	-1.00207000
H	-5.64394000	1.22295000	-1.28579000
C	-4.57702000	-0.52089000	-1.99484000
H	-4.69515000	-0.27591000	-3.04727000
C	-3.93610000	-4.04833000	-2.23868000
C	-4.59665000	-4.87671000	-3.14403000
H	-4.65613000	-4.60126000	-4.19600000
C	-5.19936000	-6.05606000	-2.67757000
H	-5.75256000	-6.69365000	-3.36379000
C	-5.10050000	-6.41543000	-1.33030000
H	-5.56258000	-7.33334000	-0.98165000
C	-4.41533000	-5.59298000	-0.42518000
H	-4.31497000	-5.88168000	0.61756000
C	-3.85448000	-4.40169000	-0.87615000
C	-3.11024000	-3.37018000	-0.02151000
H	-3.05573000	-3.64795000	1.03645000
H	-0.49273000	-3.65106000	1.02191000
C	0.63633000	-2.99532000	-2.20106000

Table S13. Cartesian coordinates of optimized T_1 state of MesDBPI as the QM layer of ONIOM model at BMK/6-31G(d) in the solid phase

Elements	X	Y	Z
B	1.92179000	-2.92105000	-3.03103000
C	1.90226000	-2.58421000	-4.57561000
C	1.78938000	-1.24796000	-5.04073000
C	1.65466000	-0.09923000	-4.04994000
H	1.57711000	0.86373000	-4.56804000
H	2.51608000	-0.05250000	-3.36867000
H	0.75928000	-0.21887000	-3.42363000
C	1.80049000	-0.97216000	-6.41582000
H	1.72834000	0.06084000	-6.75173000
C	1.88644000	-1.99079000	-7.37315000
C	1.88107000	-1.67421000	-8.85929000
H	2.89899000	-1.48210000	-9.23075000
H	1.27832000	-0.78208000	-9.07338000
H	1.47110000	-2.50975000	-9.44092000
C	1.98974000	-3.30709000	-6.91134000
H	2.02956000	-4.12361000	-7.62790000
C	2.01904000	-3.61335000	-5.54311000
C	2.20261000	-5.06532000	-5.12207000
H	3.24838000	-5.26437000	-4.84319000
H	1.94108000	-5.75111000	-5.93817000
H	1.58967000	-5.31480000	-4.24723000
C	3.25119000	-3.19786000	-2.31546000
C	4.47584000	-3.10691000	-3.03817000
H	4.44202000	-2.87040000	-4.10103000

C	5.68629000	-3.29354000	-2.41513000
C	7.06093000	-3.15625000	-3.05623000
H	7.00875000	-2.87766000	-4.11421000
C	7.80620000	-2.12599000	-2.20037000
C	7.88704000	-2.48032000	-0.83874000
C	8.54902000	-1.65410000	0.06684000
H	8.60809000	-1.93060000	1.11861000
C	9.15400000	-0.47491000	-0.39844000
H	9.70809000	0.16105000	0.28861000
C	9.05602000	-0.11459000	-1.74469000
H	9.51991000	0.80251000	-2.09291000
C	7.20291000	-3.81550000	-0.52409000
C	7.85494000	-4.84464000	-1.45837000
C	8.52316000	-6.00913000	-1.08441000
C	9.05457000	-6.84987000	-2.07726000
C	8.89510000	-6.53401000	-3.43089000
C	8.23557000	-5.35367000	-3.80675000
H	8.09752000	-5.09317000	-4.85402000
H	9.29130000	-7.19811000	-4.19508000
H	9.58920000	-7.75390000	-1.79352000
H	8.63978000	-6.25513000	-0.03200000
H	7.26900000	-4.09263000	0.53261000
C	8.36933000	-0.93565000	-2.65044000
H	8.27027000	-0.64619000	-3.69317000
C	7.74766000	-4.50203000	-2.82027000
C	5.75559000	-3.64649000	-1.00855000
C	4.59498000	-3.75696000	-0.27490000
H	4.63612000	-4.00720000	0.78601000
C	3.31284000	-3.53082000	-0.87561000
B	2.02476000	-3.61012000	-0.04861000
C	2.04439000	-3.94791000	1.49574000
C	1.92802000	-2.91934000	2.46385000
C	1.74396000	-1.46709000	2.04393000
H	2.35576000	-1.21704000	1.16847000
H	2.00643000	-0.78176000	2.86012000
H	0.69782000	-1.26793000	1.76647000
C	1.95809000	-3.22653000	3.83182000
H	1.91931000	-2.41040000	4.54873000
C	2.06128000	-4.54307000	4.29293000
C	2.06696000	-4.86024000	5.77896000
H	2.47700000	-4.02491000	6.36083000
H	2.66973000	-5.75246000	5.99263000
H	1.04910000	-5.05246000	6.15054000
C	2.14675000	-5.56118000	3.33498000
H	2.21893000	-6.59446000	3.67012000
C	2.15743000	-5.28445000	1.96005000
C	2.29196000	-6.43252000	0.96849000
H	3.18804000	-6.31321000	0.34310000
H	1.43115000	-6.47788000	0.28635000
H	2.36810000	-7.39599000	1.48588000

C	0.69536000	-3.33326000	-0.76412000
C	-0.52928000	-3.42467000	-0.04151000
C	-1.73967000	-3.23766000	-0.66454000
C	-1.80904000	-2.88413000	-2.07104000
C	-0.64844000	-2.77355000	-2.80464000
H	-0.68953000	-2.52307000	-3.86549000
C	-3.25646000	-2.71520000	-2.55550000
H	-3.32250000	-2.43769000	-3.61213000
C	-3.90877000	-1.68657000	-1.62084000
C	-3.80094000	-2.02949000	-0.25908000
C	-4.28765000	-1.17769000	0.72783000
H	-4.14811000	-1.43817000	1.77489000
C	-4.94736000	0.00274000	0.35267000
H	-5.34280000	0.66669000	1.11735000
C	-5.10787000	0.31873000	-1.00083000
H	-5.64248000	1.22290000	-1.28422000
C	-4.57709000	-0.52188000	-1.99414000
H	-4.69404000	-0.27548000	-3.04643000
C	-3.93982000	-4.05097000	-2.24143000
C	-4.60037000	-4.87790000	-3.14747000
H	-4.65944000	-4.60131000	-4.19923000
C	-5.20386000	-6.05808000	-2.68271000
H	-5.75698000	-6.69452000	-3.37010000
C	-5.10567000	-6.41878000	-1.33654000
H	-5.56820000	-7.33676000	-0.98872000
C	-4.42023000	-5.59707000	-0.43043000
H	-4.32047000	-5.88694000	0.61211000
C	-3.85884000	-4.40568000	-0.87990000
C	-3.11426000	-3.37531000	-0.02357000
H	-3.06211000	-3.65423000	1.03432000
H	-0.49558000	-3.66206000	1.02118000
C	0.63372000	-2.99991000	-2.20396000

Table S14. Cartesian coordinates of optimized S_0 state of MesDBA as the QM layer of ONIOM model at BMK/6-31G(d) in the solid phase

Elements	X	Y	Z
C	-0.23731913	-3.07499364	-0.02811658
B	-0.05619633	-1.50961317	-0.00118955
C	-0.02204622	-0.71545541	1.34276403
C	-0.05282024	-1.38422026	2.58041453
C	0.03553678	-0.68563783	3.79381402
H	0.00033933	-1.22430404	4.73821428
C	0.15905599	0.70559881	3.78178941
H	0.25676056	1.26247869	4.70847265
C	0.14571297	1.39693539	2.56338281
H	0.20878453	2.48464848	2.56378610
H	-0.15649127	-2.46670783	2.59007464

C	0.04937208	0.71491945	1.33825026
B	-0.05619633	1.50291350	-0.00118955
C	-0.33696142	3.04697706	0.02059445
C	-1.67894162	3.50195636	0.06661174
C	-1.94701243	4.87122498	0.15852671
H	-2.98357244	5.20288318	0.20298375
C	-0.91814452	5.82176671	0.21316296
C	-1.25076803	7.29112626	0.37834720
H	-1.45460249	7.52937060	1.42990819
H	-0.42838301	7.93507044	0.04398558
H	-2.15280534	7.54675480	-0.19049922
C	0.40421454	5.37073106	0.13991756
H	1.21795573	6.09505678	0.16955721
C	-2.83340135	2.51168011	0.01099416
H	-3.79425998	3.01784955	0.16465991
H	-2.73414138	1.73475663	0.78282138
H	-2.87080759	2.00108142	-0.96252480
C	0.70421205	4.00293048	0.03725939
C	2.14960164	3.54560050	-0.08810196
H	2.32984477	2.63766907	0.50137855
H	2.85217303	4.31698281	0.24876984
H	2.39552981	3.30098105	-1.13160451
C	0.03090569	0.71909812	-1.33869987
C	0.06998778	1.40779314	-2.56362980
H	0.03024843	2.49693108	-2.56071715
C	0.14853977	0.71943654	-3.78180847
C	0.17654448	-0.67732053	-3.78608559
C	0.12548073	-1.38090727	-2.57391532
H	0.13827614	-2.46825296	-2.58167761
H	0.22184025	-1.22353964	-4.72443136
H	0.17050948	1.27705147	-4.71425746
C	0.05416110	-0.71186249	-1.33993532
C	-1.54324189	-3.62327796	-0.10553611
C	-2.75890826	-2.70870961	-0.14653926
H	-2.63343695	-1.84299456	0.51817311
H	-3.66254790	-3.24281704	0.17008266
H	-2.93385540	-2.32186344	-1.16162576
C	-1.72553832	-5.01176950	-0.15955932
H	-2.73941853	-5.41291622	-0.20488942
C	-0.63731718	-5.89536931	-0.14479228
C	0.65048152	-5.35190947	-0.06593763
H	1.50956437	-6.02214175	-0.03522502
C	-0.85623389	-7.39583930	-0.21606152

H	-1.59921419	-7.72605132	0.52292822
H	0.07540606	-7.94453701	-0.03015409
H	-1.22762570	-7.68864938	-1.20771923
C	0.86333989	-3.96601460	-0.00084096
C	2.28061756	-3.42922674	0.13159515
H	2.42069941	-2.90821547	1.09022971
H	3.02296522	-4.23438347	0.07416702
H	2.50174479	-2.70758817	-0.66447054

Table S15. Cartesian coordinates of optimized S_1 state of MesDBA as the QM layer of ONIOM model at BMK/6-31G(d) in the solid phase

Elements	X	Y	Z
C	-3.36973800	-0.47588500	1.11358600
B	-2.85469400	1.02090600	1.01848600
C	-2.35751200	1.79135800	2.25816800
C	-2.23635300	1.15440700	3.52145800
C	-1.75523400	1.81230500	4.64938100
H	-1.68743700	1.29430300	5.60351000
C	-1.36133600	3.16371800	4.54607600
H	-0.94854900	3.68786800	5.40310900
C	-1.49815000	3.83252700	3.33944300
H	-1.18905400	4.87796800	3.28467200
H	-2.54322900	0.11333600	3.59898000
C	-2.00449100	3.18914100	2.17239500
B	-2.20263000	3.93767700	0.85873400
C	-2.12829100	5.50190400	0.83808500
C	-3.32095200	6.28325200	1.15509700
C	-3.22864200	7.65477500	1.26472300
H	-4.10969300	8.23766500	1.52274400
C	-1.99834900	8.33451000	1.05365000
C	-1.94414700	9.82507600	1.22451700
H	-1.90085300	10.06506100	2.29689500
H	-1.06714500	10.26886800	0.74187400
H	-2.85845300	10.28919300	0.83634100
C	-0.84072100	7.59527200	0.69735800
H	0.08996600	8.13146700	0.52805900
C	-4.63670200	5.57924900	1.37645900
H	-5.42759200	6.29463500	1.62786800
H	-4.54155800	4.84237600	2.18438900
H	-4.92391800	5.02103300	0.47543100
C	-0.89004500	6.22084800	0.55936800
C	0.31514800	5.42276200	0.13681400
H	0.44900300	4.56754900	0.80687200

H	1.22567300	6.02981000	0.12410000
H	0.14808500	5.00178900	-0.86510600
C	-2.61645200	3.18979800	-0.40488300
C	-2.73860600	3.84218500	-1.66706500
H	-2.53707500	4.91261100	-1.73893700
C	-3.10180800	3.15438100	-2.81516400
C	-3.36962700	1.76977700	-2.74531200
C	-3.27090800	1.11250000	-1.52359200
H	-3.48176500	0.04618400	-1.46911200
H	-3.66429500	1.22133900	-3.63605500
H	-3.18949700	3.68850600	-3.75814100
C	-2.90430200	1.77779000	-0.32479000
C	-4.74951200	-0.72945700	1.34137400
C	-5.72135700	0.42716300	1.52596900
H	-5.27966900	1.21912100	2.14440300
H	-6.64471000	0.08954200	2.01196900
H	-5.98909200	0.87992000	0.55950900
C	-5.23776200	-2.04232800	1.39424600
H	-6.30007800	-2.20848000	1.58296000
C	-4.39080700	-3.14617300	1.22239000
C	-3.03014600	-2.90080500	1.00532400
H	-2.35081400	-3.74564800	0.89073900
C	-4.93881900	-4.56136100	1.27228800
H	-5.54816400	-4.72265700	2.17263300
H	-4.12948900	-5.30246500	1.27321800
H	-5.57995400	-4.76466200	0.40322800
C	-2.51371800	-1.59557900	0.95799100
C	-1.01738000	-1.39657600	0.76840800
H	-0.58147900	-0.85363200	1.61933100
H	-0.49485500	-2.35638200	0.67044800
H	-0.81150600	-0.80112400	-0.12909200

Table S16. Cartesian coordinates of optimized T_1 state of MesDBA as the QM layer of ONIOM model at BMK/6-31G(d) in the solid phase

Elements	X	Y	Z
C	-3.36741500	-0.52173800	1.10912100
B	-2.85170700	0.97122600	1.01785800
C	-2.37482000	1.74650600	2.25343300
C	-2.29396400	1.12128800	3.53458200
C	-1.79029400	1.78083500	4.63889200
H	-1.73401000	1.28131300	5.60364700
C	-1.33350600	3.14496400	4.52000600
H	-0.91141500	3.65102800	5.38279900

C	-1.44603800	3.81135200	3.31827300
H	-1.13144300	4.85128000	3.24273100
H	-2.63520000	0.09327000	3.62440600
C	-1.99650700	3.18094200	2.16073100
B	-2.23148200	3.97884500	0.87560600
C	-2.13665500	5.55087200	0.87769400
C	-3.29645400	6.30571900	1.19166400
C	-3.22497600	7.69983100	1.26966800
H	-4.12397500	8.26093000	1.52040200
C	-2.02462700	8.38895900	1.05127300
C	-1.97927700	9.89498700	1.20787600
H	-1.88831100	10.17557500	2.26450700
H	-1.13121500	10.33686200	0.67096700
H	-2.90769500	10.34341200	0.83489700
C	-0.88757700	7.64724000	0.71545000
H	0.05221400	8.16671200	0.53027400
C	-4.62997000	5.61492800	1.44159000
H	-5.39598400	6.33850200	1.74677500
H	-4.54829000	4.85345400	2.23003200
H	-4.98798400	5.10274800	0.53651900
C	-0.93240900	6.24675700	0.61894300
C	0.31330800	5.47765000	0.20566900
H	0.42373300	4.55850100	0.79323300
H	1.22204800	6.07864100	0.32835400
H	0.25457000	5.17145500	-0.84914400
C	-2.60454900	3.18966700	-0.37749100
C	-2.69024600	3.84537600	-1.64301000
H	-2.48460000	4.91430800	-1.68476800
C	-3.05050200	3.16508300	-2.78801500
C	-3.34222300	1.75450700	-2.72630100
C	-3.26534700	1.08003800	-1.52338500
H	-3.48196700	0.01589200	-1.48126900
H	-3.62980700	1.22650000	-3.63155900
H	-3.13224700	3.69262300	-3.73467400
C	-2.89441600	1.73615100	-0.31265500
C	-4.74706300	-0.77426600	1.33465800
C	-5.72706300	0.37734400	1.51461200
H	-5.28828800	1.19154100	2.10539800
H	-6.63493000	0.04104900	2.02913900
H	-6.02709700	0.80350400	0.54536600
C	-5.23040500	-2.08939300	1.38868200
H	-6.29222100	-2.25849600	1.57626200
C	-4.37982300	-3.19054500	1.22016500

C	-3.01940100	-2.94252900	1.00399800
H	-2.33846600	-3.78594100	0.89343700
C	-4.92256700	-4.60733700	1.27039700
H	-5.56697700	-4.75700400	2.14754300
H	-4.11078900	-5.34413300	1.31453600
H	-5.52630500	-4.82600800	0.37887700
C	-2.50603500	-1.63681700	0.95430500
C	-1.00961800	-1.43304200	0.76729700
H	-0.57103500	-0.90209300	1.62484500
H	-0.48579500	-2.39048000	0.65902500
H	-0.79963700	-0.82866100	-0.12366800

Table S17. Cartesian coordinates of optimized S_0 state of MesDBP as the QM layer of ONIOM model at BMK/6-31G(d) in the solid phase

Elements	X	Y	Z
B	3.04213500	4.83423700	5.53184100
C	3.26603600	3.96619800	6.83053000
C	3.84461700	2.67216100	6.73940600
C	4.04157700	1.90103300	7.89259400
H	4.47804500	0.90514700	7.79456500
C	3.66973900	2.36654600	9.16207100
C	3.86776900	1.49903200	10.39206900
H	3.40093700	0.51301100	10.25901300
H	4.93570500	1.33064200	10.59151000
H	3.42686800	1.96591000	11.28140000
C	3.11220200	3.64671900	9.25272800
H	2.82134300	4.03411600	10.22957700
C	4.28493400	2.10655100	5.39871800
H	4.35790800	1.01421200	5.44772200
H	3.57766300	2.36404900	4.60128200
H	5.27061400	2.49834100	5.10744700
C	2.91597100	4.44813300	8.11629000
C	2.29393800	5.82600400	8.28553400
H	1.21807900	5.80898800	8.05704900
H	2.41325100	6.18783700	9.31345400
H	2.76376900	6.55786300	7.61709100
C	1.83138800	4.58103800	4.58743400
C	0.86524300	3.64881400	4.93204800
H	0.95110200	3.12453100	5.88107900
C	-0.23831200	3.34456900	4.08237100
C	-1.22371300	2.38139900	4.42367400

H	-1.13881300	1.91141700	5.38804400
C	-2.28623100	2.10131200	3.58978100
H	-3.03184800	1.36398500	3.88087600
C	-2.41201700	2.79654600	2.35808900
H	-3.25558200	2.59331800	1.70085600
C	-1.46020700	3.72819600	1.99958600
H	-1.52779900	4.26099900	1.06556800
C	-0.36036700	4.03432300	2.84236400
C	0.61196600	5.02181300	2.50592800
H	0.49469000	5.56268300	1.56590700
C	1.68955200	5.30931000	3.33185400
B	2.71047000	6.41283100	2.93353300
C	3.91703600	6.67256600	3.88467500
C	4.88186900	7.61016800	3.54989900
C	5.98046000	7.91502000	4.40722800
C	6.10262900	7.21775800	5.64323700
C	5.13310300	6.22496000	5.96904000
H	5.24909100	5.67731100	6.90525700
C	7.19897100	7.52047400	6.49233900
H	7.26916100	6.97702000	7.42101700
C	8.14656000	8.46045200	6.14435100
H	8.98797000	8.66210600	6.80481900
C	8.02090800	9.16435600	4.91750500
H	8.76417200	9.90720300	4.63435600
C	6.96320800	8.88480800	4.07714600
H	6.88072900	9.36179600	3.11632200
H	4.79971600	8.13788400	2.60237900
C	2.49487600	7.26746700	1.62310000
C	2.83326000	6.76066300	0.34263700
C	3.43776100	5.37328400	0.18864900
H	2.96529400	4.65580200	0.87041900
H	3.30649000	4.99878600	-0.83341100
H	4.51564900	5.38217300	0.40759200
C	2.63459400	7.54233100	-0.80642800
H	2.90564700	7.13089700	-1.77876500
C	2.09141400	8.82995400	-0.73696200
C	1.73176400	9.32084300	0.52626300
H	1.29777600	10.31910800	0.60834600
C	1.90973800	9.67512800	-1.98554900
H	1.97822800	9.06050800	-2.89174700
H	0.93464700	10.18016100	-1.98706100
H	2.68344600	10.45433300	-2.05148900
C	1.92640400	8.56771900	1.69322500

C	1.48333400	9.15450200	3.02518500
H	0.51681100	8.73425300	3.34064800
H	2.20880100	8.94370200	3.82056800
H	1.36761700	10.24196400	2.95004000
C	4.05903900	5.94035400	5.13801000

Table S18. Cartesian coordinates of optimized S_1 state of MesDBP as the QM layer of ONIOM model at BMK/6-31G(d) in the solid phase

Elements	X	Y	Z
B	3.04162100	4.87277600	5.52680200
C	3.27857500	4.02210100	6.86601500
C	3.94232800	2.73167500	6.81598000
C	4.03429200	1.96111400	7.95399800
H	4.47956700	0.97064200	7.90335400
C	3.53468200	2.41731100	9.20597600
C	3.58367600	1.52325700	10.40177100
H	2.80087300	0.75392100	10.31568300
H	4.53820700	0.98844600	10.46313800
H	3.41203400	2.06881300	11.33291500
C	2.97143000	3.71218100	9.28559000
H	2.63610200	4.07453200	10.25152500
C	4.48602500	2.22472600	5.51274300
H	4.91465400	1.22885900	5.63787600
H	3.70054800	2.18662200	4.75253100
H	5.25962000	2.90073900	5.13408500
C	2.84987500	4.51199500	8.16831200
C	2.27311700	5.89163700	8.27658800
H	1.24227300	5.89674900	7.90098500
H	2.27102200	6.24214000	9.31054500
H	2.83978000	6.59589600	7.66383200
C	1.83303300	4.60823300	4.63096500
C	0.84898300	3.65581800	4.94654700
H	0.90934700	3.11582700	5.88757800
C	-0.23062600	3.35560100	4.10048000
C	-1.22060600	2.38107100	4.40986800
H	-1.14652100	1.89390400	5.36544600
C	-2.27070800	2.11149700	3.56846000
H	-3.01342900	1.36643600	3.84259600
C	-2.39648400	2.82573500	2.34660700
H	-3.23437500	2.62863100	1.68315000
C	-1.45095500	3.76629400	2.01582400

H	-1.51915200	4.31976500	1.09531800
C	-0.35596100	4.06793800	2.86340500
C	0.60325000	5.05477900	2.55305400
H	0.48660000	5.60538100	1.62131600
C	1.68857700	5.34900500	3.37605600
B	2.71646800	6.43008500	2.95793600
C	3.93173900	6.66563100	3.89643100
C	4.90031800	7.61119500	3.57282100
C	5.99634700	7.91865500	4.40785600
C	6.13692600	7.21013800	5.64480100
C	5.18910600	6.22635200	5.96949400
H	5.33315100	5.69631900	6.91091700
C	7.24525000	7.53038900	6.47694300
H	7.33687400	6.98827400	7.40295800
C	8.17406600	8.47667800	6.12326100
H	9.01787600	8.68616800	6.77627700
C	8.03145200	9.18385300	4.89966800
H	8.76406200	9.93255200	4.61104700
C	6.96925900	8.89448600	4.07677400
H	6.87010200	9.37135000	3.11962100
H	4.80344000	8.14627900	2.63321900
C	2.50200100	7.28607300	1.62562300
C	2.85889500	6.78261600	0.35160500
C	3.47275300	5.40641400	0.20193900
H	3.00713600	4.68594800	0.88042100
H	3.35073300	5.02878300	-0.81853200
H	4.54817800	5.41750700	0.42560000
C	2.66029900	7.55317300	-0.80117200
H	2.94259700	7.13867400	-1.76720000
C	2.10518800	8.83226000	-0.74818100
C	1.73036400	9.32164900	0.50667600
H	1.28748000	10.31495100	0.57981700
C	1.93062600	9.66413400	-1.99855300
H	1.98640700	9.04411700	-2.90005100
H	0.96523600	10.18434800	-2.00669100
H	2.71063100	10.43382800	-2.08305700
C	1.91716500	8.57718000	1.67705200
C	1.45541100	9.17257400	2.99104300
H	0.50140000	8.73550700	3.31612200
H	2.17778200	8.99524800	3.79369300
H	1.31008900	10.25362200	2.89783000
C	4.08756200	5.92229300	5.14825300

Table S19. Cartesian coordinates of optimized T_1 state of MesDBP as the QM layer of ONIOM model at BMK/6-31G(d) in the solid phase

Elements	X	Y	Z
B	3.05496300	4.81852700	5.51278300
C	3.25966700	3.94979400	6.81382600
C	3.84083600	2.65688200	6.72444100
C	4.04264600	1.88800300	7.87833000
H	4.48319200	0.89382400	7.78117800
C	3.67159000	2.35474700	9.14744700
C	3.87975700	1.49259000	10.37948600
H	3.44870500	0.49158800	10.23871800
H	4.94981800	1.36032600	10.59451900
H	3.41082200	1.94348200	11.26269800
C	3.10736900	3.63240800	9.23639100
H	2.81575500	4.01997400	10.21300000
C	4.28321700	2.09153600	5.38458500
H	4.34101200	0.99815100	5.42929000
H	3.58667200	2.36156400	4.58174500
H	5.27561200	2.47222100	5.10207000
C	2.90783300	4.43226900	8.09946500
C	2.28347900	5.80911200	8.26855900
H	1.20772200	5.79166500	8.03884600
H	2.40000000	6.16979600	9.29715100
H	2.75361200	6.54172900	7.60150100
C	1.85325100	4.55455200	4.54103500
C	0.85991800	3.64648100	4.91098500
H	0.93080400	3.16316600	5.88375800
C	-0.26397400	3.30962400	4.06873100
C	-1.23991700	2.40540800	4.46612300
H	-1.17293200	2.00209100	5.46267400
C	-2.35516200	2.07372400	3.63712600
H	-3.10413800	1.37021900	3.99177600
C	-2.46401800	2.68225500	2.39463100
H	-3.30964100	2.46372900	1.74489500
C	-1.46660600	3.57599900	1.95634500
H	-1.52044700	4.00885300	0.96980100
C	-0.35898500	3.93365400	2.77807600
C	0.61638000	4.86632800	2.40007300
H	0.53633800	5.36091900	1.43256400
C	1.74838100	5.24124300	3.28130900
B	2.72399400	6.36030700	2.90703500
C	3.91200500	6.64786200	3.86758600

C	4.87759900	7.59626300	3.54198400
C	5.96965400	7.90741100	4.40345000
C	6.09710100	7.21133600	5.64112300
C	5.13022600	6.21420100	5.96274900
H	5.24430700	5.66774600	6.90022000
C	7.19048300	7.51526200	6.49059900
H	7.26192400	6.96990600	7.41844000
C	8.13800500	8.45878400	6.14511300
H	8.97904800	8.66132100	6.80562300
C	8.00860100	9.16199700	4.91934100
H	8.74957600	9.90724600	4.63577900
C	6.95120500	8.88076100	4.07743200
H	6.86944100	9.35954400	3.11729400
H	4.80016000	8.12137600	2.59238700
C	2.48422600	7.22120500	1.59985300
C	2.77940100	6.70122700	0.31121200
C	3.34420000	5.29777400	0.15185800
H	2.83240900	4.58004700	0.80748300
H	3.23534700	4.94482400	-0.88071700
H	4.41346700	5.27015200	0.40712200
C	2.57883600	7.47955400	-0.83913400
H	2.81992200	7.05662700	-1.81460000
C	2.07066100	8.78179700	-0.76632700
C	1.74460900	9.28577100	0.50032900
H	1.33281700	10.29333400	0.58355300
C	1.89136900	9.62435100	-2.01703700
H	1.81263300	8.99298200	-2.91117900
H	0.98739600	10.24418900	-1.95445400
H	2.74586900	10.30188200	-2.16362600
C	1.94521900	8.53519800	1.66903600
C	1.53867300	9.14581000	3.00278100
H	0.58449700	8.72632800	3.35481500
H	2.28758300	8.95082200	3.77938500
H	1.41257300	10.23133600	2.91101800
C	4.06120500	5.92333700	5.12607100

Table S20. Cartesian coordinates of optimized S_0 state of MesDBPI-d_{18} as the QM layer of ONIOM model at BMK/6-31G(d) in the solid phase.

Elements	X	Y	Z
C	-3.34010200	3.52718400	0.89434700
C	-3.28341500	3.20002600	2.28522800
C	-4.48775200	3.09381900	3.00943500
H	-4.45684300	2.85705300	4.07243500
C	-5.71443200	3.28417200	2.38007700
C	-7.08681900	3.13721100	3.03372100
H	-7.02884900	2.85536800	4.09078300
C	-7.83891700	2.10900800	2.17872200
C	-8.40082700	0.91785000	2.62343600
H	-8.30089900	0.62183800	3.66420700
C	-9.08278100	0.09707900	1.71169100
H	-9.54216500	-0.82386700	2.05447900
C	-9.17878900	0.46202900	0.36856700
H	-9.72763900	-0.17359400	-0.32292700
C	-8.57613900	1.64678800	-0.09028600
H	-8.63210900	1.92704700	-1.14124200
C	-7.91744200	2.46814400	0.81937500
C	-7.22330300	3.80225800	0.51379700
C	-7.88998100	4.82671200	1.44569200
C	-8.55591800	5.99160200	1.07420100
C	-9.08516400	6.83294700	2.06987000
C	-8.93026400	6.51145300	3.42106300
C	-8.27677000	5.32469300	3.79318900
H	-8.13886400	5.05872900	4.83896200
H	-9.32210400	7.17564900	4.18736900
H	-9.61305000	7.74151500	1.78794100
H	-8.66999600	6.24149500	0.02241700
H	-7.28053500	4.08285900	-0.54260700
C	-7.78657300	4.47906200	2.80530100
C	-5.77862000	3.63256000	1.01832400
B	-1.92493400	2.92474100	3.00317700
C	-1.90821900	2.59293600	4.54065900
C	-1.79063100	1.25922500	5.00725700
C	-1.79788100	0.99379000	6.38388400
H	-1.72175200	-0.03673800	6.72601000
C	-1.88349200	2.01908600	7.33444100
C	-1.87306800	1.71037900	8.82206500
H	-2.89085400	1.52722400	9.19807800
H	-1.27544200	0.81525100	9.03745600

H	-1.45486500	2.54573100	9.39770100
C	-1.99108300	3.33333900	6.86579100
H	-2.03139000	4.15428000	7.57731700
C	-1.66056300	0.10960700	4.01712700
H	-1.55315700	-0.85030500	4.53526500
H	-2.54032000	0.04310000	3.36056000
H	-0.78354200	0.24408300	3.36851400
C	-2.02601700	3.63110800	5.49647800
C	-2.21417600	5.07741100	5.05772100
H	-3.26236600	5.27252200	4.78699800
H	-1.94365700	5.77360200	5.86174500
H	-1.60624500	5.31996700	4.17613500
C	-0.60531200	2.99891800	2.18101200
C	0.65213200	2.77411000	2.79215000
H	0.69250600	2.52183400	3.85289000
C	1.83311400	2.89073100	2.05585700
C	3.27814900	2.72068800	2.55963800
H	3.33587100	2.44023200	3.61607400
C	3.94437900	1.69593800	1.62756400
C	4.61058300	0.53102200	1.99868700
H	4.72552900	0.28128100	3.05039200
C	5.13900900	-0.31057200	1.00277400
H	5.66697000	-1.21912900	1.28460200
C	4.98287200	0.01049500	-0.34842800
H	5.37406500	-0.65376100	-1.11498000
C	4.32902500	1.19712300	-0.72021800
H	4.18971200	1.46279200	-1.76586600
C	3.84004800	2.04315200	0.26793800
C	3.14054600	3.38507900	0.03959800
C	3.89273900	4.41358400	0.89405700
C	4.45294700	5.60531300	0.44883000
C	5.13386400	6.42738300	1.36017500
C	5.23016200	6.06318100	2.70351400
C	4.62909800	4.87780700	3.16290800
H	4.68515900	4.59834800	4.21408000
H	5.77810300	6.70002800	3.39455400
H	5.59195600	7.34882900	1.01701800
H	4.35202200	5.90087600	-0.59195700
H	3.08204200	3.66667500	-1.01749700
C	3.97172500	4.05500700	2.25353400
C	1.76855000	3.23890100	0.69404800
C	0.54185400	3.43094900	0.06535600
H	0.51089500	3.66854100	-0.99747400

C	-0.66216000	3.32599900	0.79013100
C	-4.59744900	3.75067300	0.28262700
H	-4.63764800	4.00293100	-0.77811000
B	-2.02044900	3.60211600	0.07246300
C	-2.03677500	3.93268300	-1.46504600
C	-1.91489400	2.89246300	-2.41827100
C	-1.72019400	1.44906500	-1.97295600
H	-2.35072900	1.19989300	-1.10921000
H	-1.95686200	0.74768900	-2.78302700
H	-0.67823300	1.27094100	-1.66827300
C	-1.95076600	3.18678800	-3.78807400
H	-1.90784500	2.36427600	-4.49738900
C	-2.06178500	4.49985400	-4.25989200
C	-2.14989200	5.52724600	-3.31194700
H	-2.22749500	6.55695800	-3.65646600
C	-2.07177100	4.80483800	-5.74832000
H	-2.49512500	3.97043600	-6.32159200
H	-2.66446100	5.70278400	-5.96547300
H	-1.05325300	4.98104800	-6.12567000
C	-2.15732100	5.26493600	-1.93452300
C	-2.28937600	6.41652100	-0.94695900
H	-3.16552600	6.28156000	-0.29729200
H	-1.40922600	6.48656500	-0.29127800
H	-2.39927500	7.37506100	-1.46712700

Table S21. Cartesian coordinates of optimized S_1 state of MesDBPI-d_{18} as the QM layer of ONIOM model at BMK/6-31G(d) in the solid phase.

Elements	X	Y	Z
C	-3.31003300	3.53168700	0.87533900
C	-3.24788300	3.19890800	2.31240400
C	-4.47120900	3.11746400	3.03675900
H	-4.43759200	2.88219000	4.09981700
C	-5.68325400	3.29859100	2.41169400
C	-7.05536100	3.15575200	3.05576700
H	-7.00044400	2.87695700	4.11342500
C	-7.79924600	2.12479400	2.20023300
C	-8.36037700	0.93335100	2.65013300
H	-8.25983900	0.64318300	3.69237300
C	-9.04656800	0.11264800	1.74438000
H	-9.50965400	-0.80480300	2.09204900
C	-9.14598700	0.47324600	0.39767200

H	-9.70051600	-0.16328800	-0.28839400
C	-8.54244000	1.65237100	-0.06801400
H	-8.60196500	1.92904100	-1.11966100
C	-7.88137700	2.47936200	0.83805400
C	-7.19937700	3.81491000	0.52417800
C	-7.85312200	4.84220700	1.45842300
C	-8.52458200	6.00511500	1.08507700
C	-9.05515700	6.84504400	2.07853100
C	-8.89179900	6.53040100	3.43267200
C	-8.22994200	5.35194300	3.80819700
H	-8.08875700	5.09225700	4.85517100
H	-9.28738300	7.19445700	4.19701500
H	-9.59212500	7.74795600	1.79578900
H	-8.64373100	6.24988200	0.03281400
H	-7.26425100	4.09235700	-0.53237200
C	-7.74358000	4.50002100	2.82079600
C	-5.75340000	3.64468600	1.01106300
B	-1.92120400	2.90863000	3.02759000
C	-1.89927500	2.57826800	4.57418400
C	-1.78000900	1.24426500	5.04636100
C	-1.78784900	0.97303000	6.42230600
H	-1.71168000	-0.05830600	6.76204600
C	-1.87541400	1.99457500	7.37626100
C	-1.86634100	1.68332700	8.86349200
H	-2.88309300	1.49105100	9.23819700
H	-1.26182200	0.79283900	9.07956000
H	-1.45637500	2.52150900	9.44132100
C	-1.98349400	3.30861200	6.90945600
H	-2.02224300	4.12782500	7.62290200
C	-1.64263000	0.09229500	4.05998700
H	-1.55251700	-0.86778900	4.58142400
H	-2.50800900	0.03608100	3.38459100
H	-0.75356700	0.21782900	3.42596400
C	-2.01739900	3.60993100	5.54012000
C	-2.20508100	5.06125300	5.11848100
H	-3.25407700	5.26124000	4.85256200
H	-1.93335200	5.74800900	5.93057500
H	-1.60371300	5.30715000	4.23494000
C	-0.63527200	2.98994900	2.19728200
C	0.64632600	2.77137500	2.79813900
H	0.68849100	2.52453400	3.85974500
C	1.80810300	2.87685200	2.06153900
C	3.25419600	2.70684300	2.54846200

H	3.31903300	2.42907800	3.60495500
C	3.90831900	1.68007100	1.61388700
C	4.58002900	0.51708100	1.98663100
H	4.69957300	0.27199500	3.03878400
C	5.11012600	-0.32265800	0.99274800
H	5.64724300	-1.22559700	1.27516100
C	4.94573700	-0.00793000	-0.36126400
H	5.34070100	-0.67181400	-1.12603900
C	4.28354700	1.17051300	-0.73616100
H	4.14098700	1.43016900	-1.78293900
C	3.79822200	2.02253000	0.25164500
C	3.10993900	3.36678100	0.01706900
C	3.85309600	4.39790600	0.87302700
C	4.41255700	5.59034900	0.42369700
C	5.09747000	6.41172700	1.32981500
C	5.19707400	6.05079100	2.67645500
C	4.59501400	4.87068400	3.14163100
H	4.65456300	4.59389800	4.19325300
H	5.75062800	6.68783100	3.36285200
H	5.55920900	7.33004200	0.98251100
H	4.31139500	5.88088000	-0.61837400
H	3.05503300	3.64586800	-1.04051000
C	3.93535000	4.04299600	2.23511800
C	1.73788400	3.22353600	0.66104100
C	0.52588700	3.40508200	0.03601300
H	0.49235700	3.64130200	-1.02685900
C	-0.69742700	3.32313800	0.76029300
C	-4.59163100	3.74999400	0.27442000
H	-4.63380700	3.99656200	-0.78724900
B	-2.02406200	3.61362000	0.04517700
C	-2.04606500	3.94470200	-1.50124000
C	-1.92825800	2.91344900	-2.46762700
C	-1.74011000	1.46193900	-2.04677600
H	-2.34088400	1.21550900	-1.16297900
H	-2.01210500	0.77542100	-2.85899200
H	-0.69092900	1.26209300	-1.78150800
C	-1.96282700	3.21548800	-3.83674100
H	-1.92507300	2.39655800	-4.55041400
C	-2.07078300	4.52970300	-4.30305800
C	-2.15803400	5.55087200	-3.34864800
H	-2.23409200	6.58246600	-3.68775200
C	-2.07995600	4.84123600	-5.79024300
H	-2.49155400	4.00381800	-6.36803400

H	-2.68301500	5.73278800	-6.00597400
H	-1.06301700	5.03175800	-6.16534100
C	-2.16562200	5.27889400	-1.97281900
C	-2.30330300	6.43034400	-0.98587800
H	-3.19427300	6.30600700	-0.35424300
H	-1.43959200	6.48434200	-0.30819500
H	-2.39030500	7.39101500	-1.50677100

Table S22. Cartesian coordinates of optimized T_1 state of MesDBPI-d_{18} as the QM layer of ONIOM model at BMK/6-31G(d) in the solid phase.

Elements	X	Y	Z
C	-3.31205300	3.52681400	0.87245600
C	-3.24985600	3.19616000	2.31267500
C	-4.47407400	3.10607700	3.03590700
H	-4.43992400	2.87143900	4.09912600
C	-5.68465000	3.29067800	2.41269700
C	-7.05889500	3.15096800	3.05368900
H	-7.00640300	2.87105500	4.11128800
C	-7.80320700	2.12118400	2.19649200
C	-8.36505800	0.92976300	2.64498800
H	-8.26519400	0.63853500	3.68707500
C	-9.05155100	0.10979300	1.73828800
H	-9.51516800	-0.80766800	2.08518000
C	-9.15024700	0.47164800	0.39264600
H	-9.70474300	-0.16374100	-0.29454400
C	-8.54583600	1.65152800	-0.07141000
H	-8.60496200	1.92930700	-1.12284400
C	-7.88463400	2.47703100	0.83530400
C	-7.20182700	3.81303800	0.52228600
C	-7.85454400	4.84025400	1.45798000
C	-8.52410200	6.00435600	1.08558900
C	-9.05490100	6.84412300	2.07949800
C	-8.89347700	6.52749900	3.43269400
C	-8.23323800	5.34715700	3.80701100
H	-8.09352600	5.08577900	4.85381700
H	-9.28885500	7.19090300	4.19777500
H	-9.59041400	7.74800500	1.79701100
H	-8.64199000	6.25048400	0.03344800
H	-7.26832000	4.09158600	-0.53396900
C	-7.74632800	4.49642200	2.81940600
C	-5.75444100	3.64392100	1.00623700

B	-1.92050300	2.91936700	3.02790000
C	-1.89932700	2.58617300	4.57298800
C	-1.78237300	1.25134400	5.04112100
C	-1.78993000	0.97906800	6.41685600
H	-1.71489500	-0.05280300	6.75528800
C	-1.87516100	1.99991100	7.37185500
C	-1.86559200	1.68678300	8.85868400
H	-2.88222500	1.49320200	9.23287900
H	-1.26035000	0.79650500	9.07343100
H	-1.45626300	2.52454100	9.43750000
C	-1.98190800	3.31475500	6.90689800
H	-2.02017900	4.13319700	7.62115200
C	-1.64705900	0.10059400	4.05280900
H	-1.56567000	-0.86084100	4.57301300
H	-2.50988100	0.04984500	3.37360000
H	-0.75344200	0.22093700	3.42422100
C	-2.01619700	3.61748900	5.53803800
C	-2.20541900	5.06794800	5.11428800
H	-3.25324100	5.26382100	4.84088400
H	-1.94121100	5.75633400	5.92732200
H	-1.59813200	5.31684600	4.23538800
C	-0.63297500	2.99486300	2.19997200
C	0.64916900	2.76793300	2.80038900
H	0.69058300	2.51816500	3.86139400
C	1.80941600	2.87777500	2.06633900
C	3.25686800	2.70879700	2.55039600
H	3.32325300	2.42984900	3.60657900
C	3.90987800	1.68208100	1.61434200
C	4.57938700	0.51771300	1.98612600
H	4.69745300	0.27116700	3.03816400
C	5.10960600	-0.32194700	0.99180000
H	5.64496100	-1.22603300	1.27398600
C	4.94732100	-0.00511200	-0.36127400
H	5.34198600	-0.66832600	-1.12684200
C	4.28699700	1.17536800	-0.73492800
H	4.14592000	1.43674600	-1.78153100
C	3.80122900	2.02626300	0.25307000
C	3.11384300	3.37175400	0.01919800
C	3.85738600	4.40165500	0.87693300
C	4.41750400	5.59415200	0.42909800
C	5.10273800	6.41472400	1.33622100
C	5.20163400	6.05242100	2.68176100
C	4.59866500	4.87156100	3.14522400

H	4.65778000	4.59365500	4.19663300
H	5.75519400	6.68819400	3.36935900
H	5.56499600	7.33310300	0.98977600
H	4.31700500	5.88581100	-0.61280200
H	3.06144500	3.65201000	-1.03831200
C	3.93890000	4.04538500	2.23801400
C	1.73959700	3.23156600	0.65996000
C	0.52909900	3.41642900	0.03669700
H	0.49507600	3.65186900	-1.02636800
C	-0.69515200	3.32582900	0.75979400
C	-4.59421500	3.75362300	0.27208300
H	-4.63570600	4.00322000	-0.78896200
B	-2.02449400	3.60277400	0.04462300
C	-2.04580700	3.93688300	-1.50024800
C	-1.92937200	2.90616000	-2.46596400
C	-1.73964800	1.45543600	-2.04331000
H	-2.34626500	1.20588700	-1.16413200
H	-2.00430500	0.76749400	-2.85657600
H	-0.69162800	1.25950400	-1.77074600
C	-1.96456200	3.20988400	-3.83452800
H	-1.92727000	2.39188400	-4.54919800
C	-2.07133800	4.52499900	-4.29872800
C	-2.15610000	5.54527400	-3.34307900
H	-2.23120600	6.57744700	-3.68068500
C	-2.08121500	4.83875000	-5.78543800
H	-2.49100700	4.00138000	-6.36448900
H	-2.68613200	5.72936600	-5.99970600
H	-1.06459400	5.03202000	-6.15983500
C	-2.16308100	5.27199000	-1.96751500
C	-2.29846200	6.42201400	-0.97835600
H	-3.19396300	6.30281300	-0.35216400
H	-1.43730100	6.47020700	-0.29688200
H	-2.37658000	7.38415900	-1.49775100

9. References

(1) Gondo, K.; Kitamura, T. Improved and Practical Synthesis of [2, 4, 5-Tris (Trimethylsilyl)-Phenyl](Phenyl) Iodonium Triflate and Utilization as a 1, 4-Benzdiyne Synthon. *Adv. Synth. Catal.* **2014,** *356*, 2107-2112.

(2) Tsuchiya, Y.; Diesing, S.; Bencheikh, F.; Wada, Y.; Dos Santos, P. L.; Kaji, H.; Zysman-Colman, E.; Samuel, I. D.; Adachi, C. Exact Solution of Kinetic Analysis for Thermally Activated Delayed Fluorescence Materials. *J. Phys. Chem. A* **2021,** *125*, 8074-8089.

(3) Frisch, M.; Trucks, G.; Schlegel, H.; Scuseria, G.; Robb, M.; Cheeseman, J.; Scalmani, G.; Barone, V.; Petersson, G.; Nakatsuji, H. Gaussian 16, Revision A. 03, Gaussian. *Inc., Wallingford CT* **2016,** *3*.

(4) Sun, H.; Zhong, C.; Bredas, J.-L. Reliable Prediction with Tuned Range-Separated Functionals of the Singlet–Triplet Gap in Organic Emitters for Thermally Activated Delayed Fluorescence. *J. Chem. Theory Comput.* **2015,** *11*, 3851-3858.

(5) Tomasi, J.; Mennucci, B.; Cammi, R. Quantum Mechanical Continuum Solvation Models. *Chem. Rev.* **2005,** *105*, 2999-3094.

(6) Chung, L. W.; Sameera, W.; Ramozzi, R.; Page, A. J.; Hatanaka, M.; Petrova, G. P.; Harris, T. V.; Li, X.; Ke, Z.; Liu, F. The Oniom Method and Its Applications. *Chem. Rev.* **2015,** *115*, 5678-5796.

(7) Rappé, A. K.; Casewit, C. J.; Colwell, K.; Goddard III, W. A.; Skiff, W. M. Uff, a Full Periodic Table Force Field for Molecular Mechanics and Molecular Dynamics Simulations. *J. Am. Chem. Soc.* **1992,** *114*, 10024-10035.

(8) Huang, S.; Zhang, Q.; Shiota, Y.; Nakagawa, T.; Kuwabara, K.; Yoshizawa, K.; Adachi, C. Computational Prediction for Singlet-and Triplet-Transition Energies of Charge-Transfer Compounds. *J. Chem. Theory Comput.* **2013,** *9*, 3872-3877.

(9) Jiang, Y.; Hu, Z.; Zhou, B.; Zhong, C.; Sun, Z.; Sun, H. Accurate Prediction for Dynamic Hybrid Local and Charge Transfer Excited States from Optimally Tuned Range-Separated Density Functionals. *J. Phys. Chem. C* **2019,** *123*, 5616-5625.

(10) Neese, F. Software Update: The Orca Program System, Version 4.0. *WIREs Comput Mol Sci.* **2018,** *8*, e1327.

(11) Aidas, K.; Angeli, C.; Bak, K.; Bakken, V.; Bast, R.; Boman, L.; Christiansen, O.; Cimiraglia, R.; Coriani, S.; Dahle, P. Dalton, a Molecular Electronic Structure Program, Release Dalton2016 (2016). *WIREs Comput. Mol. Sci.* **2014,** *4*, 269-284.
